# The Biochemical Pathways of Nicotinamide-Derived Pyridones

**DOI:** 10.3390/ijms22031145

**Published:** 2021-01-24

**Authors:** Faisal Hayat, Manoj Sonavane, Mikhail V. Makarov, Samuel A. J. Trammell, Pamela McPherson, Natalie R. Gassman, Marie E. Migaud

**Affiliations:** 1 Department of Pharmacology, College of Medicine, University of South Alabama, Mobile, AL 36688, USA; fhayat@health.southalabama.edu (F.H.); msonavane@health.southalabama.edu (M.S.); 2 Mitchell Cancer Institute, College of Medicine, University of South Alabama, Mobile, AL 36604, USA; mmakarov@health.southalabama.edu (M.V.M.); pvm1821@jagmail.southalabama.edu (P.M.); nrgassman@southalabama.edu (N.R.G.); 3 Department of Physiology & Cell Biology, College of Medicine, University of South Alabama, Mobile, AL 36688, USA; 4 Novo Nordisk Foundation, Center for Basic Metabolic Research, University of Copenhagen, 2200 Copenhagen, Denmark; trammell@sund.ku.dk

**Keywords:** NAD, redox cofactor, nicotinamide, pyridones

## Abstract

As catabolites of nicotinamide possess physiological relevance, pyridones are often included in metabolomics measurements and associated with pathological outcomes in acute kidney injury (AKI). Pyridones are oxidation products of nicotinamide, its methylated form, and its ribosylated form. While they are viewed as markers of over-oxidation, they are often wrongly reported or mislabeled. To address this, we provide a comprehensive characterization of these catabolites of vitamin B3, justify their nomenclature, and differentiate between the biochemical pathways that lead to their generation. Furthermore, we identify an enzymatic and a chemical process that accounts for the formation of the ribosylated form of these pyridones, known to be cytotoxic. Finally, we demonstrate that the ribosylated form of one of the pyridones, the 4-pyridone-3-carboxamide riboside (4PYR), causes HepG3 cells to die by autophagy; a process that occurs at concentrations that are comparable to physiological concentrations of this species in the plasma in AKI patients.

## 1. Introduction

**Figure 1:**
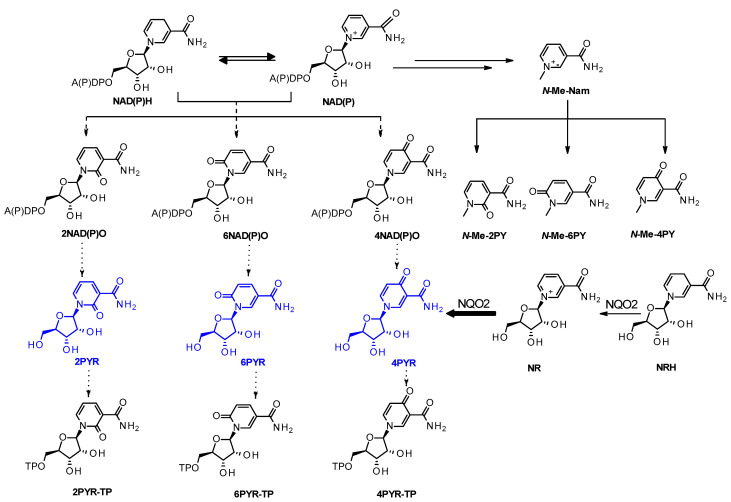
NAD(P)(H)-derived pyridone catabolites. An additional route to the triphosphate from the monophosphate ribosylated pyridone generated from the dinucleotides can also be envisaged. NAD(P)(H): nicotinamide adenine dinucleotide (phosphate) (reduced form); NAD(P)O: hyper- oxidized forms of NAD(P) containing pyridone moieties; PYR-TP: pyridone riboside triphosphate; PYR: pyridone riboside; The 2, 4, and 6 positions of the carbonyl unit are indicated in the abbreviation; *N*-Me-PY: *N*-methyl pyridone.

Through multiple yet convergent biosynthetic pathways, all components of vitamin B3 are precursors to the nicotinamide-derived redox cofactor, nicotinamide adenine dinucleotide (NAD), its reduced form NADH, its phosphate parent NADP, and the reduced form NADPH, collectively referred to as NAD(P)(H) [1]. The water-soluble components that constitute vitamin B3 are obtained through the diet and/or supplementation. Its regular intake must compensate for the daily losses stemming from the degradation of the cofactors [2]. Some of the NAD(P)(H) degradation products (Figure 1) include *N*-methyl-4-pyridone-3-carboxamide (*N*-Me-4PY) and *N*-methyl-6-pyridone-3-carboxamide, often reported as *N*-methyl-2-pyridone-5-carboxamide (*N*-Me-6PY). Both pyridones are detected in plasma and urine as major vitamin B3 degradation products and are oxidized forms of methyl-nicotinamide (Me-Nam) [3,4]. The ribosylated form of the pyridone carboxamides, 4-pyridone-3-carboxamide riboside (4PYR), and 6-pyridone-3-carboxamide riboside also known as 2-pyridone-5-carboxamide riboside (6PYR), are also detected in urine [5]. In blood, these ribosylated pyridones are mostly present intracellularly in their triphosphorylated (4PYR-TP and 6PYR-TP) [6,7] or adenine dinucleotidic forms [8]. *N*-Methyl-2-pyridone-3-carboxamide (Me-2PY) and 2-pyridone-3-carboxamide riboside (2PYR) are the least prominent pyridones in the literature, as they are much less often detected in biological samples [9].

As waste products, these by-products of NAD(P)(H) metabolism associate with aging and nephrotic dysfunction as their levels associate with advanced kidney injuries (AKI) and kidney failure [4,6,10,11,12]. Liquid chromatography combined with mass spectrometry is the most common method used to detect nicotinamide-derived pyridones in biological samples [13,14,15]. However, much confusion arises from their detection, identification, and reporting as additional nicotinamide metabolites that are the non-methylated or ribosylated pyridones are also often mistaken for their methylated species in reports [5,9,11,15,16,17,18]. The misperception stems from confusing nomenclature, compounded by poor quantification due to facile fragmentation and adduct formation under mass spectrometry conditions.

Critically, the chemical nature of each form of the pyridone informs on the type of catabolic pathway the nicotinamide-derived species have undertaken. For instance, only the methylated form of nicotinamide (*N*-Me-Nam) can be the precursor of the three methylated pyridones (*N*-Me-2PY, *N*-Me-4PY, and *N*-Me-6PY, Figure 1) [19,20,21,22]. Similarly, only the ribosylated form of nicotinamide can be the source of the pyridone ribosides [17]. Each of the catabolic routes indicates dysregulation in NAD-dependent metabolism [23,24]. Pyridone ribosides, unlike the methylated species, can be converted to the adenine dinucleotide or the triphosphate form [17,25]. The 4PYR isomer not only circulates in plasma once generated but is also internalized and metabolized to species that interfere with enzymatic processes [26,27,28]. Possessing a systematic, reliable account of the pyridones’ distribution would greatly help identify whether these species are pathophysiologically significant.

Here, we present a comprehensive comparative characterization of each species and explore the mechanisms by which their under-detection and misidentification can arise. We also propose a mechanism for the wide-spread production of the ribosylated pyridone 4PYR and rationalize selective cytotoxicity by comparing how exogenous exposure to ribosylated pyridones affects cell survival.

## 2. Results

**Scheme 1:**
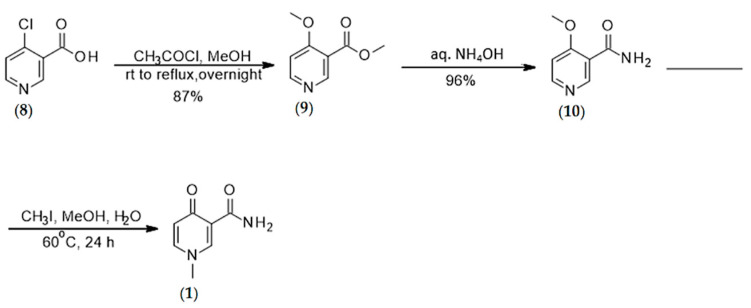
Synthesis of *N*-methyl-4-pyridone 3-carboxamide.

**Scheme 2:**
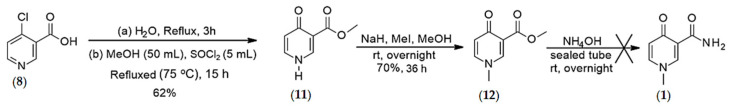
Synthesis of the *O*-methyl, 1-*N*-methyl-4-oxo-pyridine-3-carboxylate (**12**).

**Scheme 3:**
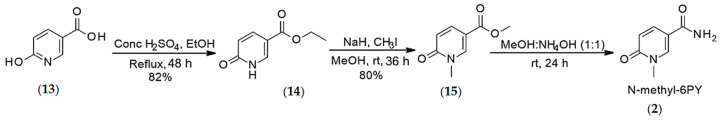
Synthesis of *N*-methyl-6-pyridone 3-carboxamide.

**Scheme 4:**
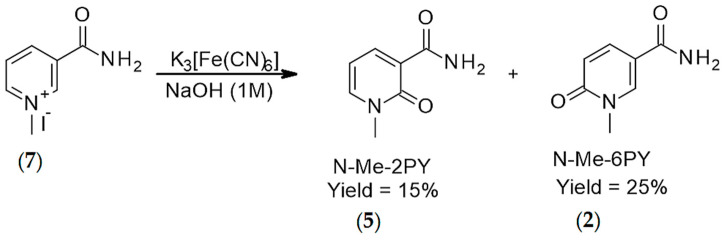
Synthesis of 1-*N*-methyl-2-pyridone-3-carboxamide and 1-*N*-methyl-6-pyridone-3-carboxamide.

**Scheme 5:**
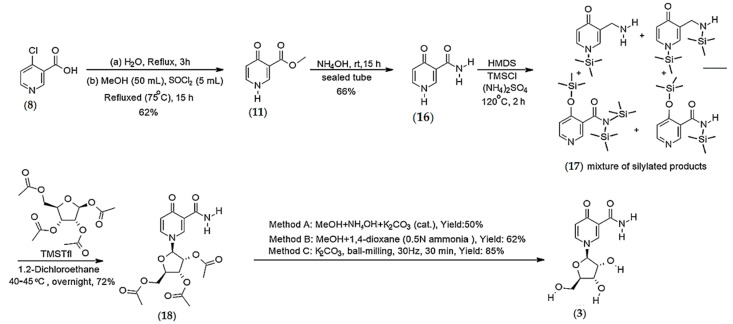
Synthesis of 4-pyridone-3-carboxamide riboside.

**Scheme 6:**
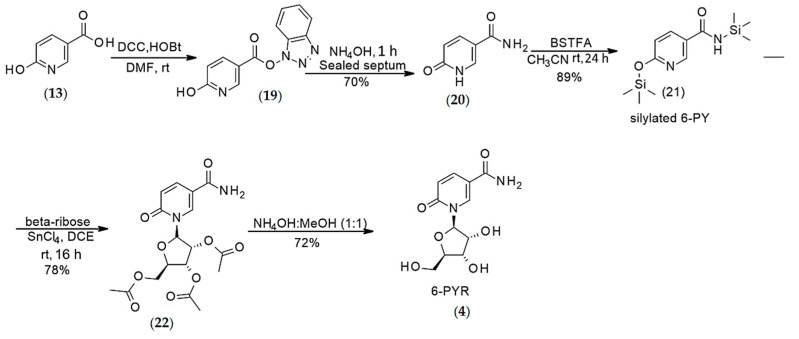
Synthesis of 6-pyridone 3-carboxamide riboside.

**Scheme 7:**
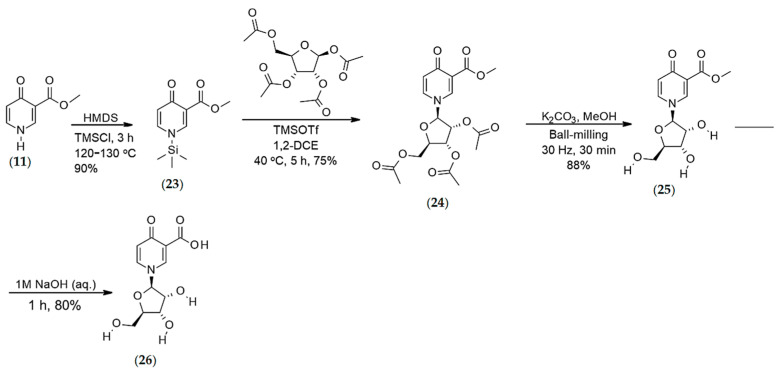
Synthesis of the methyl-ester and carboxylate ribosyl analogs.

**Scheme 8:**
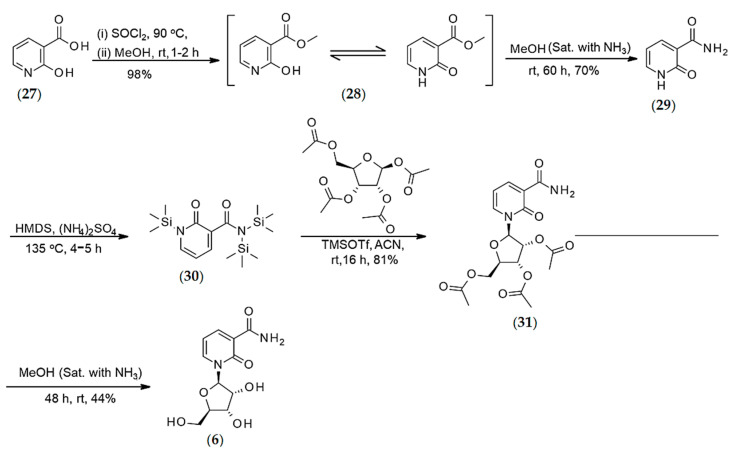
Synthesis of the 2-pyridone-3-carboxamide riboside.

The *N*-methylated 6-pyridone were easily generated from their respective chloronicotinic acid precursors as were the ribosylated 6-pyridone according to published syntheses [6,29,30,31,32]. However, *N*-Me-2PY, 2PYR, *N*-Me-4PY, and 4PYR converted readily to the methylated ester instead of the amide under aqueous methanolic conditions. Alterations to the published synthetic sequences were deemed necessary to ensure sample authenticity and are described in detail in the experimental and methods section (Scheme 1, Scheme 2, Scheme 3, Scheme 4, Scheme 5, Scheme 6, Scheme 7 and Scheme 8 in Methods). All synthetic compounds were purified by separation by flash column, and their chemical structures were fully characterized by both NMR and mass spectrometry (Supplementary Material Table S1 and Spectra). More details on structural characterization can be found in the Supplementary Material Section. In Table 1, Table 2 and Table 3, we have compiled the ^1^H NMR experimental data for the pyridones (Table 1), the methylated pyridones (Table 2), and the ribosylated pyridones (Table 3), while a comprehensive mass spectrometry report can be found in the Supplementary Material Section. The ^1^H NMR of each one of the pyridone is very characteristic of each species according to the position of the carbonyl moiety as well as the type of substitution present on the heterocycle.

The simple 4PY was synthesized in a three-step process as reported by Slominska et al [25]. First, 4-chloronicotinic acid was hydrolyzed then converted to the acyl chloride with thionyl chloride in MeOH solution. The 4-pyridone-formylchloride was treated with aq.NH_4_OH in a sealed tube to generate 4PY. For the 6PY, 5-hydroxy-nicotinic was activated with HOBt in DMF and subsequently treated with aq.NH_4_OH in a sealed tube at rt to afford 6PY in 70% yield. Finally, 2PY was generated from 2-hydroxy-nicotinic acid, which following its conversion to the acyl halide using SOCl_2_ reagent was converted to the amide using aq.NH_4_OH in a sealed tube at rt. When stirred with aq. MeOH, the acyl chloride hydrolyzes to its nicotinate salt that exists in both keto and enol forms.

The synthesis of ribosylated forms of 2PY, 4PY, and 6PY (2PYR, 4PYR, and 6PYR, respectively) is based on Vorbrüggen glycosylation reaction [31,32]. Following silylation of the nucleobase using either HMDS (2-PY and 4-PY) or BSTFA (6-PY), the silylated pyridones were mixed with beta-d-riboside tetraacetate in the presence of a Lewis acid. For the ribosylation of 2PY and 4PY, TMSOTf in 1,2-DCE/acetonitrile was applied, while SnCl_4_ was used for the ribosylation of 6PY. While the deacetylation should have been straightforward, it had to be optimized for 2PYR and 4PYR to prevent the loss of the amine moiety and generation of the corresponding carboxylic acid derivatives, materials that we have also fully characterized for reasons described below. Finally, the *N*-methyl derivatives of 4PY and 6PY (*N*-Me-2PY, *N*-Me-4PY, and *N*-Me-6PY, respectively) were prepared by methylation with methyl iodide. *N*-methyl-2-pyridone, *N*-Me-2PY was obtained by oxidation of *N*-methyl nicotinamide with K_3_Fe(CN)_6_ under basic conditions [33,34].

**Figure 2:**
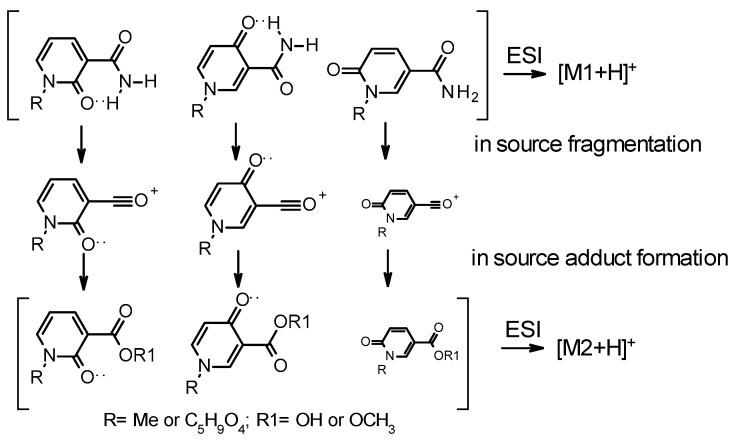
Pyridone carboxamides, their ESI-induced fragment, and the subsequent in source generated adduct. The contribution of in-source fragmentation of the *N*-Me-6PY is thought to be minimal.

As described above, *N*-methyl-nicotinamide oxidizes to three possible forms, *N*-Me-2PY, *N*-Me-4PY, and *N*-Me-6PY. By mass spectrometry (ESI-MS, Supplementary Material Table S1), it was found that the three isomers fragment were similar to each other but differ in the relative abundance of the predominant fragmentation products (Supplementary Material Table S1). The fragmentation pattern of each pyridone derivative is a fingerprint of that pyridone and can be used to identify these species unambiguously (Supplementary Material Spectra). Crucially, the functional group exchange between the amide and the carboxylic acid described above is observed under simple electrospray conditions (Supplementary Material Table S1 and Spectra). All three methylated pyridone forms (*N*-Me-2PY, *N*-Me-4PY, and *N*-Me-6PY) are deamidated on the carboxamide to form an ion at *m/z* 136. However, this fragment is the predominant product at low energy when fragmenting *N*-Me-2PY, and *N*-Me-4PY but more minor when fragmenting *N*-Me-6PY. The same fragmentation profile is observed for 2PYR, 4PYR, and 6PYR (Supplementary Material Table S1). Critically, when the mass spectrometry analyses are conducted in the presence of water and/or methanol, the ionized carbonium fragment (Figure 2) can react with in situ solvents to generate adducts that in turn get ionized and thus get detected. We have provided a comparative characterization of these materials to address the possible mischaracterization of these metabolites by MS. While not endogenously generated, they can be major contributors to the pyridone pool they originated from.

While the origins of the methylated pyridones (*N*-Me-2PY, *N*-Me-4PY, and *N*-Me-6PY) have been attributed to the oxidation of *N*-methyl nicotinamide by aldehyde oxidases and xanthine oxidases [35,36,37], the formation of the pyridone ribosides remains more elusive. Interestingly, side reactions between water and NADP in the binding pocket of adrenodoxin reductase (FDXR) have been reported [38,39,40]. The nucleophilic addition of water on the ribosylated nicotinamide moiety of NADP was thought to promote the formation of 4NADPO. We explored the possibility that nicotinamide riboside, NR, and its reduced form, NRH are oxidized by the FAD-dependent reductase, dihydronicotinamide riboside: quinone reductase 2 via a similar mechanism, using LC-MS combined to mass spectrometry. The oxidation of NRH by the FAD-dependent NQO2 was monitored at 340 nM by LC-UV-spectroscopy, while the appearance of NR was confirmed by monitoring at 254 nm. Similarly, when seeking to oxidize NR, the reaction was monitored at 254 nm to follow NR consumption. We observed not only that NRH was oxidized to NR in the presence of FAD and menadione, but that the pyridone riboside, 4PYR, was also generated over time, as confirmed by LC-MS-MS. This chemistry was not observed when NR was used as the starting material. This indicates that the mechanism responsible for the oxidation of NRH to 4PYR by NQO2 is not the same as the mechanism observed and characterized for the oxidation of NADP by adrenodoxin reductase.

**Figure 3:**
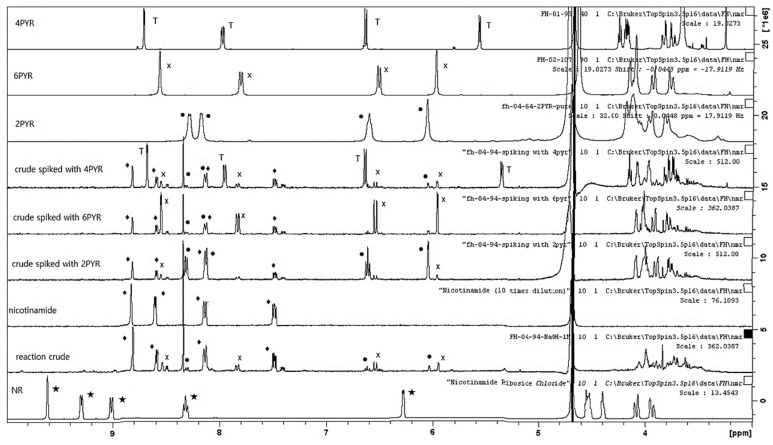
Fenton chemistry facilitates the formation of 2PYR and 6PYR from NR. Overlay ^1^H NMR of nicotinamide riboside (bottom spectrum) and crude reaction mixture (above). NR decomposes to nicotinamide under basic conditions (3rd spectrum up). The crude reaction mixture was spiked with each pyridone ribosides (4th–6th spectra). Pure PYR ^1^H NMR spectra (7th–9th spectra). Only 2PYR and 6PYR are present in the reaction mixture. ● 2PYR; x 6PYR; T 4PYR; ★ Nicotinamide Riboside; ♦ Nicotinamide.

Seeking to establish the mechanism of 4PYR formation by NQO2, we turned to Fenton Chemistry. In the presence of K_3_(Fe(CN)_6_) in 1 M NaOH, methyl-nicotinamide is readily oxidized, and *N*-Me-2PY and *N*-Me-6PY were detected. A carboxylic acid [C_7_H_7_NO_3_+H^+^] was also detected by direct ESI-MS. This outcome could be consistent with the synthetic challenges we experienced in the synthesis of *N*-Me-4PY. When we applied this chemistry to nicotinamide riboside (NR), we detected 2PYR [41] and 6PYR [42,43], the two isomers of 4PYR (Figure 3), along with nicotinamide, the product formed from NR hydrolysis. These outcomes indicate that the NQO2-catalyzed over-oxidation of NRH favors one isomer, 4PYR, and occurs via a mechanism that differs from Fenton chemistry as it is more regioselective. It is also different from adrenodoxin reductase (FDXR) oxidation of NADP, as the over-oxidation of NRH by NQO2 requires the initial release of a hydride for superoxide formation via FAD.

Acetaminophen, also known as paracetamol is oxidized to a quinone by the FDXR-dependent cytochrome CYP450, CYP2E1, for which acetaminophen administration stimulates the overexpression [44]. acetaminophen exposure also promotes the overexpression of NADPH: quinone oxide-reductase 1 (NQO1) [45], an homolog of NQO2, which catalyzes the reduction of the quinone back to acetaminophen. Catabolites of vitamin B3 found in urine include nicotinuric acid, trigonelline, methyl-nicotinamide, Me-2PY, Me-4PY, and 4PYR. In a human preliminary study, we measured the levels of Me-Nam, *N*-Me-4PY, and 4PYR in urine over a 24 h period and compared these levels with those obtained following the ingestion of 1 gm of acetaminophen (Supplementary Figure S3). We observed that acetaminophen consumption increases the urinary levels of 4PYR by more than 40-fold but does not change the levels of the non-ribosylated species. This observation would indicate that acetaminophen only promotes the oxidation of the ribosylated forms of nicotinamide, i.e., NR, NMN, or NAD(P) and this via a mechanism which is independent of the aldehyde oxidase, the enzyme responsible for the generation of *N*-Me-2PY and *N*-Me-4PY from methyl-nicotinamide in vivo.

**Figure 4:**
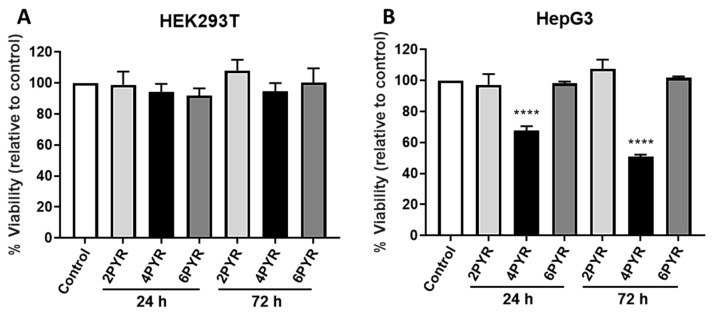
4PYR is cytotoxic to HepG3 cells but not HEK293T. Cell viability after 72 h was assessed for HEK293T (**A**) and HepG3 (**B**) cells. Cells were exposed to 100 µM of 2PYR, 4PYR, or 6PYR for 24 h with pyridones removed and fresh medium replaced for an additional 48 h or continuously exposed to the pyridone isomers for 72 h. After 72 h, CellTiter-Fluor^TM^ viability assay was performed with results expressed as the mean fluorescence intensity relative to control, vehicle-treated cells (% Viability) ± standard error of the mean (SEM). Statistical significance: **** *p* < 0.0001.

The studies evaluating the PYR-family cytotoxicity are somewhat confusing and imprecise, as not all cell-lines appear to be sensitive to exposure to these nucleosides [26,46]. Given the literature, we decided to explore which one if not all the pyridone ribosides were cytotoxic to two very metabolically different cell lines, the human embryonic kidney HEK293T cell line and the human hepatocarcinoma HepG3 cell line. Compared to HEK293T that is more glycolytic, HepG3 is more reliant on mitochondrial respiration for energy production, processes that are NAD(P) dependent and therefore potentially sensitive to the presence of PYR-derived dinucleotides. Using a CellTiter-Fluor^TM^ viability assay as a reporter [47], cytotoxicity assays on HEK293T and HepG3 cell cultures indicated that none of the PYR-derivates were cytotoxic to HEK293T cells but that the 4PYR reduced the cell viability of HepG3 cells by 62% after 24 h of treatment at 100 µM followed by 48 h recovery in cell medium and by 45% after 72 h of continuous exposure (Figure 4).

**Figure 5:**
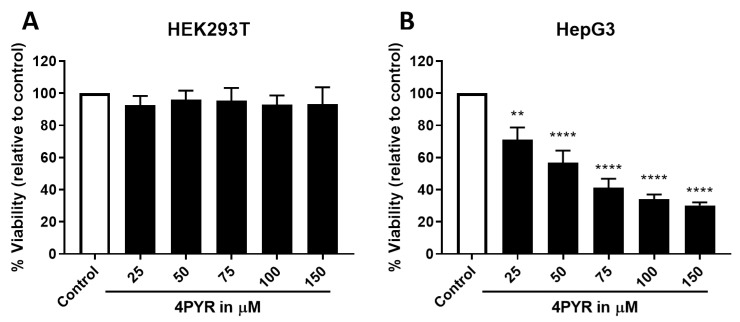
Dose-dependent sensitivity of HepG3 cells to continuous 4PYR treatment. HEK239T (**A**) and HepG3 (**B**) cells were exposed to 25, 50, 75, 100, and 150 µM continuously for 72 h. CellTiter-Fluor^TM^ Viability assay was performed with results expressed as the mean fluorescence intensity relative to control, vehicle-treated cells (% viability) ± SEM. Statistical significance: ** *p* < 0.005, **** *p* < 0.0001.

**Figure 6:**
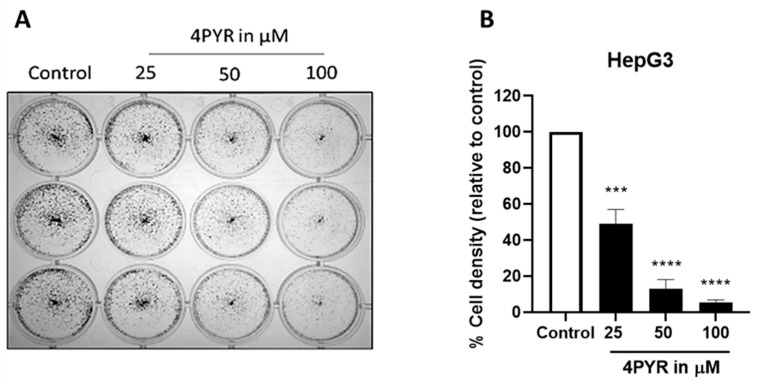
Clonogenic survival of HepG3 cells continuously exposed to 4PYR. HepG3 cells were exposed to 25, 50, and 100 µM of 4PYR for six days then stained with crystal violet (**A**). Cell density was assessed by measuring the area fraction of the crystal violet-stained cells after 4PYR exposure (**B**). Results are presented as percentage cell density to control ± SEM of three independent experiments. Statistical significance: *** *p* < 0.001, **** *p* < 0.0001.

**Figure 7:**
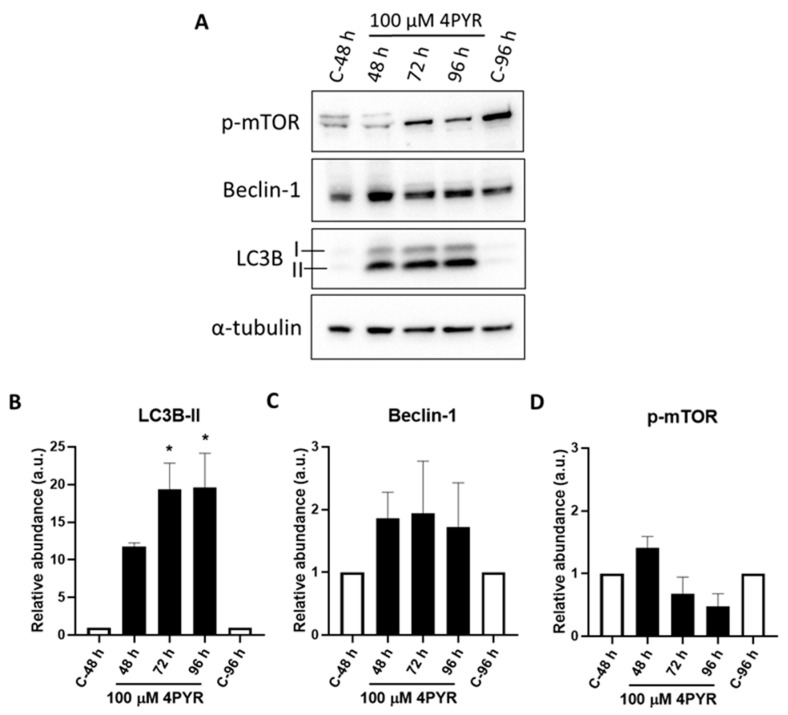
4PYR induced autophagy in HepG3 cells. HepG3 cells were continuously dosed with 100 µM of 4PYR for 48, 72, and 96 h. (**A**) Increased protein expression levels of the autophagy marker LC3BII, Beclin-1, and decrease in phospho-mTOR were assessed using immunoblot in 4PYR treated HepG3 cells compared to vehicle-treated control cells. α-tubulin was used as a loading control for immunoblotting. The graph shows quantified protein expression levels relative to controls for (**B**) LC3B-II, (**C**) Beclin-1 and (**D**) phosphor-mTOR in HepG3 cells. Results are expressed as relative abundance (a.u.) ± SEM of two biological replicates. Statistical significance: * *p* < 0.05.

A dose-dependent sensitivity assay of HepG3 cells exposed to continuous 4PYR treatment showed that 4PYR was cytotoxic to HepG3 in a dose-dependent manner with an IC50 at 50 µM (Figure 5). Crucially, even at higher concentrations in this cell line, surviving cells remain, and the loss of cell viability appears to plateau. We also characterized the effects of 4PYR on HepG3 cells in a clonogenic survival assay, with HepG3 cell density less than 50% after 6 days at 25 µM of 4PYR (Figure 6). Together, these data provided some indications of the possible mode of action of 4PYR in HepG3 cells, which likely promotes senescence in the remaining viable cells. Therefore, we sought to establish whether markers of autophagy could be detected in 4PYR-treated HepG3. Immunoblotting showed an increase in protein expression levels of the autophagy marker LC3BII, Beclin-1, and a decrease in phospho-mTOR compared to vehicle-treated control cells (Figure 7). Furthermore, we confirmed that apoptosis was not observed over the same period (data not shown).

**Figure 8:**
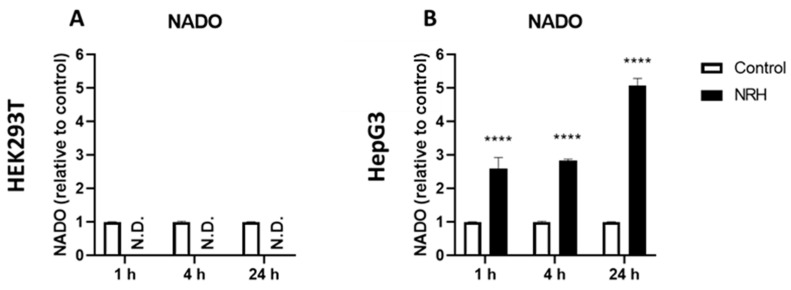
NRH induced 4NADO production in HepG3 cells but not in HEK293T. HEK293T (**A**) and HepG3 (**B**) cells were continuously dosed with 100 µM of 4PYR for 1, 4, and 24 h. The crude cell extract was measured by LC-MS for its NADO content. The NADO detected matches the 4NADO standard. Results are presented as relative abundance to control ± SEM of three independent experiments. Statistical significance: **** *p* < 0.0001. N.D: Not Detected.

Finally, we exposed HEK293T and HepG3 cells to 100 μM NRH, the reduced form of nicotinamide riboside, for 1, 4, and 24 h and measured the cell extract for the presence of the over-oxidized form of NAD, NADO by LC-MS. While HEK293T did not produce NADO under such conditions, HepG3 cells produced 4NADO intracellularly, and its abundance increased with the length of exposure (Figure 8).

## 3. Discussion

Pyridones and methyl-nicotinamide are measured in clinical settings to identify vitamin B3 deficiencies [48,49] and in metabolomics as they associate with increased NAD consumption [50,51] and cellular dysfunctions in tissues [51,52,53,54,55]. Unfortunately, over the years the nomenclature used to describe the pyridone’s nature thus characterized has been somewhat unsystematic and exquisitely confusing (e.g., [5,11,56]). For instance, the term “pyridone” can describe both the methylated or non-methylated forms, abbreviated PY. Similarly, PY and PYR have been used to describe the ribosylated or non-ribosylated species indiscriminately (e.g., [28,57]). Surely, there is a need for consistency, as these species come from very distinct pathways and are sought systematically during physiological and pathophysiological investigations [11,55]. We proposed that by implementing the IUPAC nomenclature, inconsistencies can be readily rectified. We have named, characterized, and compared each of the pyridone species to facilitate this transition to provide clarity to the field.

The abundance of urinary pyridones derived from nicotinamide has been shown to correlate with metabolic syndromes and aging [4,58]. While *N*-Me-2PY, *N*-Me-6PY, *4*PYR, and 6PYR are detected in human urine, *N*-Me-2PY and 2PYR could go undetected because of their rapid conversion to the free acid or an ester during acidic or basic extraction protocols [59,60,61]. When the amide bond can share hydrogen bond interactions with the neighboring carbonyl, as is the case in *N*-Me-4PY, 4PYR, *N*-Me-2PY, and 2PYR, the loss of ammonia occurs readily, as we observed in solution chemistry, a process that could readily occur during sample processing.

Mass spectrometry detects all metabolites in ionic forms. For the NAD metabolome, in some cases, such as for NR, the metabolite exists in an ionic form before reaching the detector; however, in most cases, the metabolite requires protonation/deprotonation or adduction, usually with sodium, potassium, chloride, ammonium. Many metabolites included in the NAD metabolome react to the most common ionization source, electrospray ionization (ESI) [62,63]. ESI employs a steady stream of nitrogen and heat to produce ions. *N*-Me-4PY, 4PYR, *N*-Me-2PY, and 2PYR react within the ESI chamber in a potentially confounding way because of an MS/MS fragmentation and formation of an acylium ion. In the ESI-MS instrument, such fragments, if stabilized or produced at a high enough rate, can react with water or methanol to form the acid or methyl ester analogs of *N*-Me-4PY, 4PYR, *N*-Me-2PY, and 2PYR (Figure 2). Here, the in-source reaction can lead to a loss of signal and potentially the false identification of metabolites that likely do not exist in the biological sample. Furthermore, metabolite derivatives, artifacts of ESI, could go unmeasured. When a low-resolution instrument is used, the carboxylic acids may be non-resolved from the carboxamide form (Supplementary Materials). However, high-resolution mass spectrometers are more than capable of resolving the amide form from the carboxylic acid [64]. In quantitative metabolomics, internal isotopically labeled standards are used to identify and quantify specific metabolites of interest [63]. In a more encompassing but less accurate, semi-quantitative metabolomics, identifying a species often relies upon the *m/z* ratio and fragmentation. As such, the materials can go undetected or underrepresented unless dedicated internal standards are used for their quantification. It is with this in mind that one can rationalize the often-contradictory reports of pyridones’ quantifications.

Crucially, the ribosylated pyridones derived from nicotinamide can only be generated from the ribosylated forms of nicotinamide [17]. Such forms include nicotinamide riboside (NR), its mononucleotide parent (NMN) or NAD(P), and their reduced forms [1]. Circulating endogenous and exogenous 4PYR is readily intracellularized and metabolized by blood and tissues where it is phosphorylated and converted to either 4PYR-triphosphate (4PYR-TP) or the NAD derivative 4NADO, all inhibitors of intracellular enzymes [25,27,46,65].

The cells’ ability to convert exogenous pyridone ribosides to NAD-like species might select for 4PYR’s capacity to promote cell death in a cell-specific manner, as it has been observed here and by others [26,28]. Crucially, we observed similar cell line selectivity regarding cell survival when the adenine dinucleotide form of 4PYR, 4NADO, is generated endogenously following exposure to the reduced form of NR, NRH (Figure 8) [47]. Still, the causality of cell death observed in HepG3 cells exposed to NRH, and the formation of 4NADO remain to be explored. Finally, exogenous 2PYR and 6PYR do not affect HEK293T and HepG3 cells’ viability (Figure 6). Yet, their function, if generated endogenously, remains unknown. Therefore, the physiological properties of endogenously produced toxins derived from the oxidation of NAD, NADP, and NR warrant further investigations.

Finally, aldehyde oxidases are responsible for the formation of the methylated pyridones (*N*-Me-6PY, and *N*-Me-4PY) from methyl-nicotinamide [36]. Yet, the generation of the three ribosylated pyridone derivatives, 2PYR, 4PYR, and 6PYR, remains unexplained. A hint as to the source of the ribosylated pyridones was obtained when the FAD (flavin adenine dinucleotide)-dependent FDXR was shown to generate the oxidized form of NADP, NADPO, in a side-reaction. In the proposed mechanism, NADP, instead of the normal substrate NADPH, binds FAD-bound FDXR [39]. The 1,4-nucleophilic addition of an active site water molecule on the enzyme-bound NADP leads to a 4-hydroxylated dihydropyridine derivative that can then enter in hydride-transfer type catalysis with the bound FAD to generate the 4-pyridone form of NADP (i.e., 4NADPO). At first sight and given the abundance of the urinary ribosylated pyridones, the kinetics of this side-reaction enzymatic process would preclude it to be the major source of 4PYR. Furthermore, this enzymatic process only accounts for the generation of the dinucleotide form of 4PYR and cannot explain the formation of 2PYR, known to also be in circulation [66]. NQO1 and NQO2 are FAD-dependent redox enzymes that bind NAD(P)H and NRH, respectively, and could potentially generate 4NAD(P)O and 4PYR via a mechanism like that of FDXR. Recent work on NQO2 and acetaminophen in the presence of NRH has identified superoxide HOO- as a by-product of the NQO2-catalyzed turn-over, especially in the presence of O_2_ [45]. We envisage that the detection of 4PYR in the NQO2-catalyzed oxidation of NRH is promoted by the NQO2-catalyzed production of superoxide upon the reduction of NRH, with O_2_ as a co-oxidant and that the production of superoxide within the vicinity of a still FAD-NQO2-bound NR, favors the addition of superoxide onto the electrophilic NR. Superoxide is likely to react within the constraints of the enzyme binding pocket and lead to a favored isomer. As such, this type of side reaction or a variation thereof could apply to other NAD(P)H binding FAD-dependent oxidoreductases and explain the generation of a substantial amount of ribosylated pyridone metabolites in vivo. It is important to note that CYP450 and FAD-dependent aryl hydroxylation has long been recognized as a source of hydroxylated catabolites of aryl amino acids and riboflavin, e.g., 2/3-hydroxylated tyrosine and 7/8-hydroxyriboflavins, respectively [67,68]. In such a process, the transient formation of the ROS species is responsible for the outcomes of the conversion. We, therefore, explored whether Fenton chemistry [69], often used to emulate ROS-chemistry in these systems, could promote PYR formation from NR and found that only 2PYR and 6PYR but not 4PYR were readily detected. We have therefore identified two related oxidative mechanisms that could be wide-spread and responsible for the formation of functionalized ribosylated pyridones.

## 4. Methods

### 4.1. Syntheses

#### 4.1.1. Synthesis of *N*-methyl-4-pyridone 3-carboxamide (**1**)

The synthesis of *N*-methyl-4-pyridone 3-carboxamide was conducted according to Scheme 1.

*Synthesis of O-methyl 4-methoxypyridine-3-carboxylate* (**9**) *(Step-1)*: To a solution of 4-chloronicotinic acid (**8**) (1.0 g, 6.34 mmol) in anhydrous methanol (18.5 mL) was added acetyl chloride (1.42 mL) at 0 °C. The reaction mixture was stirred under nitrogen overnight at 65–70 °C. Upon completion of the reaction, the reaction mixture was concentrated under reduced pressure. The crude was dissolved in saturated NaHCO_3_ (pH = 8–9) and then extracted with CHCl_3_ (2 × 15 mL). The combined organics were washed with brine (30 mL), dried over Na_2_SO_4_, filtered, and concentrated under reduced pressure to afford the desired product as a white solid (87%). ^1^H NMR (MeOD), δ, ppm: 8.74 (s, 1H, H1), 8.51 (d, J = 6.04 Hz, 1H, H6), 7.18 (d, J = 6.04 Hz, 1H, H5), 3.96 (s, 3H, COOMe), 3.87 (s, 3H, –OMe). ^13^C NMR (MeOD), δ, ppm: 165.58 (C4), 164.92 (C7), 153.52 (C2), 151.37 (C6), 116.45 (C3), 107.93 (C5), 55.33 (C9), 51.31 (C8) [29].

*Synthesis of 4-methoxypyridine-3-carboxamide* (**10**) *(Step-2)*: *O*-Methyl-4-methoxypyridine-3-carboxylate (**9**) (0.5 g, 2.99 mmol) in NH_4_OH (27%, 20 mL) was stirred at rt for 15 in a 50 mL sealed tube and the reaction was monitored by ^1^H NMR. After the reaction had gone to completion, the solvent was evaporated under reduced pressure and the obtained white solid was used for the next step without purification (96%). ^1^H NMR (MeOD), δ, ppm: 8.87 (s, 1H, H1), 8.51 (d, J = 5.96 Hz, 1H, H6), 7.21 (d, J = 6.0 Hz, 1H, H5), 4.04 (s, 3H, –OMe). ^13^C NMR (MeOD), δ, ppm: 166.50 (C4), 164.35 (C7), 153.02 (C2), 151.37 (C6), 118.16 (C3), 107.51 (C5), 55.55 (C8) [29].

*Synthesis of 1-*N*-methyl-4-oxo-pyridine-3-carboxamide* (**1**) *(Step-3)*: To a solution of 4-methoxypyridine-3-carboxamide (**10**) (438 mg, 2.88 mmol) in methanol:water (9:1) mixture (10 mL) was added iodomethane (1.74 mL, 28 mmol). The reaction was stirred under nitrogen for 24 h at 60 °C. After stirring for 24 h at 60 °C with an oil bath, the mixture was cooled in an iced bath for 1 h and the resulting yellow precipitate was filtered off, washed with methanol, and dried under high vacuum. The final product did not require purification. Yield: 65%. ^1^H NMR (DMSO, d^6^), δ, ppm: 9.36 (s, 1H, NH), 8.54 (s, 1H, H2), 7.87 (d, J = 5.08 Hz, 1H, H6), 7.54 (s, 1H, NH), 6.53 (d, J = 7.4 Hz, 1H, H5), 3.82 (s, 3H, Me). ^13^C NMR (MeOD), δ, ppm: 175.66 (C4), 165.51 (C7), 146.51 (C2), 143.27 (C6), 119.66 (C3), 118.97 (C5), 44.37 (C8), HRMS calcd for C_7_H_9_N_2_O_2_ [M + H]^+^ 153.0664 found 153.0652 [29].

#### 4.1.2. Synthesis of *O*-methyl, 1-*N*-methyl-4-oxo-pyridine-3-carboxylate (**12**)

The synthesis of *O*-methyl, 1-*N*-methyl-4-oxo-pyridine-3-carboxylate was conducted according to Scheme 2.

*Synthesis of O-methyl 4-pyridone-3-carboxylate* (**11**) *(Step-1)*: To a solution of the 4-chloronicotinic acid (**8**) (5 g, 31.7 mmol) in H_2_O (50 mL) was refluxed for 3 h and then concentrated under reduced pressure. The obtained yellow solid was azeotroped with toluene and then dissolved in anhydrous MeOH (50 mL). To the resulting mixture was added SOCl_2_ (4.99 mL, 8.19 g, 68.9 mmol) at room temperature, and then the reaction mixture was heated to 75 °C. After stirring at 75 °C for 15 h, the mixture was concentrated under reduced pressure, and the residual product was treated with saturated NaHCO_3_. As the remaining amount of thionyl chloride got neutralized by NaHCO_3_, the desired product precipitated. The precipitate was filtered, washed twice with water, and used directly for the next step without further purification. E.g., Scheme Yield 62%. ^1^H NMR (D_2_O), δ, ppm: 8.44 (s, 1H, H2), 7.76 (d, J = 7.2 Hz, 1H, H6), 6.53 (d, J = 6.53 Hz, 1H, H5), 3.77 (s, 3H, OMe). ^13^C NMR (CDCl_3_), δ, ppm: 178.34 (C4), 166.77 (COOCH3), 144.41 (C2), 139.15 (C6), 120.11 (C5), 117.13 (C3), 52.26 (Me), HRMS calcd for C_7_H_8_NO_3_ [M + H]^+^ 154.0504 found 154.0490. Spectral data are consistent with published literature [25].

*Synthesis of O-methyl, 1-*N*-methyl 4-pyridone-3-carboxylate* (**12**) *(Step-2)*: In a 50-mL round bottom flask, equipped with a magnetic stirring bar, NaH (15.30 mg, 0.65 mmol) was added to 2 mL of anhydrous methanol in a portion-wise manner, and then methyl 4-pyridone-3-carboxylate (**11**) (50 mg, 0.33 mmol) was added. The resulting reaction mixture was stirred at rt for 10 min. Then, CH_3_I (81.17 µL, 1.30 mmol) was added, and the resulting mixture was stirred again at the same temperature for 36 h. The reaction progress was monitored by TLC. Upon completion of the reaction, the resulting mixture was concentrated under reduced pressure, and the desired product was precipitated by adding EtOAc (2 mL). (Yield = 70%). ^1^H NMR (MeOD), δ, ppm: 8.47 (s, 1H, H2), 7.72 (dd, J = 2.28 & 2.28 Hz, 1H, H6), 6.49 (d, J = 7.52 Hz, 1H, H5), 3.83 (s, 3H, OMe), 3.80 (s, 3H, –NMe). ^13^C NMR (CDCl_3_), δ, ppm: 176.38 (C4), 164.88 (C7), 147.58 (C2), 142.16 (C6), 120.79 (C3), 117.26 (C5), 50.86 (–OCH_3_), 43.23 (–CH_3_). HRMS calcd for C_8_H_10_NO_3_ [M + H]^+^ 168.0661 found 168.0648. Spectral data are consistent with published [M^+^ + H].

#### 4.1.3. Synthesis of *N*-methyl-6-pyridone 3-carboxamide (**2**)

The synthesis of *N*-methyl-6-pyridone 3-carboxamide was conducted according to Scheme 3.

*Synthesis of O-ethyl 6-oxo-1H-pyridine-3-carboxylate* (**14**) *(Step-1)*: To a stirred solution of 6-hydroxynicotinic acid (**13**) (5 g, 3.59 mmol) in absolute ethanol (25 mL) was added sulfuric acid (0.2 mL) at room temperature. The mixture was heated to reflux for 48 h. After cooling down to room temperature, water (2.5 mL) was added, and the reaction mixture was neutralized to pH = 6–7 by portion-wise addition of sodium hydrogen carbonate (482 mg). Upon completion of the reaction, the reaction mixture was concentrated under reduced pressure, and the residue was extracted with ethyl acetate (3 × 10 mL). The combined organic layers were washed with brine, dried over Na_2_SO_4_, and evaporated under reduced pressure leading to the pure ethyl 6-hydroxynicotinate (**14**) (yield = 82%). ^1^H NMR (CDCl_3_), δ, ppm: 8.16 (s, 1H, H6), 7.95 (dd, J = 2.44 & 2.44 Hz, 1H, H4), 6.51 (d, J = 9.56 Hz, 1H, H3), 4.25 (q, J = 7.14 Hz, 2H, -CH2), 1.28 (t, J = 7.12 Hz, 3H, Me). ^13^C NMR (CDCl_3_), δ, ppm: 165.61 (COCH_3_), 164.00 (C2), 141.11 (C6), 139.69 (C4), 119.36 (C3), 111.44 (C5), 60.68 (–CH_2_), 14.23 (Me) [30].

*Synthesis of O-ethyl-1-methyl-6-oxo-pyridine-3-carboxylate* (**15**) *(Step-2)*: In a 50-mL round bottom flask, equipped with a magnetic stirring bar, NaH (56.11, 2.39 mmol) was added to 3 mL of anhydrous methanol in a portion-wise manner, and then ethyl 6-hydroxynicotinate (**14**) (0.2 g, 1.19 mmol) was added, the resulting reaction mixture was stirred at rt for 10 min, and then CH_3_I (0.297 mL, 4.78 mmol) was added, and the resulting mixture was stirred again at the same temperature for 36 h. The reaction progress was monitored by TLC analysis. Upon completion of the reaction, the resulting mixture was concentrated under vacuum and dissolved in EtOAc (15 mL). The organic layer was washed with water (3 × 10 mL), dried over Na_2_SO_4_, and concentrated under reduced pressure to afford the product (**15**) as white solid (Yield = 80%). ^1^H NMR (CDCl_3_), δ, ppm: 8.12 (s, 1H, H6), 7.77 (dd, J = 2.52 & 2.52 Hz, 1H, H4), 6.46 (d, J = 9.52 Hz, 1H, H3), 3.79 (s, 3H, OMe), 3.52 (s, 3H, Me). ^13^C NMR (CDCl_3_), δ, ppm: 165.61 (COCH_3_), 162.89 (C2), 143.55 (C6), 138.63 (C4), 119.40 (C3), 108.58 (C5), 52.04 (OMe), 38.20 (Me). HRMS calcd for C_8_H_10_NO_3_ [M + H]^+^ 168.0661 found 168.0647.

*Synthesis* of 1-*N-methyl-6-pyridone-3-carboxamide* (**2**) *(Step-3)*: A sealed tube equipped with a Teflon-coated magnetic stirring bar was charged with *O*-methyl, 1-*N*-methyl-6-oxo-pyridine-3-carboxylic acid (**15**) (2 g, 13.0 mmol) and 10 mL mixture of NH_4_OH solution (28–30%) and methanol (1:1). The resulting mixture was stirred at room temperature for 24 h. After 24 h, reaction progress was monitored by TLC. Upon completion of the reaction, the solvent was evaporated under reduced pressure, and the resulting crude (**2**) was filtered off and washed with ethyl acetate. Yield 66%. ^1^H NMR (D_2_O), δ, ppm: 8.38 (s, 1H, H2), 7.95 (d, 1H, J = 6.69 Hz, 1H, H4), 6.53 (d, 1H, J = 9.44 Hz, H5), 3.62 (s, 3H, OMe). ^13^C NMR (CDCl_3_), δ, ppm: 167.17 (CONH_2_), 163.69 (C2), 142.03 (C6), 138.61 (C4), 117.89 (C3), 113.52 (C5), 37.27 (Me). HRMS calcd for C_7_H_9_N_2_O_2_ [M + H]^+^ 153.0664 found 153.0663.

#### 4.1.4. Synthesis of *N*-methyl-6-pyridone 3-carboxamide (**2**)

The synthesis of 1-*N*-methyl-2-pyridone-3-carboxamide was conducted according to Scheme 4.

*N*-Methylnicotinamide (**7**) (0.5 g, 1.89 mmol) was dissolved in 10 mL NaOH (1M, aq.) and then commercially available K_3_[Fe(CN)_6_] (1.87 g, 5.68 mmol), dissolved in 2 mL deionized water was added. The resulting mixture was stirred at rt for 1 h. After 1 h, 25 mL MeOH was added to precipitate K_4_[Fe(CN)_6_] and then the resulting mixture was filtered off. The filtrate was concentrated on rotavap and the crude was purified by using hexane, ethyl acetate and methanol as an eluent to afford 1-methyl-2-oxo-pyridine-3-carboxamide (**5**) (15%) and 1-methyl-6-oxo-pyridine-3-carboxamide (**2**) (25%). ^1^H NMR (DMSO-*d*_6_), δ, ppm: 9.06 (s, 1H, NH), 8.27 (dd, J = 2.2 Hz & 2.16 Hz, 1H, H6), 8.01 (dd, J = 2.16 & 2.16 Hz, 1H, H4), 7.54 (s, 1H, NH), 6.44 (t, J = 6.88 Hz, 1H, H5), 3.08 (s, 3H, Me). ^13^C NMR (DMSO-*d*_6_), δ, ppm: 165.08 (CONH_2_), 162.22 (CO), 144.43 (C6), 143.70 (C4), 120.45 (C3), 106.10 (C5), 38.16 (CH_3_) HRMS calcd for C_7_H_9_N_2_O_2_ [M + H]^+^ 153.0664 found 153.0648 [29].

#### 4.1.5. Synthesis of the Ribosylated Pyridone Carboxamides

##### Synthesis of 4-pyridone-3-carboxamide riboside (**3**)

The synthesis of 4-pyridone-3-carboxamide riboside was conducted according to Scheme 5.

*Synthesis of 4-pyridone-3-carboxamide* (**16**) *(Step-1)*: A sealed tube equipped with a Teflon-coated magnetic stirring bar was charged with methyl 4-pyridone-3-carboxylate (**11**) (2 g, 13.0 mmol) and 50 mL of NH_4_OH methanolic solution (28–30%). The resulting mixture was stirred at room temperature for 15 h. Upon completion of the reaction, the solvent was evaporated at reduced pressure, and the residue was triturated with water. The solid (**16**) was filtered off and washed with water. Yield 66%. ^1^H NMR (MeOD), δ, ppm: 8.54 (s, 1H, H2), 7.79 (d, J = 5.56 Hz, 1H, H6), 6.56 (d, J = 6.53 Hz, 1H, H5). ^13^C NMR (CDCl_3_), δ, ppm: 179.04 (C4), 168.48 (CONH2), 142.95 (C2), 139.32 (C6), 119.45 (C5), 117.69 (C3); HRMS calcd for C_6_H_7_N_2_O_2_ [M + H]^+^ 139.0508 found 139.0495 [25].

*Synthesis of 1-(2′,3′,5′-tri-*O*-acetyl-β-d-ribofuranosyl)-4-pyridone-3-carboxamide* (**18**) *(Step-2)*: Silylation of 4-pyridone-3-carboxamide (**17**): 4-pyridone-3-carboxamide (**16**) (0.5 g, 3.62 mmol): Hexamethyldisilazane (10 mL) and trimethylchlorosilane (1.5 mL, 1.30 g, 12 mmol) were added sequentially in a 50 mL round bottom flask under nitrogen. The resulting mixture was stirred at 120 °C for 2 h. Upon completion of the reaction, the solution was cooled to room temperature and concentrated under reduced pressure. The residue was co-evaporated with anhydrous toluene (2 to 3 times) to afford the mixture of mono and bis-silylated 4-pyridone-3-carboxamide (**17**), which was used directly for the next step.

Vorbrüggen glycosylation *(Step-3)*: The crude mono and bis-silylated 4-pyridone-3-carboxamide (**17**) mixture was dissolved in 6 mL of anhydrous 1,2- dichloroethane. Then, a solution of 1,2,3,5-tetra-*O*-acetyl-β-d-ribofuranoside (0.6 g, 2 mmol) in 2 mL of 1,2-dichloroethane was added, followed by the addition of trimethylsilyl triflate (0.4 mL, 2.3 mmol). The resulting mixture was stirred at 40–45 °C for overnight. The reaction was monitored by ^1^H NMR analysis of the crude mixture. After completion of the reaction, the resulting solution was cooled down and poured into a mixture of ice-cooled saturated aq. NaHCO_3_ solution. The mixture was extracted with DCM (3 × 10 mL). The organic phases were collected, washed with a saturated NaCl solution, and dried over anhydrous Na_2_SO_4_. Then, the filtrate was concentrated under reduced pressure and the residue was purified by flash column chromatography using DCM: Acetone (7:3) as an eluent to afford the pure product (**18**) (72%). ^1^H NMR (MeOD), δ, ppm: 8.75 (d, J = 2.4 Hz, 1H, H2), 7.87 (dd, J = 2.4 Hz, 2.4 Hz, 1H, H6), 6.51 (d, J = 7.6 Hz, 1H, H5), 5.80 (d, J = 5.6 Hz, 1H, H1′), 5.38–5.25 (m, 2H, H2′ and H3′), 4.45 (q, J = 3.07 Hz,1H, H4′), 4.36 and 4.27 (AB part of ABX system, 2H, JAB = 12.56 Hz, JAX = 3.12 Hz, JBX = 2.76 Hz, H5′), 2.08 (s, 3H, Ac), 2.04 (s, 3H, Ac), 1.98 (s, 3H, Ac). ^13^C NMR (MeOD), δ, ppm: 178.69 (C4), 170.73 (CO), 169.88 (CO), 169.69 (CO), 166.32 (CONH2), 141.78 (C2), 138.23 (C6), 119.91 (C5), 119.04 (C3), 94.59 (C1′), 81.48 (C4′), 74.48 (C2′), 70.43 (C3′), 62.83 (C5′), 19.28 (Me), 18.96 (Me), 18.73 (Me); HRMS calcd for C_17_H_21_N_2_O_9_ [M + H]^+^ 397.1249 found 397.1229.

*Synthesis of 4-Pyridone-3-carboxamide-1-β-d-ribofuranoside* (**3**) *(Step-4)*: Method A: A mixture of compound (**18**) (0.078 g, 0.196 mmol), 1 mL NH_4_OH (27–30%) and cat. K_2_CO_3_ in methanol (10 mL) was stirred at rt overnight, and the reaction progress was monitored by ^1^H NMR analysis of the crude reaction. Upon completion of the reaction, the reaction mixture was filtered and evaporated in vacuo to afford the desired product 3 as semisolid. Yield 50%.

Method B: A sealed tube was charged with a mixture of compound (**18**) (0.408 g, 1.02 mmol), 2 mL 1,4-dioxane (saturated with 0.5N NH_3_), and 15 mL methanol. The resulting mixture was stirred at rt overnight, and the reaction progress was monitored by NMR analysis of the crude mixture. After overnight stirring at rt, the ^1^H NMR of the crude showed complete removal of the acetates. The resulting mixture was concentrated under reduced pressure to afford the desired product 3. Yield: 62%.

Method C: A PTFE jar was charged with pure compound (**18**) (0.2 g, 0.50 mmol), K_2_CO_3_ (6.91 mg, 0.1 mmol) and 20 microliter MeOH and was vibrated at a rate of 1800 rpm (30 Hz) at room temperature for 30 min. The reaction progress was monitored by ^1^H NMR analysis. Yield: 85%. ^1^H NMR (D_2_O), δ, ppm: 8.67 (d, J = 2.24 Hz, 1H, H2), 7.94 (dd, J = 2.24 Hz, 2.24 Hz, 1H, H6), 6.65 (d, J = 7.6 Hz, 1H, H5), 5.55 (d, J = 5.5 Hz, 1H, H1′), 4.23 (t, 1H, J = 5.28 Hz, H2′), 4.19–4.13 (m, 2H, H4′ and H3′), 3.81 and 3.73 (AB part of ABX system, 2H, JAB = 12.7 Hz, JAX = 3.04 Hz, JBX = 4.0 Hz, H5′). ^13^C NMR (D_2_O), δ, ppm: 179.13 (C4), 167.75 (CONH_2_), 142.72 (C2), 138.73 (C6), 120.35 (C5), 118.25 (C3), 96.96 (C1′), 86.03 (C4′), 75.61 (C2′), 70.02 (C3′), 60.89 (C5′); HRMS calcd for C_11_H_15_N_2_O_6_ [M + H]^+^ 271.0930 found 271.0919.

##### Synthesis of 6-pyridone 3-carboxamide riboside (**4**)

The synthesis of 6-pyridone 3-carboxamide riboside was conducted according to Scheme 6.

*Synthesis of 6-pyridone 3-carboxamide (6PY)* (**20**) *(Step-1)*: To an oven-dried 50-mL round bottom flask equipped with a magnetic stir bar, 6-hydroxynicotinic acid (**13**) (5 g, 3.59 mmol), HOBt (0.540 g, 4.0 mmol), DCC (0.928 g, 4.5 mmol), and anhydrous DMF (10 mL) were added. The RBF was placed under nitrogen sealed with a septum. The reaction mixture was stirred at rt for 2 h under nitrogen. The reaction progress was monitored by ^1^H NMR of the crude reaction mixture. Upon completion of the reaction, the solid residue (**19**) was filtered off and washed with fresh DMF (10 mL). The organic layer was concentrated under reduced pressure, and the residue was dissolved in 10 mL NH_4_OH (28–30%) and stirred at rt for 1 h. The reaction progress was monitored by ^1^H NMR of the crude reaction mixture. After completion of the reaction, the solvent was evaporated under high vacuum to afford the 6-pyridone-3-carboxamide (**20**) (6PY) as a brown solid. Yield 70%. ^1^H NMR (DMSO, d^6^), δ, ppm: 11.92 (s, 1H, NH), 8.00 (s, 1H, H2), 7.85 (d, J = 9.6 Hz, 1H, H4), 7.72 (s, 1H, NH), 7.18 (s, 1H, NH), 6.32 (d, J = 9.56 Hz, 1H, H5). HRMS calcd for C_12_H_16_NO_7_ [M + H]^+^ 139.0508 found 139.0499 [31].

*Silylation of 6-pyridone-3-carboxamide* (**21**) *(Step-2)*: 6PY (**20**) (0.3 g, 2.17 mmol) and *N,O*-Bis (trimethylsilyl) trifluoroacetamide (2.3 mL, 2.23 g, 8.69 mmol) were dissolved in anhydrous acetonitrile (5 mL) at rt, and the reaction mixture was stirred at rt for 24 h under nitrogen. After 24 h, the reaction mixture was concentrated under reduced pressure, and the crude product was used directly in the next step.

*Synthesis of 1-(2′,3′,5′-tri-*O*-acetyl-β-d-ribofuranosyl)-6-pyridone-3-carboxamide* (**22**) *(Step-3)*: To a mixture of pre-silylated 6-pyridone-3-carboxamide (**21**) (0.5 g, 1.6 mmol) and 1, 2, 3, 5-tetra-*O*-acetyl-β-ribofuranoside (0.767 g, 2.4 mmol) in anhydrous 1,2-dichloroethane (20 mL) was added SnCl_4_ (0.754 mL, 1.67 g, 6.4 mmol) and stirred at rt for 16 h under nitrogen gas environment. Once the reaction was complete, the reaction mixture was poured into water and extracted with CHCl_3_ (10 mL × 3), washed with saturated NaHCO_3_ and dried over anhydrous Na_2_SO_4_. The organic layer was concentrated, and the oily product was purified by flash column chromatography. Yield 78%. ^1^H NMR (CDCl_3_), δ, ppm: 8.30 (br.s., 1H, H6), 7.69 (d, J = 9.04 Hz, 1H, H4), 6.45 (d, J = 9.44 Hz, 1H, H3), 6.26 (br.s, 1H, H1′), 5.31 (t, J = 4.58 Hz, 1H, H2′), 5.20 (t, J = 5.20 Hz, 1H, H3′), 4.56–4.51 (m, 1H, H4′), 4.38 (br. s, 1H, H5′ ABX system), 4.26 (d, J = 12.36 Hz, H5′ ABX system), 2.13 (s, 3H, Ac), 2.05 (s, 3H, Ac), 2.04 (s, 3H, Ac). ^13^C NMR (CDCl_3_), δ, ppm: 171.43 (C2), 169.60 (CO), 169.36 (CO), 165.73 (CO), 161.80 (CONH_2_), 141.66 (C6), 135.66 (C4), 121.59 (C3), 119.89 (C5), 87.96 (C1′), 80.10 (C4′), 74.13 (C2′), 69.74 (C3′), 62.74 (C5′), 20.88 (Me), 20.48 (Me), 20.45 (Me). MS (ES): *m/z* 397.63 [M^+^ + H] and 419.56 [M^+^ + Na].

*Synthesis of 6-pyridone-3-carboxamide-1-β-d-ribofuranoside* (**4**) *(Step-4)*: The triacetylated precursor (**22**) (0.360 g, 0.9 mmol) was dissolved in 2 mL ordinary methanol and stirred at rt for 15 min. After 15 min, 2 mL NH_4_OH (28–30%) was added, and the resulting mixture was stirred at rt for 4 h. The reaction progress was monitored by ^1^H NMR analysis of the crude reaction mixture. Upon completion of the reaction, the solvent was evaporated under reduced pressure, and the crude was kept at rt overnight before treating with acetone for 24 h. The solid was then filtered off and washed with fresh acetone to afford 6-PYR (**4**) as a white solid. Yield 72%. ^1^H NMR (D_2_O), δ, ppm: 8.60 (s, 1H, H6), 7.84 (d, J = 9.2 Hz, 1H, H4), 6.54 (d, J = 9.44 Hz, 1H, H5), 6.01 (s, 1H, H1′), 4.19–4.13 (m, H2′, H3′ & H4′), 3.97 (d, J = 12.92 Hz, 1H, H5′, ABX system), 3.80 (d, J = 12.88 Hz, 1H, H5′, ABX system). ^13^C NMR (CDCl_3_), δ, ppm: 169.12 (CONH_2_), 163.80 (C2), 139.30 (C6), 139.02 (C4), 118.80 (C3), 114.30 (C5), 90.75 (C1′), 83.56 (C4′), 74.87 (C2′), 68.24 (C3′), 59.83 (C5′), HRMS calcd for C_11_H_15_N_2_O_6_ [M + H]^+^ 271.0930 found 271.0938.

##### Synthesis of *O*-methyl-4-pyridone-3-carboxylate-1-β-d-ribofuranoside (**26**)

The synthesis of *O*-methyl-4-pyridone-3-carboxylate-1-β-d-ribofuranoside was conducted according to Scheme 7.

*Silylated 4-pyridone-3-carboxylic methyl ester* (**23**) *(Step-1): O-*Methyl-4-pyridone-3-carboxylic ester (**11**) (3.0 g, 1.98 mmol) was dissolved in 50 mL of hexamethyl-disilazane (HMDS) in an RBF under nitrogen, 5 mL of trimethylsilyl chloride (TMSCl) (0.494 mL, 0.423 g, 3.9 mmol) was then added. The mixture was refluxed under nitrogen for 3 h at 120–130 °C. The excess reagents were evaporated and co-evaporated with toluene (10 mL). The product was a dark yellow oily residue. Yield 90%. ^1^H NMR (400 MHz, CDCl_3_, δ, ppm): 8.92 (s, 1H, H2), 8.44 (d, J = 5.6 Hz, 1H, H6), 6.71 (d, J = 5.6 Hz, 1H, H5), 3.83 (s, 3H, OMe), 0.27 (s, 9H, Si(CH_3_)_3_).

*Synthesis of 1-(2′,3′,5′-Tri-*O*-acetyl-β-d-ribofuranosyl)-4-pyridone-3-carboxylic methyl ester* (**24**) *(Step-2)*: Silylated 4-pyridone-3-carboxylic methyl ester (**23**) was dissolved in 25 mL of 1,2-dichloroethane (DCE) under nitrogen. 1,2,3,5-tetra-*O*-acetyl-δ-d-ribofuranoside (6.30 g, 1.98 mmol) was dissolved in DCE (25 mL) and then added to the ester solution. An additional 50 mL of DCE and 4.1 mL of trimethylsilyl triflate (TMSOTf) (2.28 mmol) were added to the mixture. The resulting mixture was stirred at 40 °C for 5 h under nitrogen. Upon completion of the reaction, the reaction mixture was cooled to 0 °C and poured into iced water. The reaction flask was rinsed with DCM (50 mL) and added to the quenched reaction solution. The mixture was neutralized with 35 mL of a saturated aq NaHCO_3_ solution. The resulting phases separated, and the organic layer was washed with saturated NaCl solution (50 mL). Once separated, the organic phase was dried over anhydrous sodium sulfate, filtered, and evaporated. The crude product was purified by flash column chromatography using n-hexane and ethyl acetate as an eluent system to afford the desired product (**24**) as a light brown crystalline solid (6.42 g, 75%). ^1^H NMR (400 MHz, CDCl_3_, δ, ppm): 8.40 (d, J = 2.5 Hz, 1H, H-2), 7.45 (dd, J = 7.9 and 2.5 Hz, 1H, H-6), 6.52 (d, J = 7.9 Hz, 1H, H-5), 5.49 (d, J = 5.9 Hz, 1H, H-1′), 5.30 (dd, J = 5.6 and 3.6 Hz, 1H, H-3′), 5.21 (dd, J = 5.9 and 5.6 Hz, 1H, H-2′), 4.33–4.46 (m, 3H, H-5′A, H-5′B, H-4′), 3.88 (s, 3H, O-CH_3_), 2.19 (s, 3H, Ac), 2.14 (s, 3H, Ac), 2.10 (s, 3H, Ac); ^13^C NMR (100 MHz, CDCl_3_, δ, ppm): 175.7 (COO), 170.1 (CO), 169.4 (CO), 169.3 (CO), 165.6 (C-4), 142.4 (C-2), 135.1 (C-6), 122.9 (C-5), 119.4 (C-3), 94.22 (C-1′), 81.34 (C-4′), 74.29 (C-2′), 70.21 (C-3′), 62.76 (C-5′), 52.40 (–OCH_3_), 20.47 (CH_3_), 20.31 (CH_3_); HRMS calcd for C_18_H_22_NO_10_ [M + H]^+^ 412.1243 found 412.1243.

*Synthesis of O-methyl-4-pyridone-3-carboxylate ribonucleoside* (**25**) *(Step-3)*: A mixture of 1-(2′,3′,5′-tri-*O*-acetyl-*β*-d-ribofuranosyl)-4-pyridone-3-carboxylic methyl ester (**24**) (3.20 g, 7.79 mmol), K_2_CO_3_ (0.103 g, 0.78 mmol), and MeOH (1.2 mL, 29.6 mmol) was ball-milled for 30 min at 30 Hz. Following removal of methanol, a brown solid with a taffy-like consistency was obtained (1.81 g, 95%). ^1^H NMR (400 MHz, MeOD, δ, ppm): 3.76 (dd, J = 12.1 and 2.7 Hz, 1H, H-5′A), 3.83 (s, 3H, -CH_3_), 3.85 (dd, J = 2.7 Hz and J = x, 1H, H-5′B), 4.15 (dd, J = 6.3 and 2.6 Hz, 1H, H-4′), 4.17–4.22 (m, 2H, H-2′, H-3′), 5.49 (d, J = 5.1 Hz, 1H, H-1′), 6.53 (d, J = 7.7 Hz, 1H, H-5), 8.09 (dd, J = 7.7 and 2.3 Hz, 1H, H-6), 8.78 (d, J = 2.3 Hz, 1H, H-2); ^13^C NMR (100 MHz, MeOD, δ, ppm): 50.89 (–OCH_3_), 61.19 (C-5′), 70.94 (C-3′), 76.33 (C-2′), 87.11 (C-4′), 97.57 (C-1′), 117.7 (C-3), 120.6 (C-5), 137.7 (C-6), 143.8 (C-2), 164.8 (C-4), 177.4 (COO); HRMS calcd for C_12_H_16_NO_7_ [M + H]^+^ 286.0926 found 286.0929.

*Synthesis of 4-pyridone-3-carboxylic acid ribonucleoside* (**26**) *(Step-4)*: A mixture of 4-pyridone-3-carboxylic ester riboside (**25**) (2.43 g, 8.85 mmol) was dissolved in a 1M NaOH aqueous solution (10 mL) and stirred for 1 h. Upon completion of the reaction, the resulting mixture was neutralized with 1 M HCl solution (5 mL) and evaporated on high vacuum (80%). ^1^H NMR (400 MHz, D_2_O, δ, ppm): 8.21 (d, J = 2.4 Hz, 1H, H-2), 7.89 (dd, J = 7.6 and 2.4 Hz, 1H, H-6), 6.52 (d, J = 7.6 Hz, 1H, H-5), 5.51 (d, J = 5.6 Hz, 1H, H-1′), 4.26 (dd, J = 5.6 and 5.3 Hz, 1H, H-2′), 4.20 (dd, J = 5.3 and 3.6 Hz, 1H, H-3′), 4.15 (dd, J = 8.3 and 3.6 Hz, 1H, H-4′), 3.82 (dd, J = 12.8 and 4.5 Hz, 1H, H-5′B), 3.74 (dd, J = 12.8 and 4.5 Hz, 1H, H-5′A); ^13^C NMR (100 MHz, D_2_O, δ, ppm): 60.97 (C-5′), 70.03 (C-3′), 5.36 (C-2′), 85.81 (C-4′), 96.67 (C-1′), 118.9 (C-5), 127.3 (C-6), 138.4 (C-2), 138.8 (C-3), 172.0 (C-4), 178.1 (COO). HRMS calcd for C_11_H_14_NO_7_ [M + H]^+^ 272.0770 found 272.0768.

##### Synthesis of 2-pyridone-3-carboxamide Riboside (**6**)

The synthesis of 2-pyridone-3-carboxamide riboside was conducted according to Scheme 8.

*Synthesis of 2-pyridone-3-carboxamide* (**29**) *(step-1)*: 2-Hydroxypyridine-3-carbonyl chloride (intermediate) was synthesized via the chlorination of 2-hydroxy nicotinic acid (**27**) (1.0 g, 7.18 mmol) by using SOCl_2_ (10 mL). The reagents were added to a 50-mL one-neck round-bottom flask equipped with a condenser. The solution was stirred at reflux (90 °C) for 1–2 h. After that time, the resulting mixture with solid residue was concentrated on a rotary evaporator. A yellowish-green solid was obtained, which was dissolved immediately in 15 mL anhydrous methanol and stirred at rt for 1 h for their conversion to methyl 2-hydroxypyridine-3-carboxylate (**28**). After completion of the reaction, the solvent was removed, and the crude 2-hydroxypyridine-3-carboxylate (**28**) was dissolved in 10 mL NH_3_ saturated methanol, and then the round bottom flask was sealed with a septum. The resulting mixture was stirred at rt for the next 60 h. After 60 h, the precipitated white solid was filtered off and washed with cold methanol (2–3 times) and dried to yield 70% of 2-oxo-1H-pyridine-3-carboxamide (**29**). ^1^H NMR (DMSO-*d_6_*), δ, ppm: 12.3 (s, 1H, NH), 9.05 (s, 1H, NH), 8.29 (d, J = 5.28 Hz, 1H, H6), 7.66 (d, J = 4.32 Hz, 1H, H4), 7.51 (s, 1H, NH), 6.41 (t, J = 6.66 Hz, 1H, H5). ^13^C NMR (DMSO-*d_6_*), δ, ppm: 165.09 (CONH_2_), 162.71 (CO), 144.69 (C4), 140.04 (C6), 121.27 (C3), 160.44 (C5); HRMS calcd for C_6_H_6_N_2_O_2_ [M + H]^+^ 139.0507 found 139.0493.

*Silylation of 2-pyridone-3-carboxamide* (**30**) *(Step-2)*: 2-pyridone-3-carboxamide (**29**) (426 mg, 3.08 mmol), hexamethyldisilazane (10 mL) and (NH_4_)_2_SO_4_ (cat. amount) were added sequentially to a 50-mL round bottom flask under nitrogen. The resulting mixture was stirred at 135 °C for 2 h. Upon completion of the reaction, the resulting solution was cooled to room temperature and then concentrated under vacuum. The residue was co-evaporated with anhydrous toluene (2 to 3 times) to afford the mixture of mono and bis-silylated 2-pyridone-3-carboxamide (**30**), which was used directly for the next step.

*Vorbrüggen glycosylation (Step-3)*: The crude mono and bis-silylated 2-pyridone-3-carboxamide (**30**) was dissolved in 10 mL of anhydrous CH_3_CN. Then, a solution of 1,2,3,5-tetra-*O*-acetyl-β-d-ribofuranoside (1.017 g, 3.20 mmol) in 2 mL of anhydrous acetonitrile was added, followed by the addition of a solution trimethylsilyl triflate (1.67 mL, 9.24 mmol). The resulting mixture was stirred at rt for 24 h, and the reaction progress was monitored by NMR analysis of crude mixture. After completion of the reaction, the resulting solution was concentrated under vacuum, and the residue was purified by flash column chromatography using n-hexane: ethyl acetate (1:1.5) as an eluent to afford pure products (**31**) (81%).

*Deacetylation of compound***31***(Step 4)*: Compound **31** (1.01 g, 2.52 mmol) was dissolved in 10 mL methanol (saturated with ammonia) and stirred at rt for 48 h under sealed condition. The reaction progress was monitored by the NMR analysis of crude reaction mixture. Upon completion of the reaction, solvent was evaporated on high vacuum and the crude product was stirred with acetone and filtered off and washed with fresh acetone to afford 2-PYR (**6**) as white solid. The obtained product was enough pure. Yield 44%. ^1^H NMR (MeOD), δ, ppm: 8.46 (dd, J = 5.4 Hz & 7.04 Hz, 2H, H4 & H6), 6.55 (t, J = 6.96 Hz, 1H, H5), 6.14 (s, 1H, H1′), 4.12 (bs, 3H, H2′, H3′ & H4′), 3.97 (d, J = 12.9 Hz, 1H, H_a_5′), 3.80 (d, J = 11.56 Hz, 1H, H_b_5′). ^13^C NMR (MeOD), δ, ppm: 166.62 (CONH_2_), 161.79 (CO), 144.02 (C6), 138.21 (C4), 119.69 (C3), 105.90 (C5), 91.11 (C1′), 84.38 (C4′), 75.71 (C2′), 68.58 (C3′), 59.88 (C5′), HRMS calcd for C_11_H_15_N_2_O_6_ [M + H]^+^ 271.0930 found 271.0909 [32].

##### Synthesis of 2PYR and 6PYR via Fenton Chemistry

Nicotinamide riboside chloride (0.20 g, 0.78 mmol) was dissolved in aqueous NaOH (1 M) at 0 °C and rapidly added to a solution of K_3_[Fe(CN)_6_] (0.773 g, 2.35 mmol) in 10 mL H_2_O. The resulting mixture was stirred at 0 °C for 1 h and then warmed to rt. After 1 h stirring at rt, 50 mL of MeOH was added. The mixture was stirred at rt for 2 h. A thick yellowish precipitate formed during stirring and was filtered off and washed with MeOH (2–3 times). NMR of the crude reaction mixture showed the formation of 2PYR and 6PYR but not that of 4PYR.

### 4.2. Enzymatic Conversions

#### 4.2.1. Synthesis of 4PYR via NQO2 Catalyzed Hyper-Oxidation of the Reduced Form of Nicotinamide Riboside (NR), Abbreviated NRH

To prepare the sample, 1 mM NR in 450 µL buffer (50 mM potassium phosphate buffer, 125 mM NaCl, 5 µM FAD, 1.0 mg/mL BSA) was reacted with 20 µL of the NQO2 enzyme (Sigma-Aldrich, Q0380) and allowed to incubate overnight at room temperature. NRH was also incubated overnight with the same buffer composition for comparison. An Agilent 1200 series HPLC was used for the purification and isolation of compounds. Five microliters of each sample were injected onto an Agilent C18 reversed-phase column (2.1 × 150 mm) equipped with a C18 guard column using an autosampler. Solvent A consisted of H_2_O with 2.0% acetonitrile (ACN) and 0.1% trifluoroacetic acid (TFA), and solvent B consisted of ACN with 2.0% H_2_O and 0.1% TFA. A flow rate of 100 µL per minute was used for the entirety of the run. The solvent was held at 5.0% B for the first 5 min, from 5 to 20 min a gradient from 5.0 to 80.0% B was used, then solvents were held at 80% B for 5 min and returned to 5.0% B for the remainder of the run. Two injections were performed for each sample, one scanning at 260 nm (for the UV detection of NR and 4PYR) and the other injection scanning at 340 nm (for the UV detection of NRH). Two fractions were collected based on the retention time of previously analyzed standards. The first from 5 to 8 min for the collection of NRH and 4PYR, and the second fraction from 19 to 26 min for the collection of NR. The collected fractions were dried using a speedvac concentrator (without heat applied). Samples were dissolved into 50 µL of 10 mM ammonium bicarbonate (ABC) for analysis by mass spectrometry.

#### 4.2.2. LC-MS/MS Analyses

Liquid chromatography-mass spectrometry analyses were performed on the individual HPLC fractions that were previously prepared. Each fraction was analyzed separately. An Agilent 1200 Series HPLC coupled to a Thermo LTQ Orbitrap XL mass spectrometer was used for sample analysis. An autosampler was used to inject the samples onto an Agilent Zorbax 300SB-C18 reversed-phase column (2.1 × 150 mm, 5-micron) using a 4.0 µL injection volume for each. Solvent A consisted of 10 mM ABC in H_2_O, and Solvent B consisted of neat ACN. A flow rate of 50 µL per minute was used for the first 5 min of the run at 5.0% B solvent, followed by a gradient from 5.0–80.0% B from 5 to 15 min along with an increased flow rate of 100 µL per minute. The solvent was held at 80.0% B from 15 to 25 min and returned to 5.0% B for the remainder of the run. A HESI (heated electrospray ionization) source was used with positive polarity, a capillary temperature of 200 °C, the source voltage of 3.0 kV, tube lens voltage of 110, and a sheath gas flow rate of 8.0. One full scan from 80–800 *m/z* was performed at 15,000 resolution, followed by targeted MS2 scans of NR (255.1 *m/z*), NRH (257.1 *m/z*), and 4PYR (271.1 *m/z*) at 7500 resolution with an isolation width of 1.0 *m/z*. Normalized CID collision energy was set to 35. All scans were performed in the FTMS. The total run time was 30 min. A blank was run in-between each sample to minimize and monitor for carryover.

### 4.3. Cell-Based Assays

#### 4.3.1. Cell Culture

Human embryonic kidney containing the SV40 T-antigen (HEK293T) and the human hepatoma cell line (HepG3) were purchased from ATCC (Manassas, VA, USA). The cells were grown at 37 °C in a 5% CO_2_ incubator in Dulbecco’s modified Eagle’s medium (DMEM, Hyclone, Logan, UT, USA; 4.5 g/L glucose and L-glutamine) supplemented with 10% fetal bovine serum (FBS, Atlanta Biologicals, Flowery Branch, GA, USA), and 1% sodium pyruvate (Gibco, Carlsbad, CA, USA). Cells were routinely tested for mycoplasma contamination using the Lonza MycoAlert kit (Walkersville, MD, USA) and found to be free of mycoplasma contamination.

#### 4.3.2. Cytotoxicity Studies

Cell viability was determined by CellTiter-Fluor^TM^ Cell Viability assays (Promega Corporation, Madison, WI, USA). HEK293T and HepG3 cells were seeded at a density of 5000 cells/well and 10,000 cells/well respectively in a 96-well clear bottom black plate and incubated overnight (ON) at 37 °C in a 5% CO_2_ incubator. For HEK293T cells, a poly d-lysine-coated black plate was used to prevent washing off the cells during medium replacement. For 24 h exposures, the cells were exposed to 100 µM concentrations alone or increasing doses (25, 50, 75, 100, and 150 µM) of pyridone derivatives (i.e., 2-PYR, 4-PYR, and 6-PYR) for 24 h, then medium containing NRH was replaced with fresh medium, and cells were further incubated for another 48 h (total 72 h) at 37 °C in a 5% CO_2_ incubator. For continuous exposures, the cells were exposed to 100 µM concentrations alone or increasing doses (25, 50, 75, 100, and 150 µM) of pyridone derivatives (i.e., 2-PYR, 4-PYR, and 6-PYR) for 72 h. The 2-PYR, 4-PYR, and 6-PYR were dissolved in Dulbecco’s phosphate-buffered saline (PBS, Hyclone, Logan, UT, USA) at 10 mM and then diluted to the final working concentrations in the cell growth medium. At the end of the 72-h incubation period, 100 µL of 2X CellTiter-Fluor^TM^ Viability reagent was added in each well, mixed briefly, and then the plates were incubated for another 30 min at 37 °C in a 5% CO_2_ incubator. Fluorescence intensity was measured in a microplate reader (Infinite^®^ M1000 PRO, TECAN, Männedorf, Switzerland) with a fluorometer at excitation/emission (Ex/Em) of 380/505 nm. Cells were plated in triplicate for each chemical concentration and exposure durations with at least three biological repeats performed. Fluorescent values are expressed as the number of cells in drug-treated wells relative to cells in vehicle-treated, control wells (%viability) ± standard error of the mean (SEM).

#### 4.3.3. Clonogenic Assays

A clonogenic assay was used to determine the cell growth and colony formation after exposure. HepG3 cells were plated at a density of 10,000 cells/well in each well of a 12-well plate and incubated ON at 37 °C in a 5% CO_2_ incubator. The following day, cells were exposed to 25, 50, and 100 µM 4-PYR continuously for six days. At the end of the incubation period, plates were placed on ice, washed twice with ice-cold 1 X PBS before fixing the cells with ice-cold methanol for 10 min. Following the fixation step, cells were incubated with 0.5% crystal violet solution (made in 25% methanol) for 10 min at room temperature (RT, ~23 °C). The plates were rinsed with double-distilled water to remove the excess crystal violet stain and allowed to dry ON at RT. Stained plates were imaged using ChemiDoc^TM^ MP Imaging System (Bio-Rad, Hercules, CA, USA). For analysis, the area fraction of the crystal violet-stained cells was generated from each image using NIS-Elements software. The percentage of the average area fraction of crystal violet staining per well was calculated. Each treatment was performed in technical triplicates and averaged over three biological replicates with final cell density percentage reported relative to control ± SEM.

#### 4.3.4. Immunoblotting

The cell death mechanism of 4-PYR induced cytotoxicity was assessed by immunoblotting. Briefly, HepG3 cells were plated in 65-mm dishes at a density of 2 × 10^6^ cells per dish and incubated ON at 37 °C in a 5% CO_2_ incubator. The cells were treated with 100 µM 4-PYR for 48, 72, and 96 h. Cell pellets were collected at the end of the desired exposure period and stored at −80 °C. The cell pellets were thawed on ice and resuspended in a lysis buffer of 25 mM β-glycerolphosphate, 50 mM Tris-HCl, pH 7.5, 150 mM NaCl, 0.2% Triton X-100, and 0.3% NP-40 supplemented with 1X Halt protease and phosphatase inhibitor (Pierce, Waltham, MA, USA). Resuspended cells were incubated for 30 min on ice. Lysates were centrifuged at 12,000 rpm for 15 min at 4 °C, and the supernatant fraction containing protein was retained. Protein concentrations were quantified using Bradford Quick Start protein assay (Bio-Rad, Hercules, CA, USA). About 30 µg of each protein sample was separated on a 4–15% SDS-PAGE gel and transferred onto a nitrocellulose membrane (Bio-Rad). Membranes were blocked in 5% skim milk in Tris-buffered saline (TBS, VWR Life Sciences) containing 0.1% Tween 20 (TBS-T) and incubated with the following antibodies: LC3B (1:1000, PA1-46286) from ThermoFisher (Rockford, IL, USA); Beclin-1 (1:1000) and phosphor-mTOR (1:1000, Ser2448, D92C) from Cell Signaling Technology, Inc. (Danvers, MA, USA); and α-tubulin (1:5000, T9026) from Millipore Sigma (St. Louis, MO, USA).

## 5. Conclusions

Here, we have synthesized and systematically characterized all pyridones-derived from nicotinamide known to have some physiological relevance and sought the biochemical origin of the PYR series that had remained unknown. Indeed, we report that 4PYR can be generated from NRH by the FAD-dependent NQO2 enzyme and provide evidence that 2PYR and 6PYR can be generated from NR via Fenton chemistry, thus further supporting the hypothesis that reductive stress, as well as oxidative stress, promotes the formation of ribosylated pyridones.

## Figures and Tables

**Table 1:** Tables of ^1^H NMR chemical shift for the non-substituted pyridones.

Compound (Solvent)	Chemical Shift (Multiplicity, J Values in Hz)					
	NH	H2	H3	H4	H5	H6
2PY (DMSO-*d_6_*)	12.3 (s), 9.05 (s), 7.51 (s)			7.66 (d, J = 4.32 Hz)	6.41 (t, J = 6.66 Hz)	8.29 (d, J = 5.28 Hz)
2PY (DMSO-*d_6_*) [25]	11.80 (s), 9.02 (s), 7.57 (s)			7.69 (q, 1H)	6.44 (t)	8.31 (q)
4PY (CD_3_OD)		8.54 (s)			6.56 (d, J = 6.53 Hz)	7.79 (d, J = 5.56 Hz)
4PY (CD_3_OD) [25]		8.56 (d, J = 1.7 Hz)			6.58 (d, J = 7.2 Hz)	7.80 (dd, J = 7.2, 1.7 Hz)
6PY (DMSO-*d_6_*)	11.92(s), 7.72 (s) 7.18 (s)	8.00 (s)		7.85 (d, J = 9.6 Hz)	6.32 (d, J = 9.56 Hz)	
6PY (DMSO-*d_6_*) [31]	11.84 (s), 7.71 (s), 7.18 (s)	7.99 (d, J = 2.6 Hz)		7.82–7.87 (dd, J = 2.6 & 9.5 Hz)	6.32 (d, J = 9.5 Hz)	

**Table 2:** Tables of ^1^H NMR chemical shift for the methylated pyridones.

Compound (Solvent)	Chemical Shift (Multiplicity, J Values in Hz)						
	NH	H2	H3	H4	H5	H6	CH_3_
*N*-Me-2PY (DMSO-*d_6_*)	9.06 (s) 7.54 (s)			8.01 (dd, J = 2.16 & 2.16 Hz)	6.44 (t, J = 6.88 Hz)	8.27 (dd, J = 2.20; 2.16 Hz)	3.08 (s)
*N*-Me-2PY * (DMSO-*d_6_*) [29]	9.09 (brs, 1H, H-Nb), 7.57 (brs, 1H, H-Na)			8.31 (dd, J = 7.21, 2.21 Hz)	6.47 (dd, J = 7.10, 7.10 Hz)	8.04 (dd, J = 6.55, 2.21 Hz)	3.56 (s)
*N*-Me-4PY (DMSO-*d_6_*)	9.36 (s), 7.54 (s)	8.54 (s)			6.53 (d, J = 7.4 Hz)	7.87 (d, J = 5.08 Hz)	3.82 (s)
*N*-Me-4PY * (DMSO-*d_6_*)^2^ [29]	9.56 (brs), 7.41 (brs)	7.74 (dd, J = 2.44, 7.49 Hz)	6.38 (d, J = 7.49 Hz)			8.44 (d, J = 2.44 Hz)	3.75 (s)
*N*- Me-6PY (D_2_O)		8.38 (s)		7.95 (d, 1H, J = 6.69 Hz)	6.53 (d, J = 9.44 Hz)		3.62 (s)
*N*- Me-6PY * (DMSO-*d_6_*) [29]	7.23 (brs), 7.69 (brs)		6.38 (d, J = 9.49 Hz)	7.85 (dd, J = 9.49, 2.54 Hz)		8.36 (d, J = 2.54 Hz)	3.46 (s)

*: non-IUPAC atom numbering.

**Table 3:** Tables of ^1^H NMR chemical shift for the ribosylated pyridones.

Compound (Solvent)	Chemical Shift (Multiplicity, J Values in Hz)											
	NH	H2	H3	H4	H5	H6	H_1’_	H_2’_	H_3’_	H_4’_	H_5’_	CH_3_
2PYR (D_2_O)				8.46 (dd, J = 5.4, 7.04 Hz, 2H, H4 & H6),			6.14 (s),	4.12 (bs, 3H, C_2__’__,3’,__4’_H)			3.97 (d, J = 12.9 Hz), 3.80 (d, J = 11.56 Hz)	
					6.55 (t, J = 6.96 Hz)							
2PYR (DMSO-*d_6_*) [32]	7.63 and 8.96 (2 brs, CONH_2_)			8.32 (m)	6.55 (t)	8.42 (m)	6.08 (d, J = 1.17 Hz)	5.55 (d, 1H (C_2__’_OH)	5.10 (d, 1H, C_3__’_OH)		5.23 (t, 1H, C_5__’_OH), 3.64 (m)	
								3.79–3.97 (m, 3H)				
4PYR (D_2_O)		8.67 (d, J = 2.24 Hz)			6.65 (d, J = 7.6 Hz)	7.94 (dd, J = 2.24 Hz, 2.24 Hz)	5.55 (d, J = 5.5 Hz)	4.23 (t, J = 5.28 Hz)	4.19–4.13 (m)		3.81 and 3.73 (ABX, 2H, JAB = 12.7 Hz, JAX = 3.04 Hz, JBX = 4.0 Hz)	
4PYR (D_2_O) [25]		8.66 (d, J = 2.4 Hz)			6.58 (d, J = 7.8)	7.93 (dd, J = 7.8, 2.4 Hz	5.53 (d, J = 5.9)	4.22 (t, J = 5.4)	4.16 (dd, J = 5.1, 3.5)	4.14 (ddd, J = 4.0, 3.5, 3.0 Hz)	3.72 (dd, J = 12.6, 3.0 Hz), 3.79 (dd, J = 12.6, 3.0 Hz)	
6PYR (D_2_O)		8.60 (s)		7.84 (d, J = 9.2 Hz)	6.54 (d, J = 9.44 Hz)		6.01 (s)	4.19–4.13 (m)			3.97 (d, J = 12.92 Hz), 3.80 (d, J = 12.88 Hz)	
6PYR (DMSO-*d_6_*) [31]	7.54(1 H, Amid-NH), 7.30 (s)	8.52 (d, J = 1.9 Hz)		7.81–7.86 (dd, J = 1.9 Hz, 9.5 Hz)	6.40 (d, J = 9.5 Hz)		6.01 (d, J = 3.5 Hz)	5.45 (d, J = 4.6 Hz,1H, C_2__’_ OH)	5.07 (d, J = 4.9 Hz, 1 H, C_3__’_ OH)		5.17 (t, J = 4.5 Hz, C_5__’_OH, 3.57–3.76 (m_b_)	
								3.92–4.08 (m, 3H, C_2__’__,3’,__4’_H)				
4PYR ACID (D_2_O)		8.21 (d, J = 2.4 Hz)			6.52 (d, J = 7.6 Hz)	7.89 (dd, J = 7.6, 2.4 Hz)	5.51 (d, J = 5.6 Hz)	4.26 (dd, J = 5.6 and 5.3 Hz)	4.20 (dd, J = 5.3 and 3.6 Hz)	4.15 (dd, J = 8.3, 3.6 Hz)	3.82 (dd, J = 12.8, 4.5 Hz), 3.74 (dd, J = 12.8, 4.5 Hz)	

## Data Availability

Raw NMR and MS data files can be obtained upon request made directly to M.M.

## Supplementary Materials

The following are available online at https://www.mdpi.com/1422-0067/22/3/1145/s1. Figure S1: ^1^H NMR, ^13^C NMR and HRMS Spectra. Figure S2: NQO2 catalyzed 4PYR formation. Table S1: Isotopic pattern with relative abundance of pyridones and their intermediates.

## Abbreviations

minute (min), hour (h), room temperature (rt), methanol (MeOH), dimethylsufoxide (DMSO), acetonitrile (CH_3_CN, ACN), Hydroxybenzotriazole (HOBt), dimethylformamide (DMF), dicyclohexylcarbodiimide (DCC), dichloromethane (DCM), nicotinamide riboside (NR), nicotinamide riboside reduced form (NRH), nicotinamide adenine dinucleotide, (NAD), nicotinamide adenine dinucleotide phosphate (NADP), nicotinamide adenine dinucleotide reduced form, (NADH), nicotinamide adenine dinucleotide phosphate reduced form (NADPH), pyridone (PY), methyl-pyridones (*N*-Me-PY), pyridone ribosides (PYR), flavin adenine dinucleotide (FAD), Electrospray ionization (ESI), mass spectrometry (MS), liquid chromatography coupled with mass spectrometry (LS-MS), proton nuclear magnetic resonance spectroscopy (^1^H NMR), calculated (calcd).
