# Supplementary material for: The Biochemical Pathways of Nicotinamide-Derived Pyridones
Source: Int J Mol Sci. 2021 Jan 24;22(3):1145. doi: 10.3390/ijms22031145 (PMC7866226; doi:10.3390/ijms22031145)

SUPPLEMENTARY MATERIALS

Title: The (bio)chemical diagnosis of nicotinamide-derived pyridones

Authors

F. Hayat,[1,4] M. Sonavane,[1,2,4] M. V. Makarov, [4] S. Trammell,[3] P. McPherson,[4] N. R. Gassman,[2,4] and M. E. Migaud[1,4]

Affiliations:

1. Department of Pharmacology, College of Medicine, University of South Alabama, Mobile, AL, USA.
2. Department of Physiology, College of Medicine, University of South Alabama, Mobile, AL, USA.
3. Novo Nordisk Foundation, Center for Basic Metabolic Research, University of Copenhagen, Denmark.
4. Mitchell Cancer Institute, College of Medicine, University of South Alabama, 1660 Springhill Avenue, Mobile, 36604, AL, USA.

Table 1. Isotopic pattern with relative abundance of pyridones and their intermediates.

Compound name	Fragmentation Pattern [Relative Abundance (%)]							
	**MW+0**	**MW+1**	**MW+2 from natural isotopic abundance [in source acylium products]**	**MW+3**	**MW+ Na**	**MW + CH_3CN**	**MW+Na +CH_3CN**	**[M-CH_3]$^+$**
1-N-methyl-4-oxo-pyridine-3-carboxamide = 1-N-methyl-4-pyridone-3-carboxamide	HRMS $C_7H_8N_2O_2$	[$C_7H_9N_2O_2$]$^+$ 153.0652 (100%)	154.0491* (~10%) [$C_7H_8NO_3^+$] (~10%)					
O-methyl 4-pyridone-3-carboxylate	HRMS $C_7H_7NO_3$	[$C_7H_8NO_3^+$] 154.0490 (100%)	155.0522 (10%)					140.0335 (100%)
O-methyl, 1-N-methyl 4-pyridone-3-carboxylate	HRMS $C_8H_9NO_3$	[$C_8H_{10}NO_3^+$] 168.0648 (100%)	169.0681 (10%)					154.0492 (100%)
O-methyl-1-methyl-6-oxo-pyridine-3-carboxylate	HRMS $C_8H_9NO_3$	[$C_8H_{10}NO_3^+$] 168.0647 (100%)					209.69 (100%)	
1-N-methyl-6-pyridone-3-carboxamide	HRMS $C_7H_8N_2O_2$	[$C_7H_9N_2O_2$]$^+$ 153.0663 (100%)	154.0696 (5%)					
1-N-methyl-2-pyridone-3-carboxamide	HRMS	[$C_7H_9N_2O_2$]$^+$ 153.0648 (100%)	154.0487* (~10%) [$C_7H_8NO_3^+$] (~10%)					
4-pyridone-3-carboxamide	HRMS $C_6H_6N_2O_2$	[$C_6H_7N_2O_2$]$^+$ 139.0495 (100%)	140.0334 (~10%) [$C_6H_6NO_3^+$] (~10%)					
1-(2′,3′,5′-tri-O-acetyl-β-D-ribofuranosyl)-4-pyridone-3-carboxamide	HRMS $C_{17}H_{20}N_2O_9$	[$C_{17}H_{21}N_2O_9$]$^+$ 397.1228 (100%)	398.1220 (20%)	399.1276 (1%)				

4-pyridone-3-carboxamide-1-β-D-ribofuranoside	HRMS $C_{11}H_{14}N_2O_6$	$[C_{11}H_{15}N_2O_6]^+$ 271.0918 (100%)	272.0951 (10%) $[C_{11}H_{14}NO_7]^+$ 272.0770 not selected			
6-pyridone 3-carboxamide	HRMS $C_6H_6N_2O_2$	$[C_6H_7N_2O_2]^+$ 139.0499 (100%)	140.0532 (5%)			
1-(2′,3′,5′-tri-O-acetyl-β-D-ribofuranosyl)-6-pyridone-3-carboxamide	MS (ES)	397.63 (32%)			419.56 (55%)	460.67 (30%)
6-pyridone-3-carboxamide-1-β-D-ribofuranoside	HRMS $C_{11}H_{14}N_2O_6$	$[C_{11}H_{15}N_2O_6]^+$ 271.0938 (100%)	272.0971 (10%)			
1-(2′, 3′, 5′-tri-O-acetyl-β-D-ribofuranosyl)-4-pyridone-3-carboxylic methyl ester	HRMS $C_{17}H_{20}N_2O_9$	$[C_{17}H_{21}N_2O_9]^+$ 412.1244 (100%)	413.1275 (20%)	414.1292 (2%)		
O-methyl-4-pyridone-3-carboxylate ribonucleoside	HRMS $C_{12}H_{15}NO_7$	$[C_{12}H_{16}NO_7]^+$ 286.0929 (100%)	287.0960 (10%)			
4-pyridone-3-carboxylic acid ribonucleoside	HRMS $C_{11}H_{13}NO_7$	$[C_{11}H_{14}NO_7]^+$ 272.0768 (100%)	273.0801 (10%)			
2-pyridone-3-carboxamide	HRMS $C_6H_6N_2O_2$	$[C_6H_7N_2O_2]^+$ 139.0493 (100%)	140.0333 (~10%) $[C_6H_6NO_3^+]$ (~30%)			
N-methyl-2-pyridone-3-carboxamide	HRMS $C_7H_8N_2O_2$	$[C_7H_9N_2O_2]^+$ 153.0648 (100%)	154.0887 (~10%) $[C_7H_8NO_3]^+$ (~10%)			
2-pyridone-3-carboxamide riboside	HRMS $C_{11}H_{14}N_2O_6$	$[C_{11}H_{15}N_2O_6]^+$ 271.0909 (100%)	272.0941 (~10%) $[C_{11}H_{14}NO_7]^+$ 272.0770 not selected	273.0958 (~2%)		

Figure S1: ^{1}H NMR, ^{13}C NMR and HRMS spectra

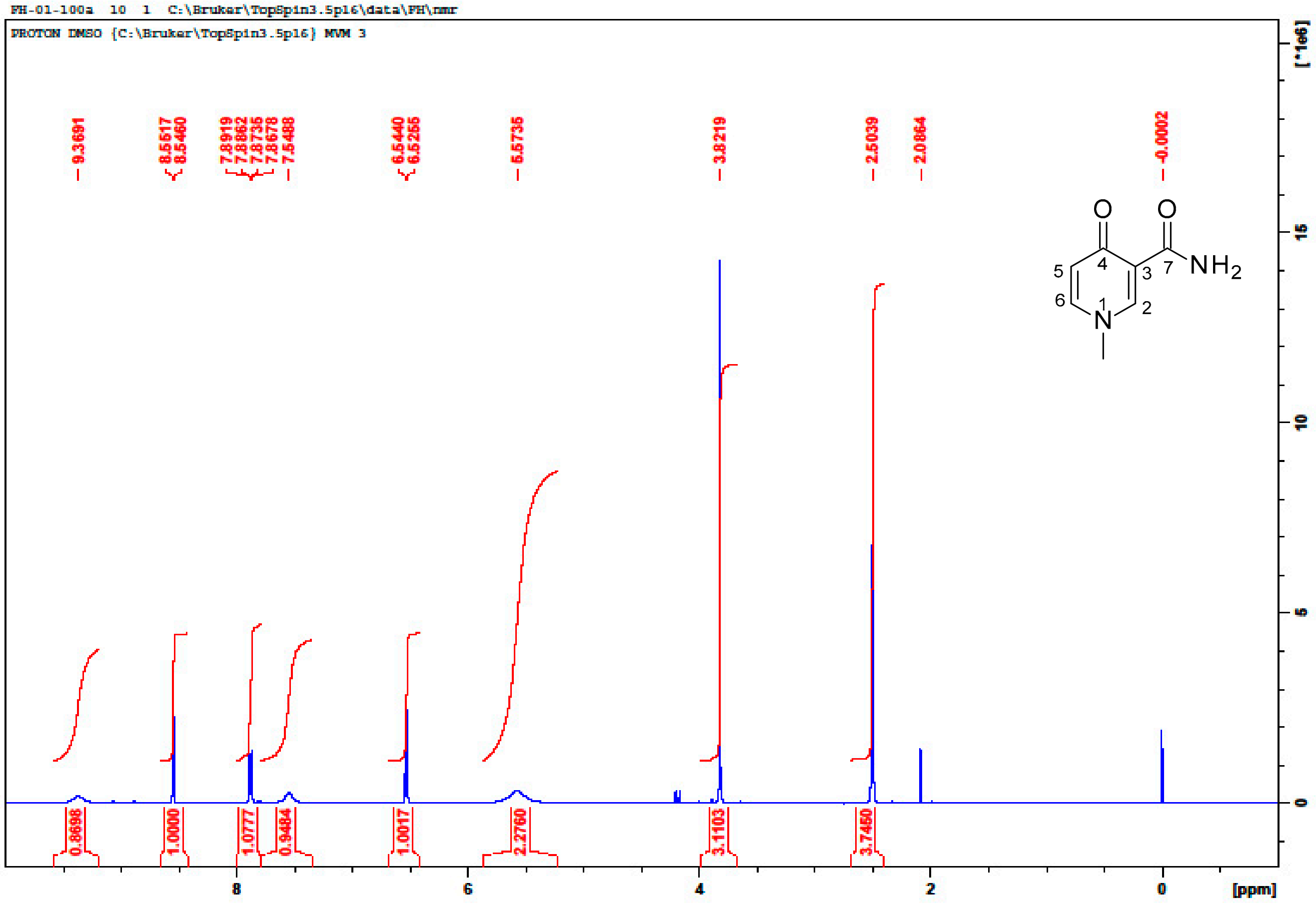

Compound 1. 400 MHz ^{1}H NMR spectrum in DMSO

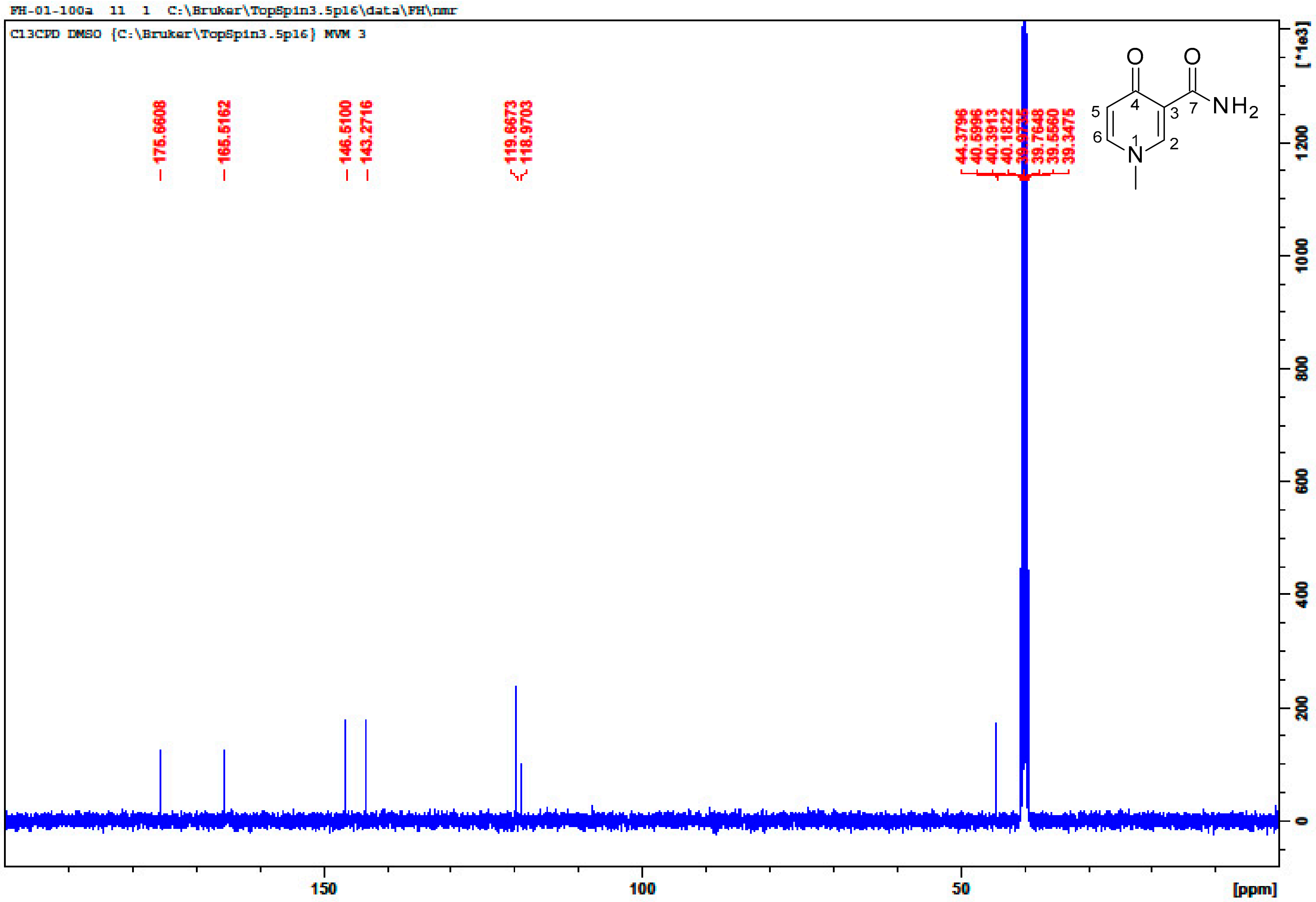

Compound 1. 400 MHz ^{13}C NMR spectrum in DMSO

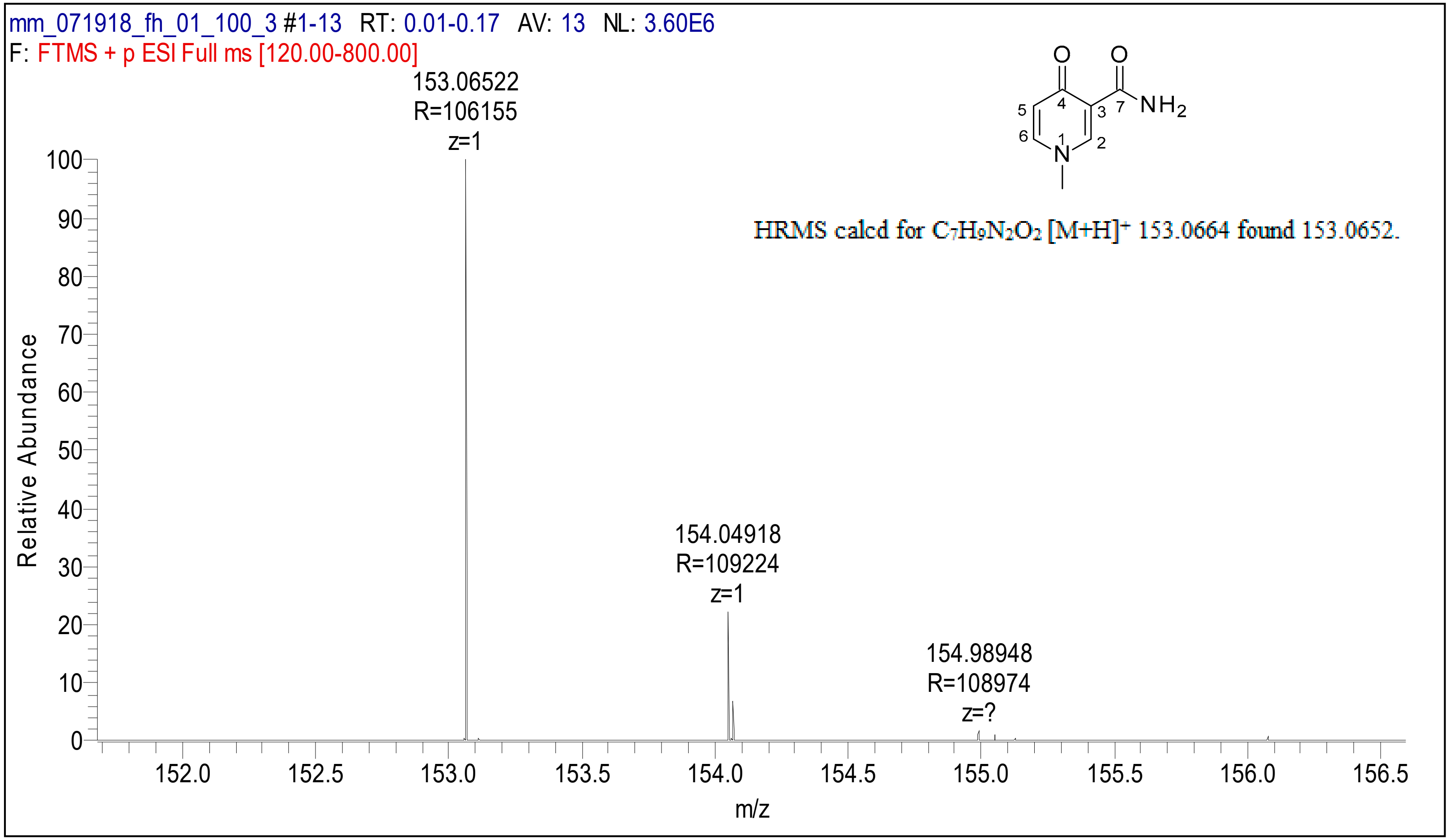

Compound 1. HRMS spectra

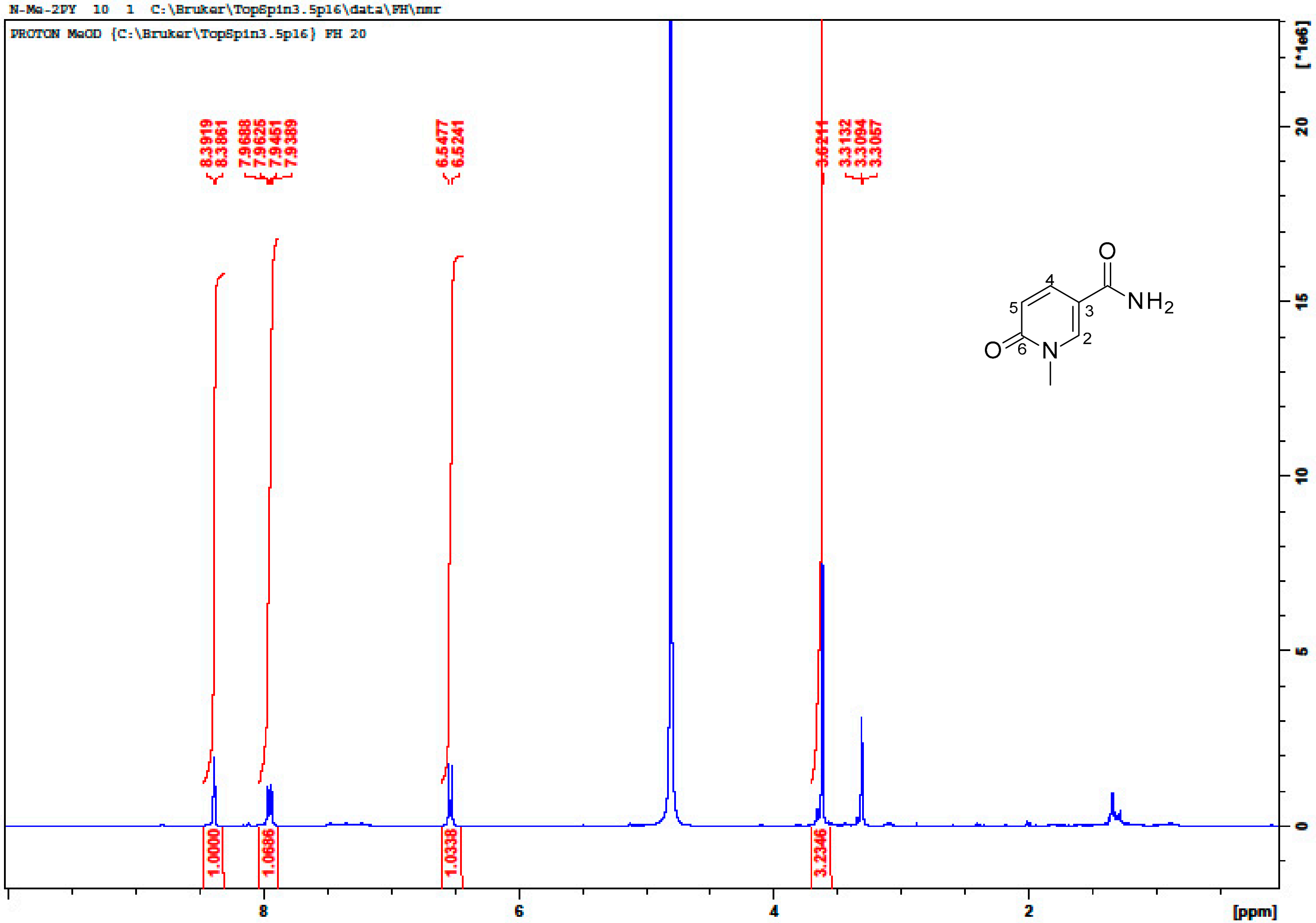

Compound 2. 400 MHz ^{1}H NMR spectrum in MeOD

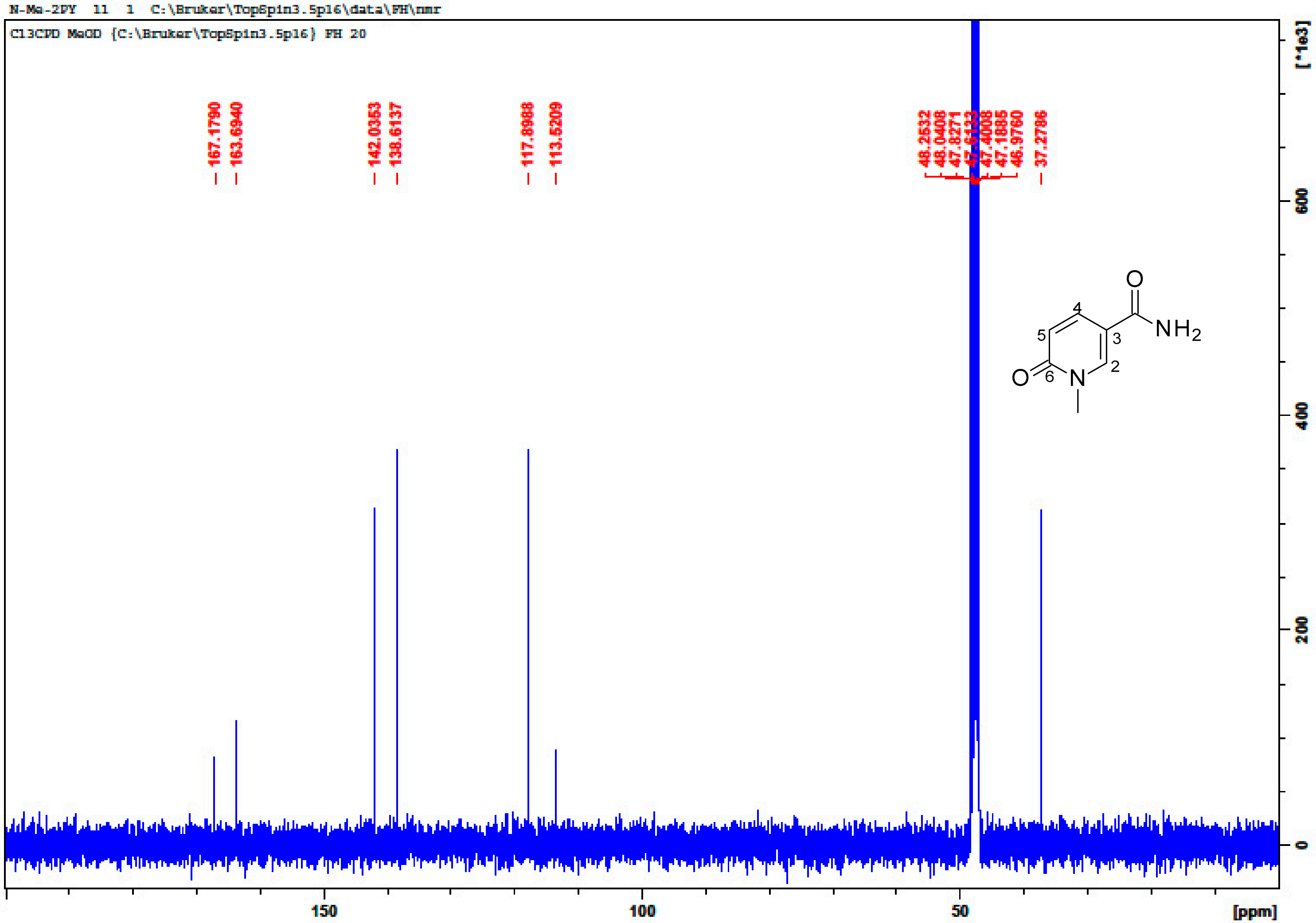

Compound 2. 400 MHz ^{13}C NMR spectrum in MeOD

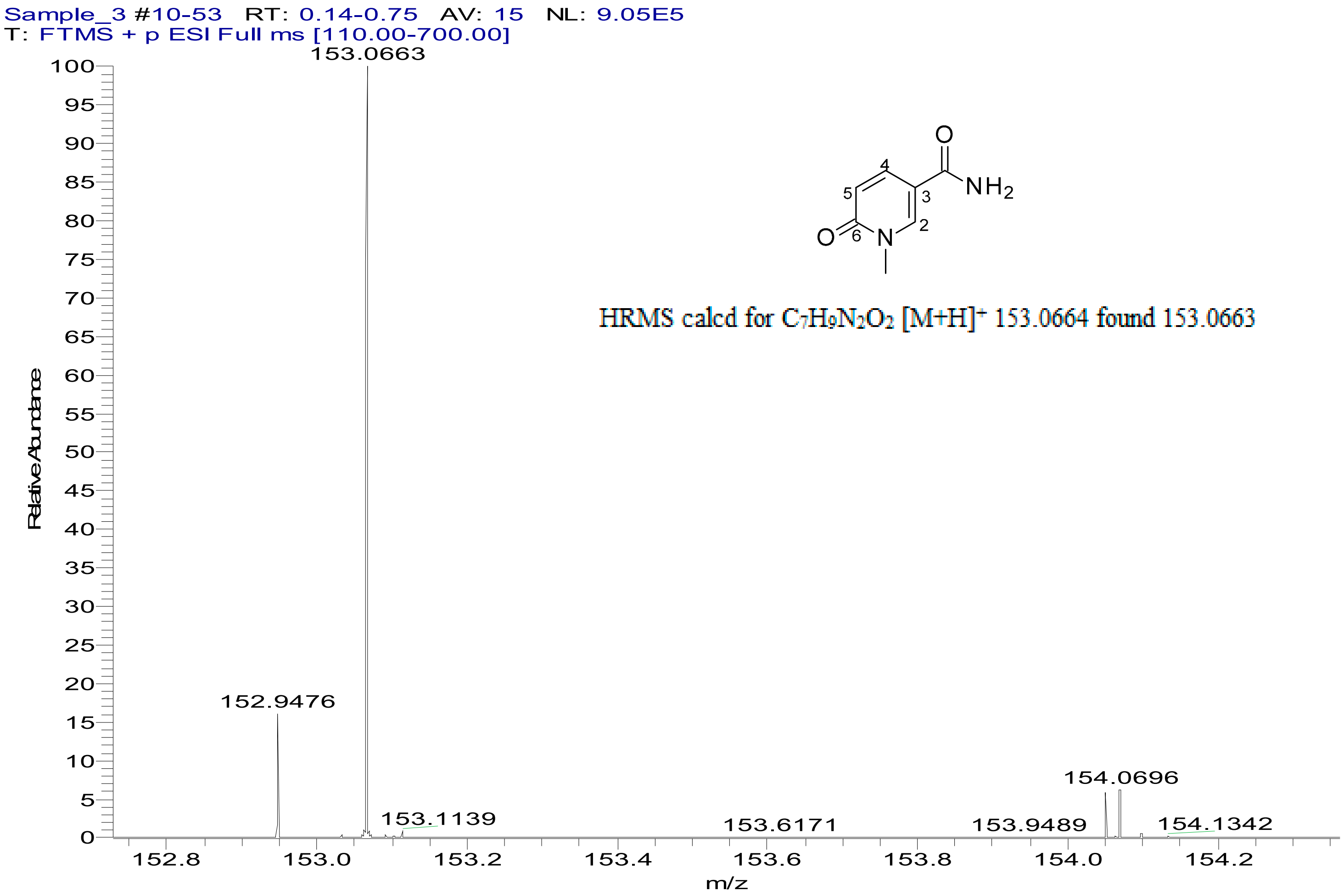

Compound 2. HRMS spectra

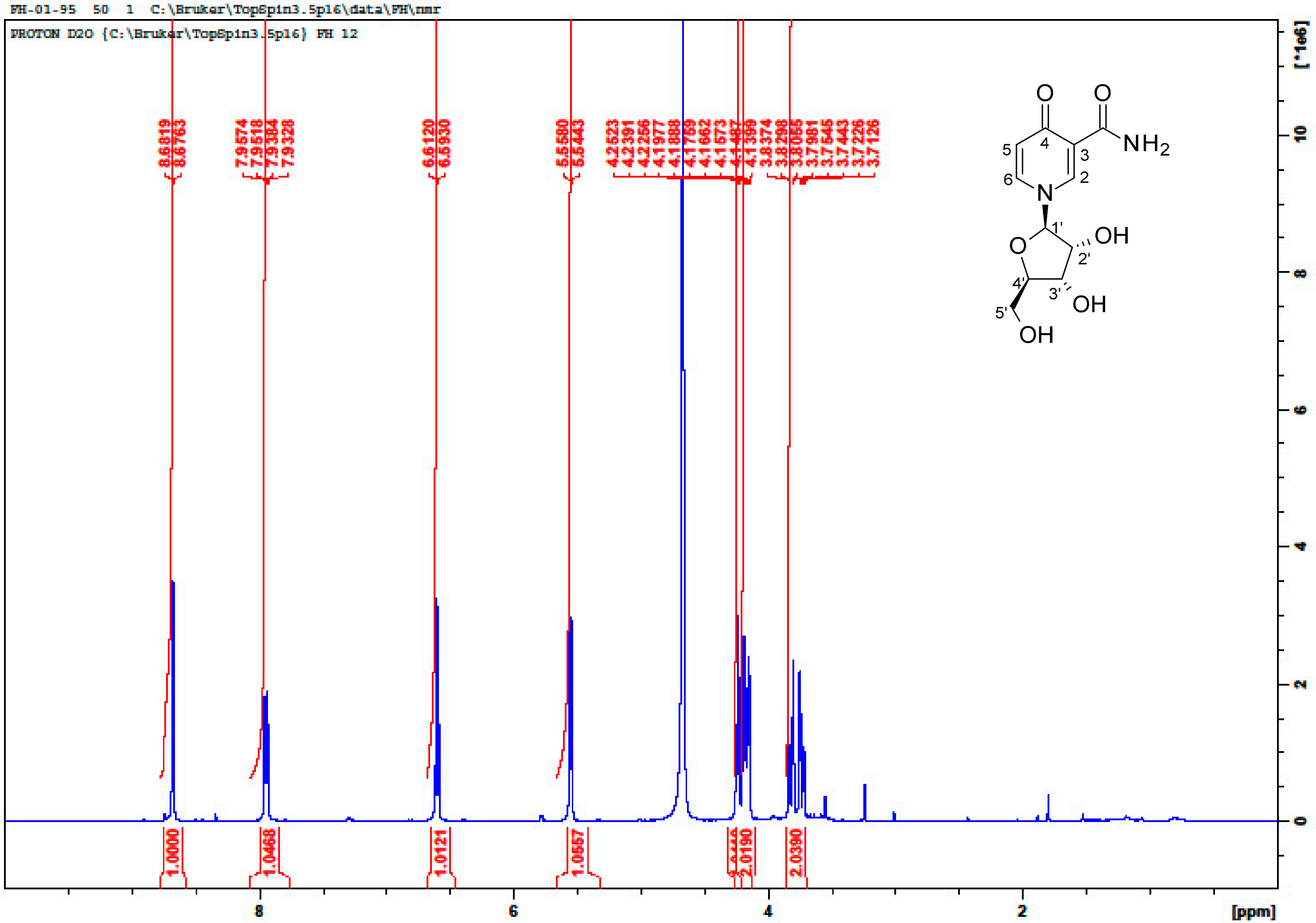

Compound 3. 400 MHz ^{1}H NMR spectrum in D_2O

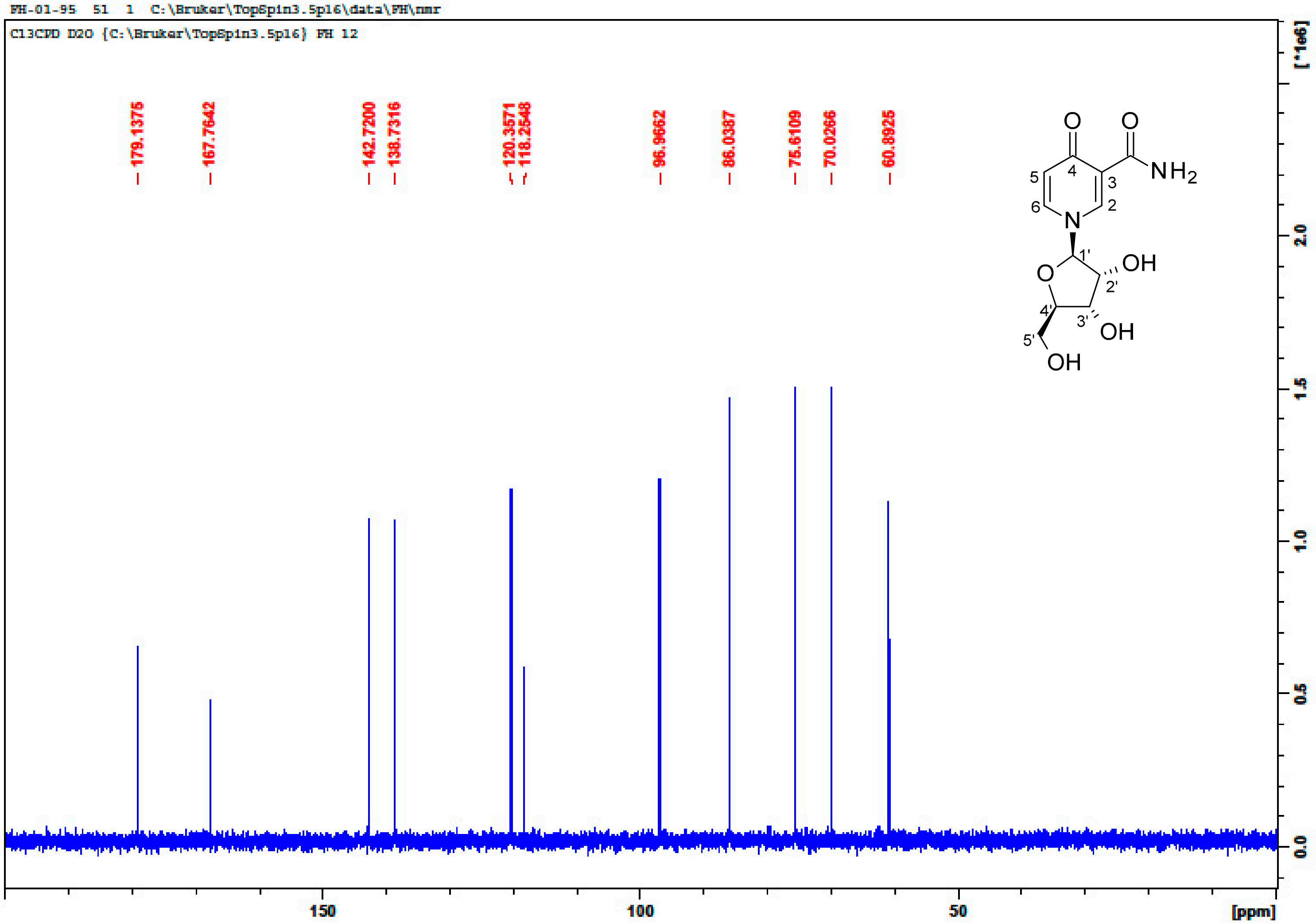

Compound 3. 400 MHz ^{13}C NMR spectrum in D_2O

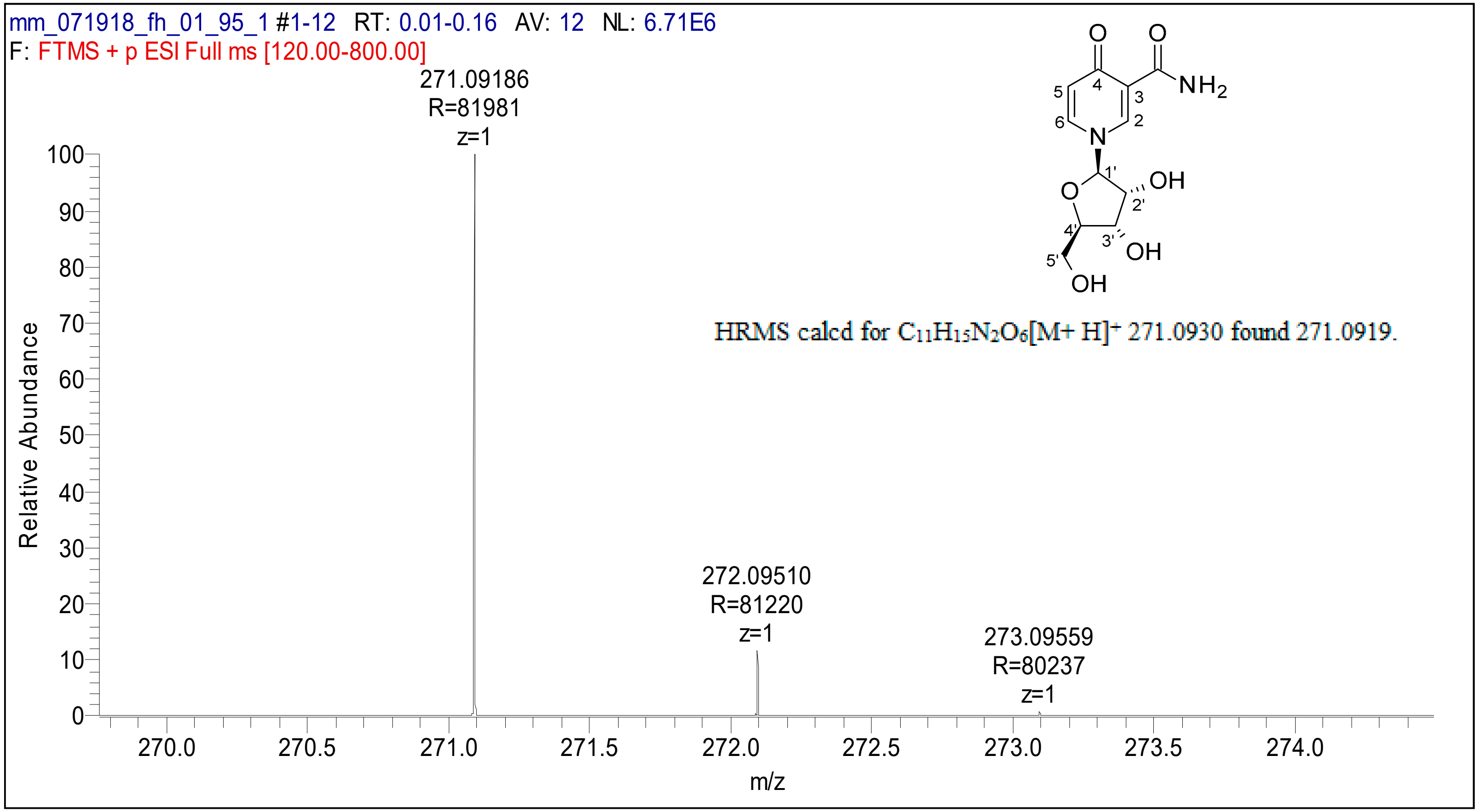

Compound 3. HRMS spectra

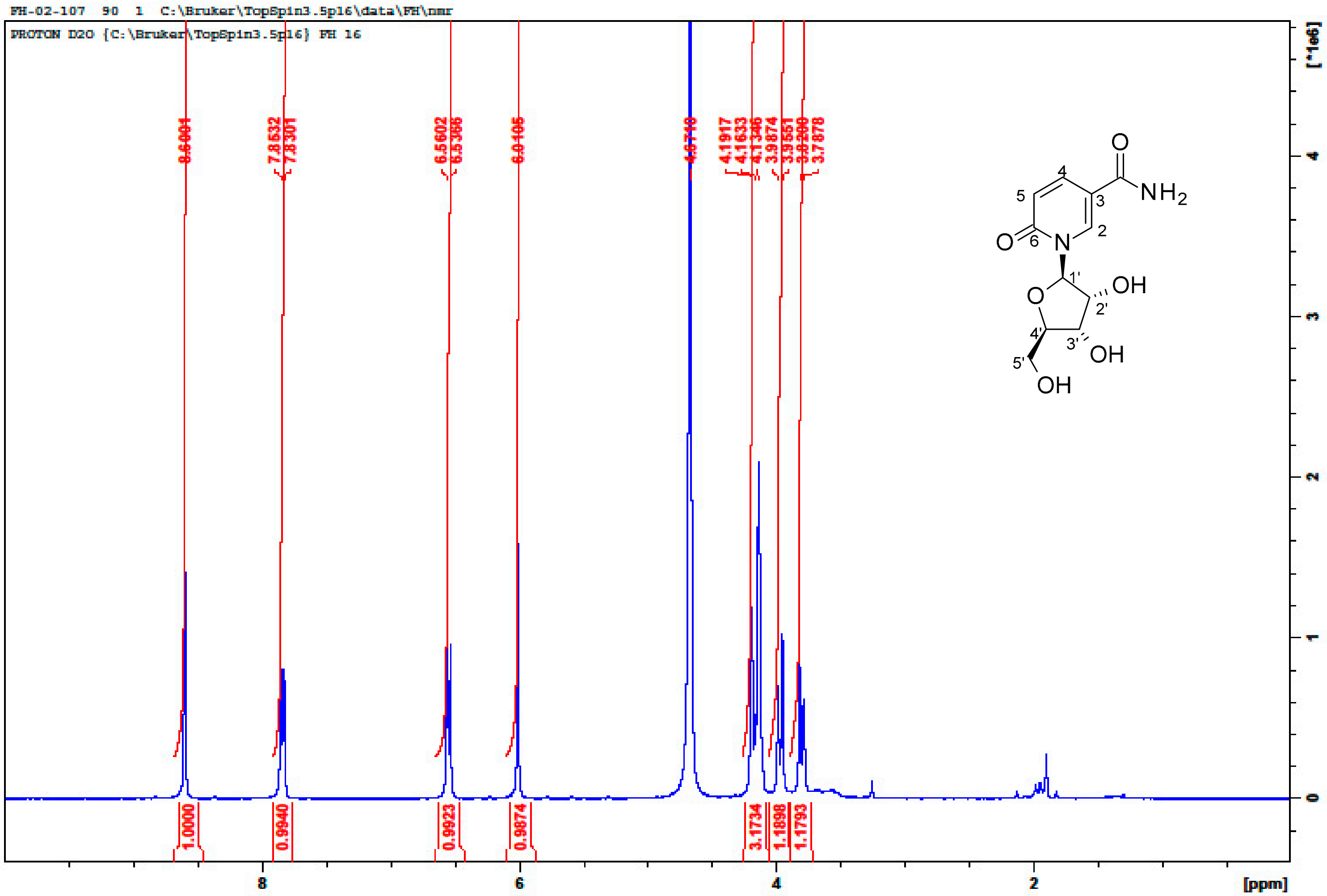

Compound 4. 400 MHz ^{1}H NMR spectrum in D_2O

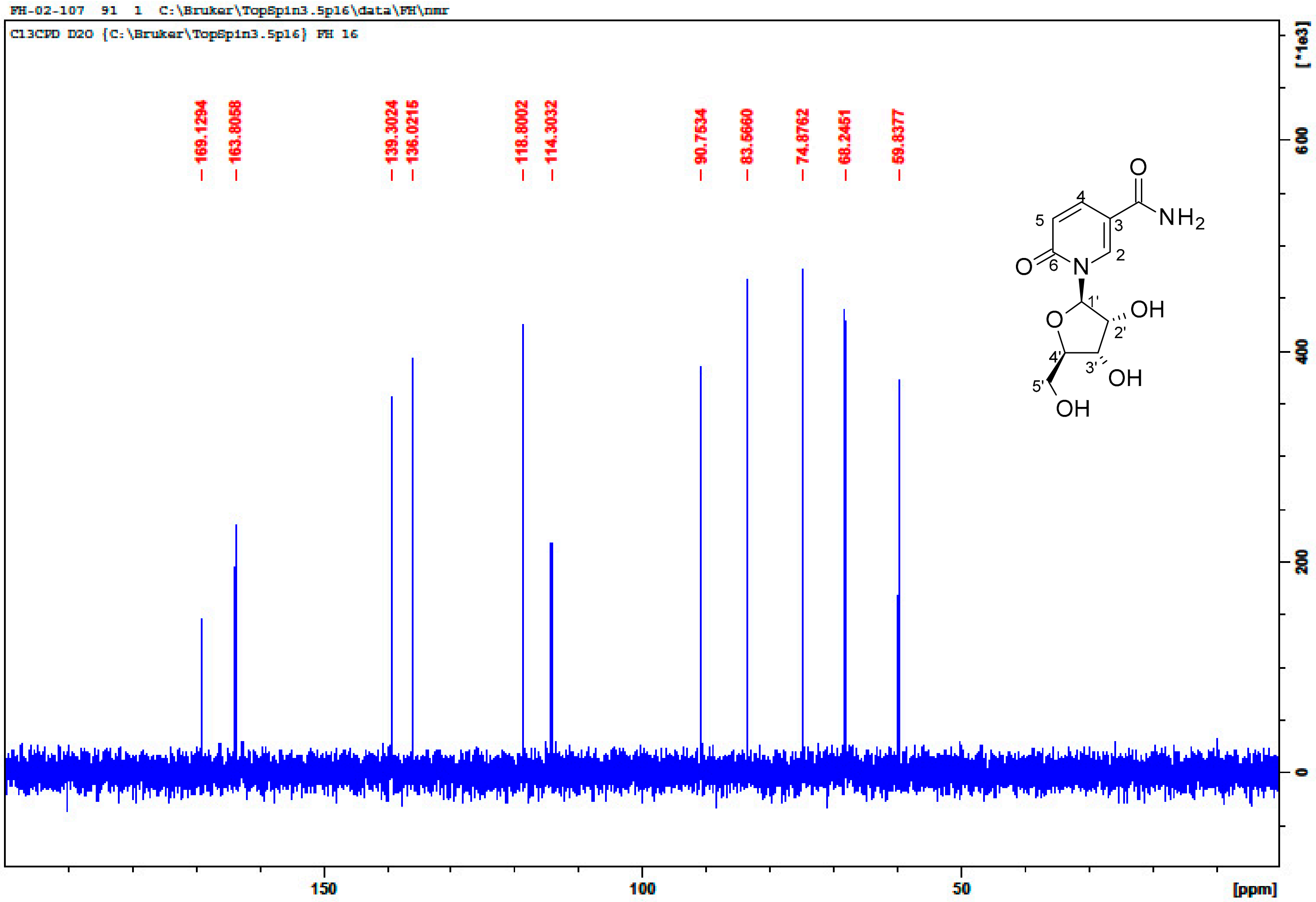

Compound 4. 400 MHz ^{13}C NMR spectrum in D_2O

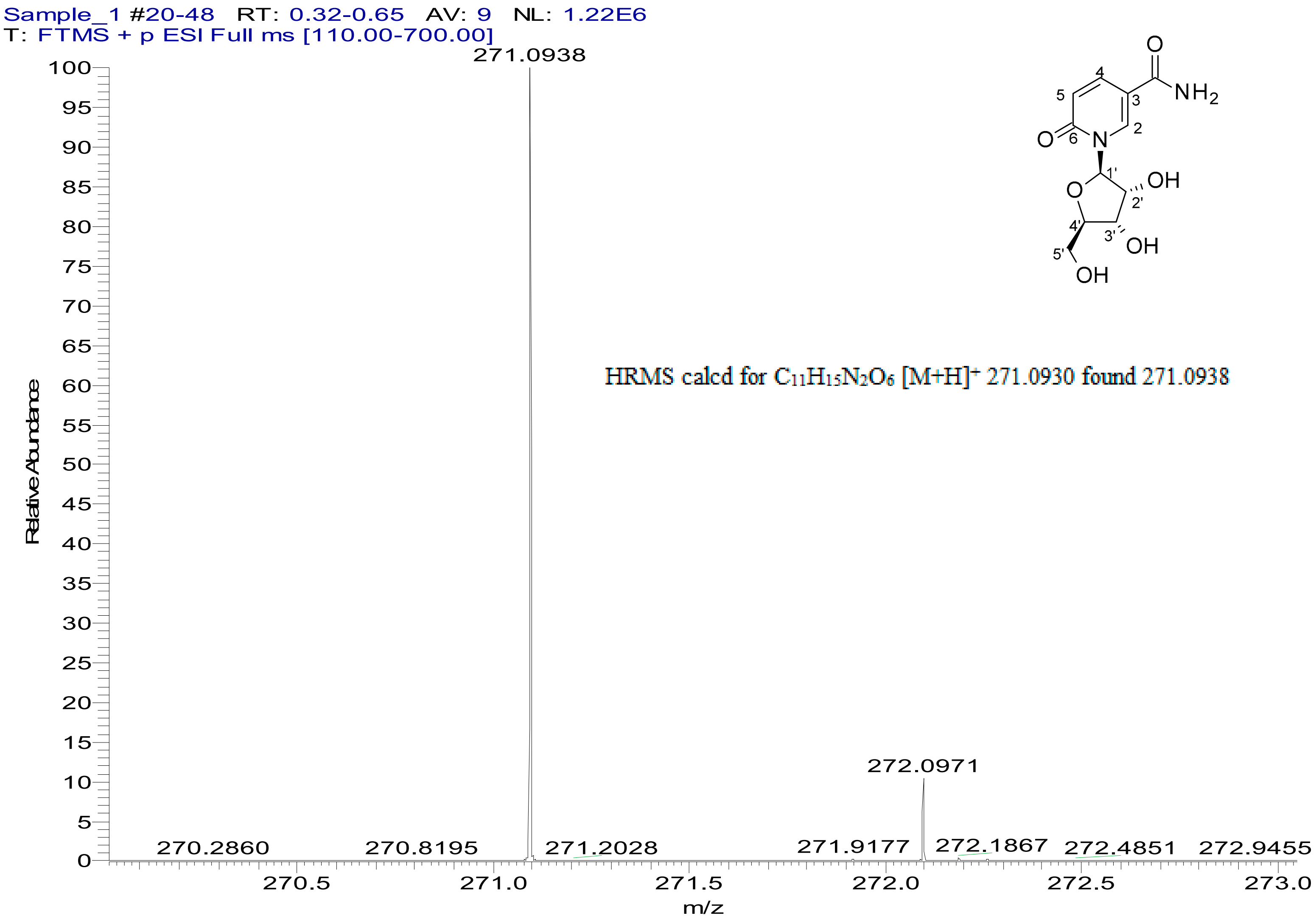

Compound 4. HRMS spectra

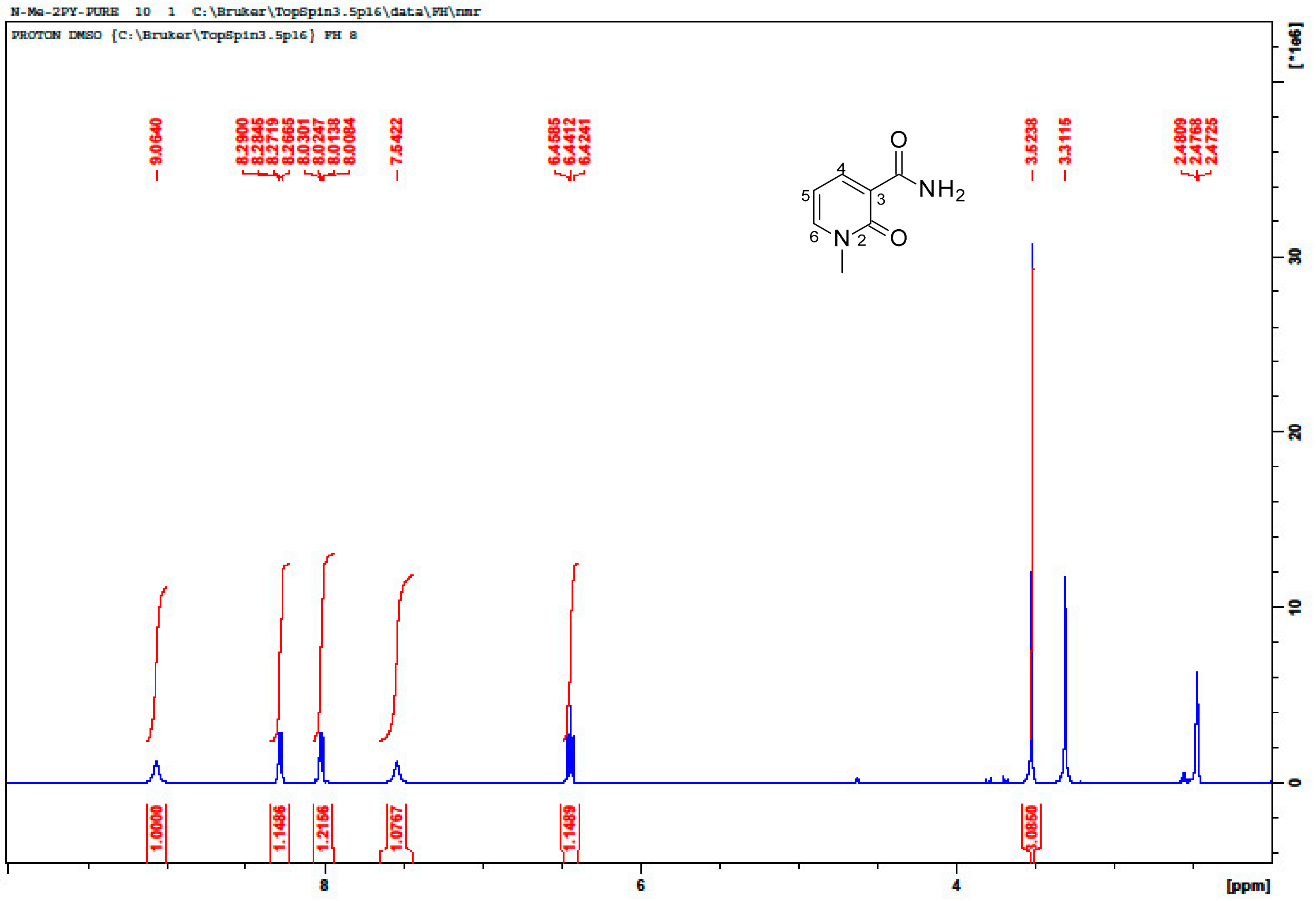

Compound 5. 400 MHz ^{1}H NMR spectrum in DMSO

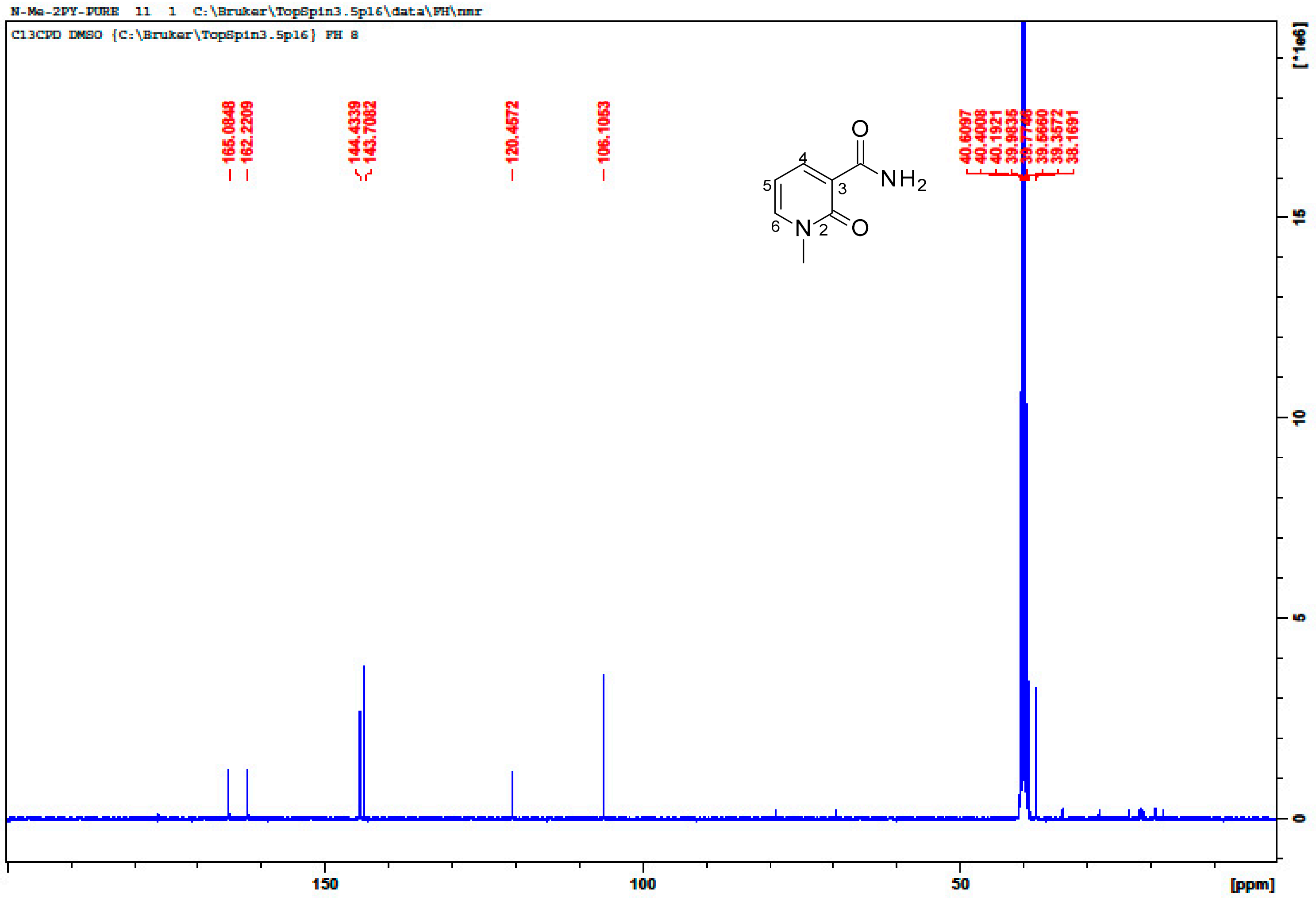

Compound 5. 400 MHz ^{13}C NMR spectrum in DMSO

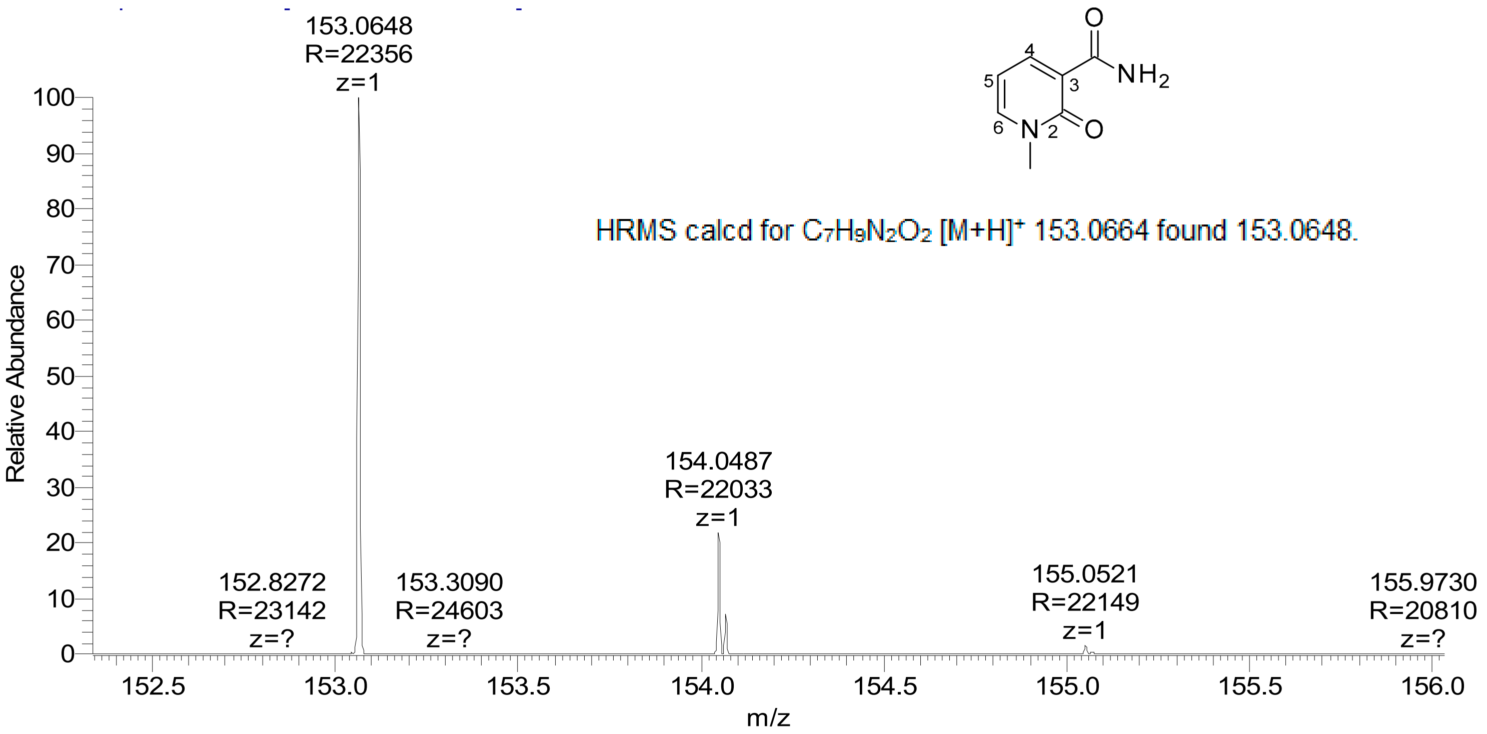

Compound 5. HRMS spectra

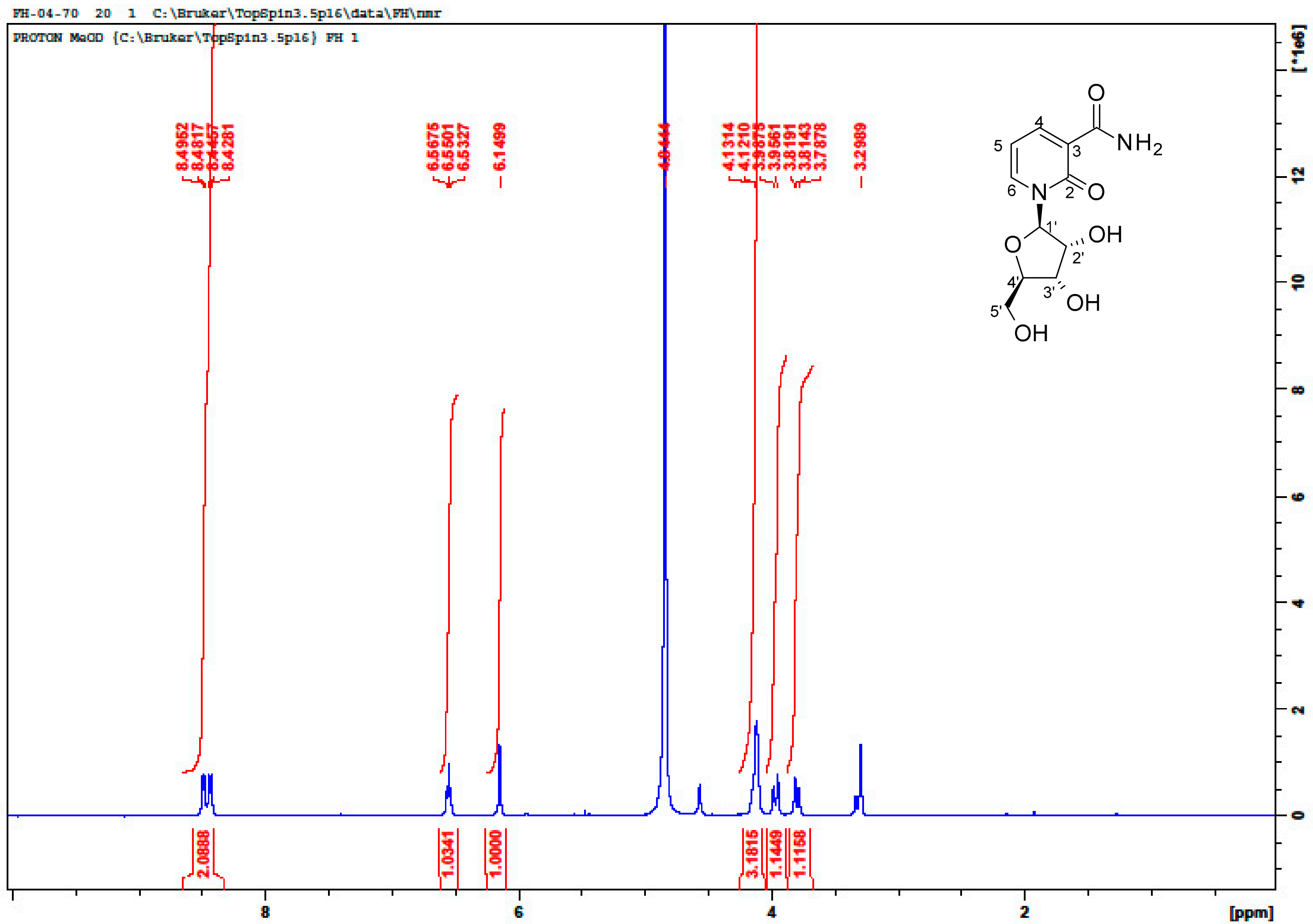

Compound 6. 400 MHz ^{1}H NMR spectrum in D_2O

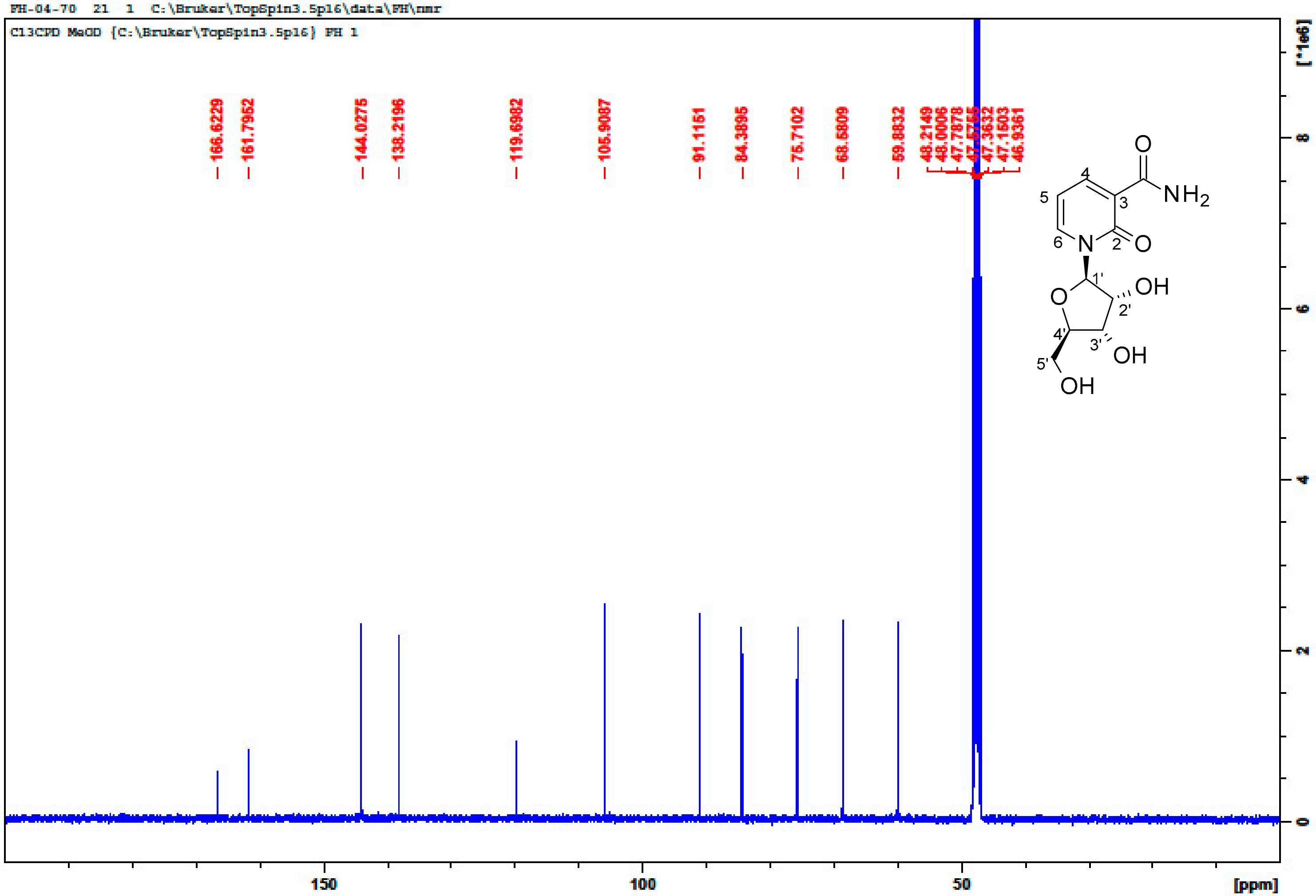

Compound 6. 400 MHz ^{13}C NMR spectrum in D_2O

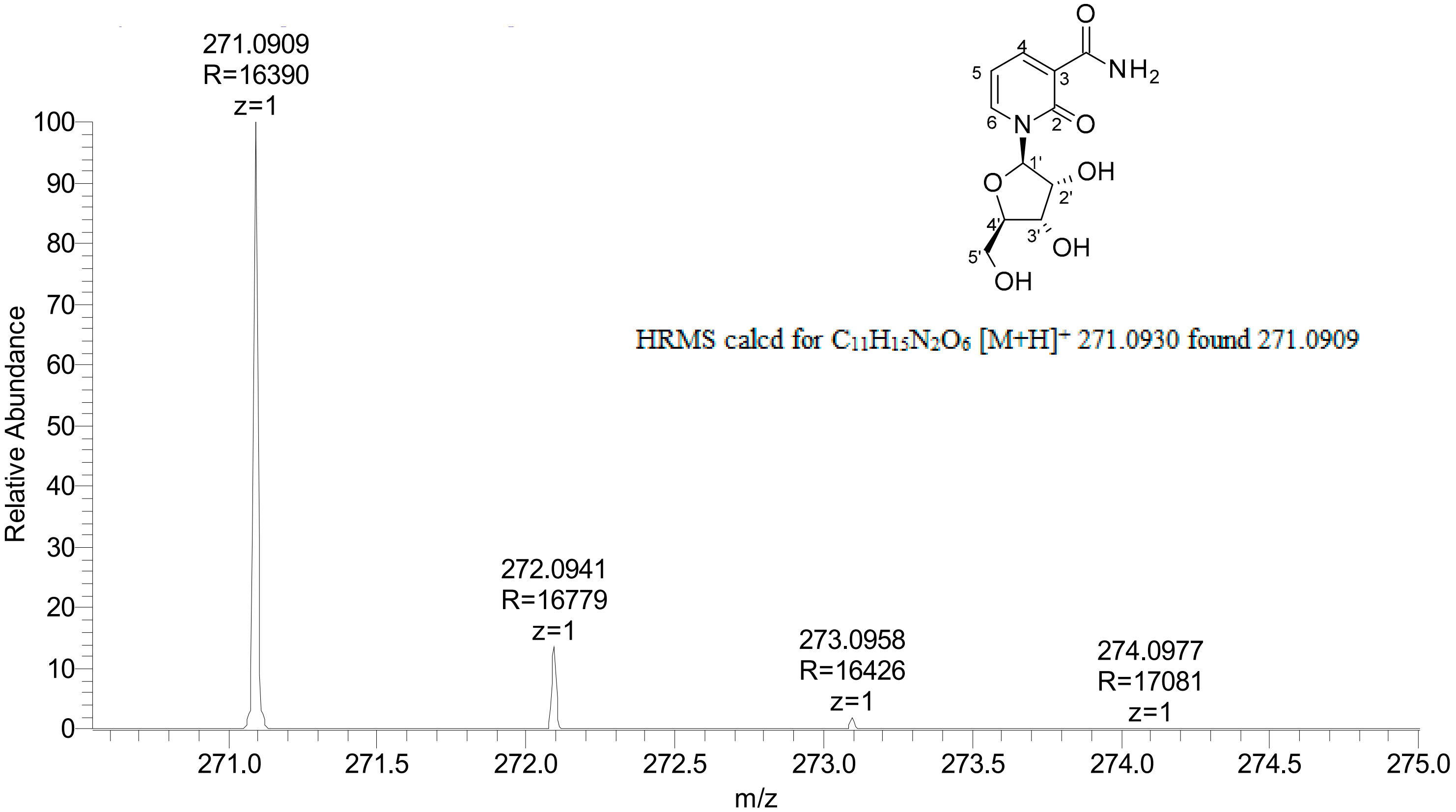

Compound 6. HRMS spectra

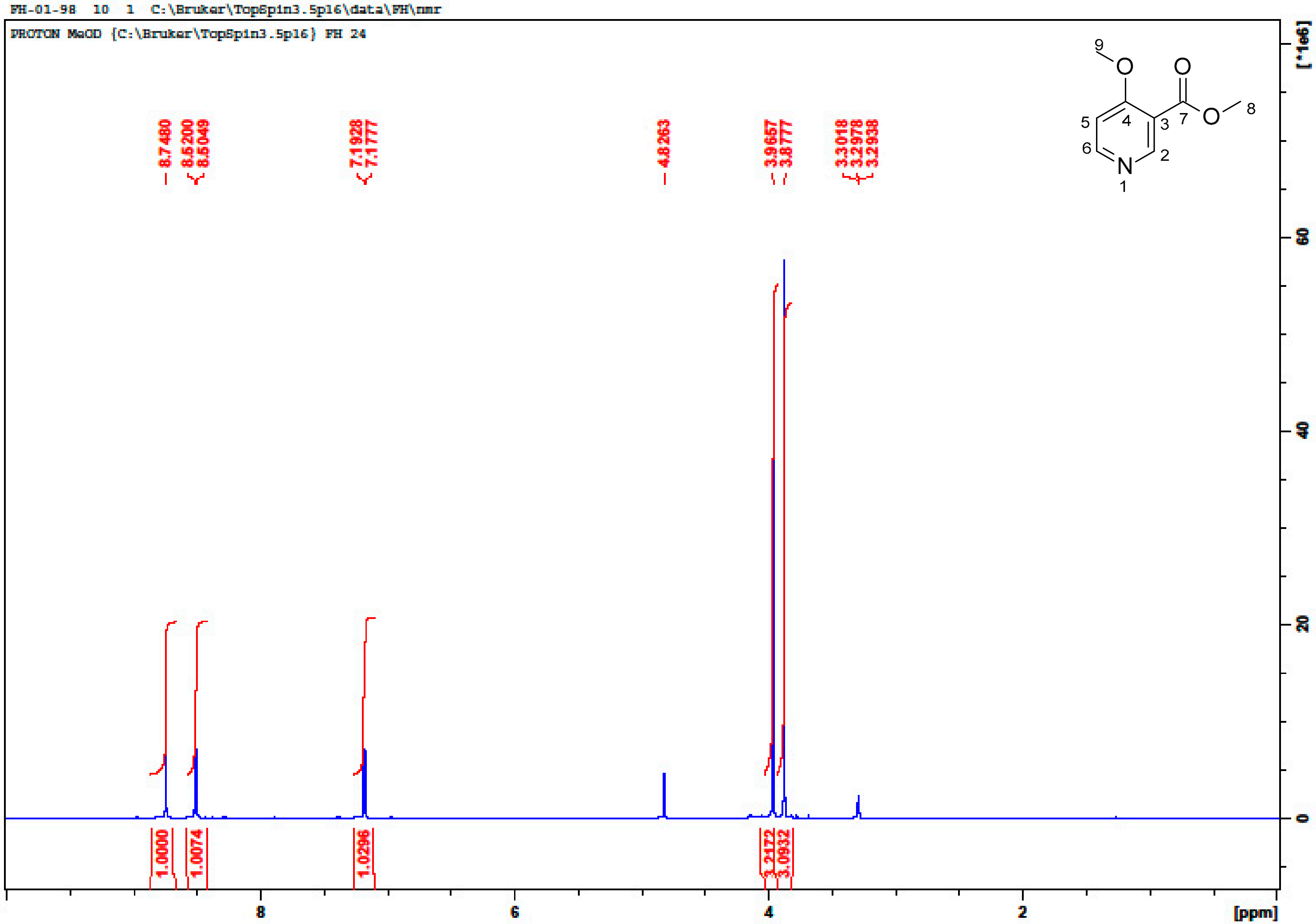

Compound 9. 400 MHz ^{1}H NMR spectrum in MeOD

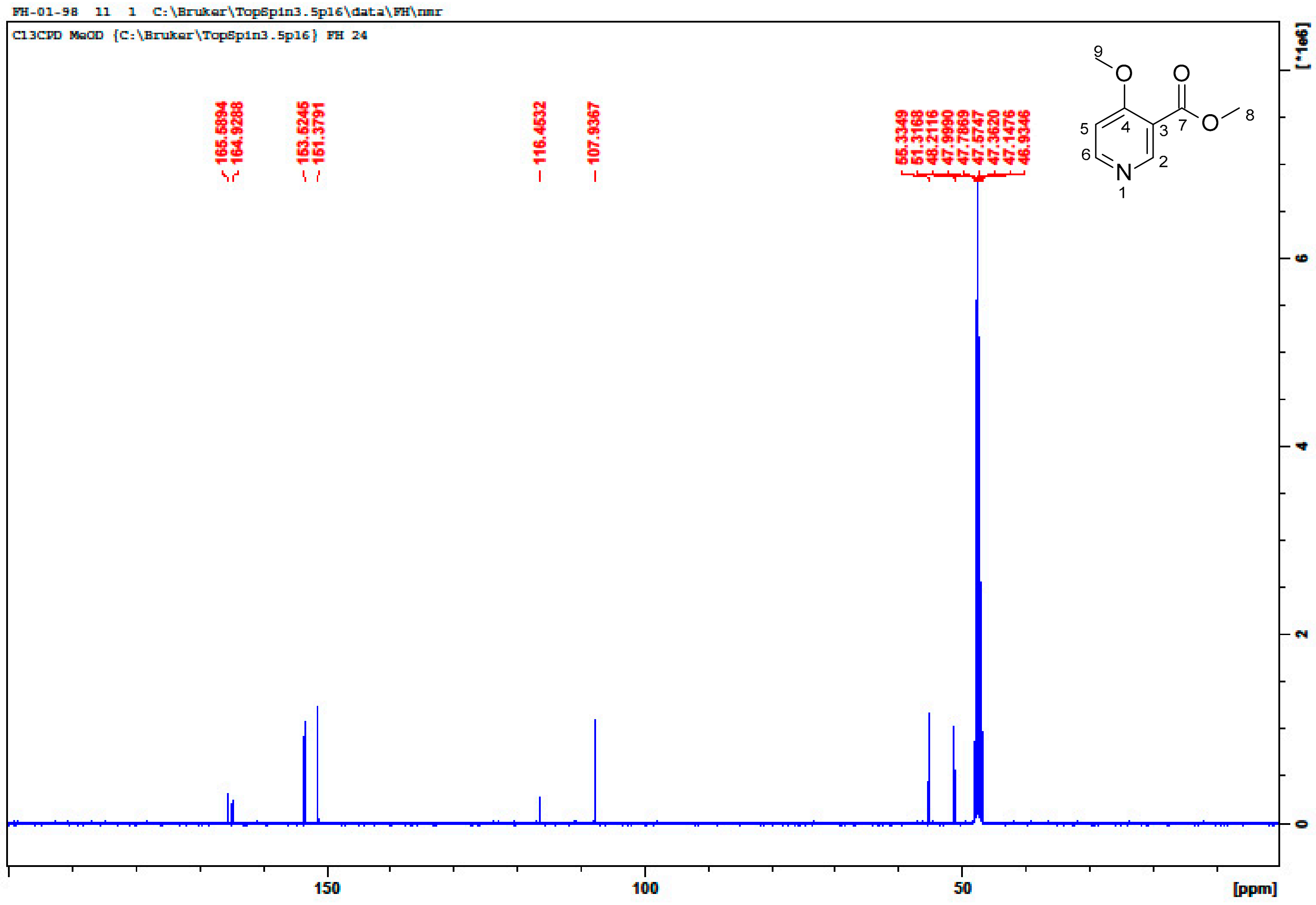

Compound 9. 400 MHz ^{13}C NMR spectrum in MeOD

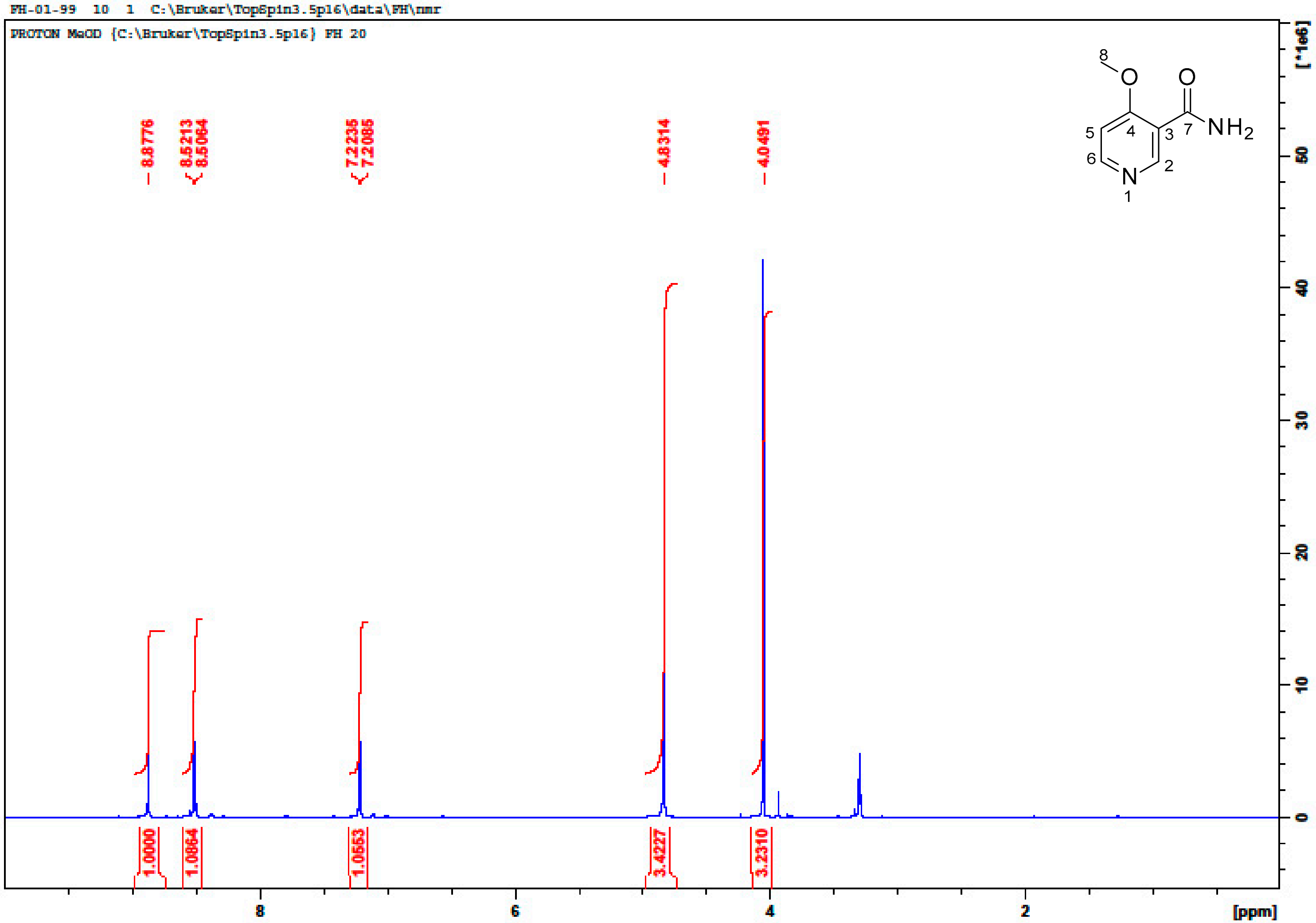

Compound 10. 400 MHz ^{1}H NMR spectrum in MeOD

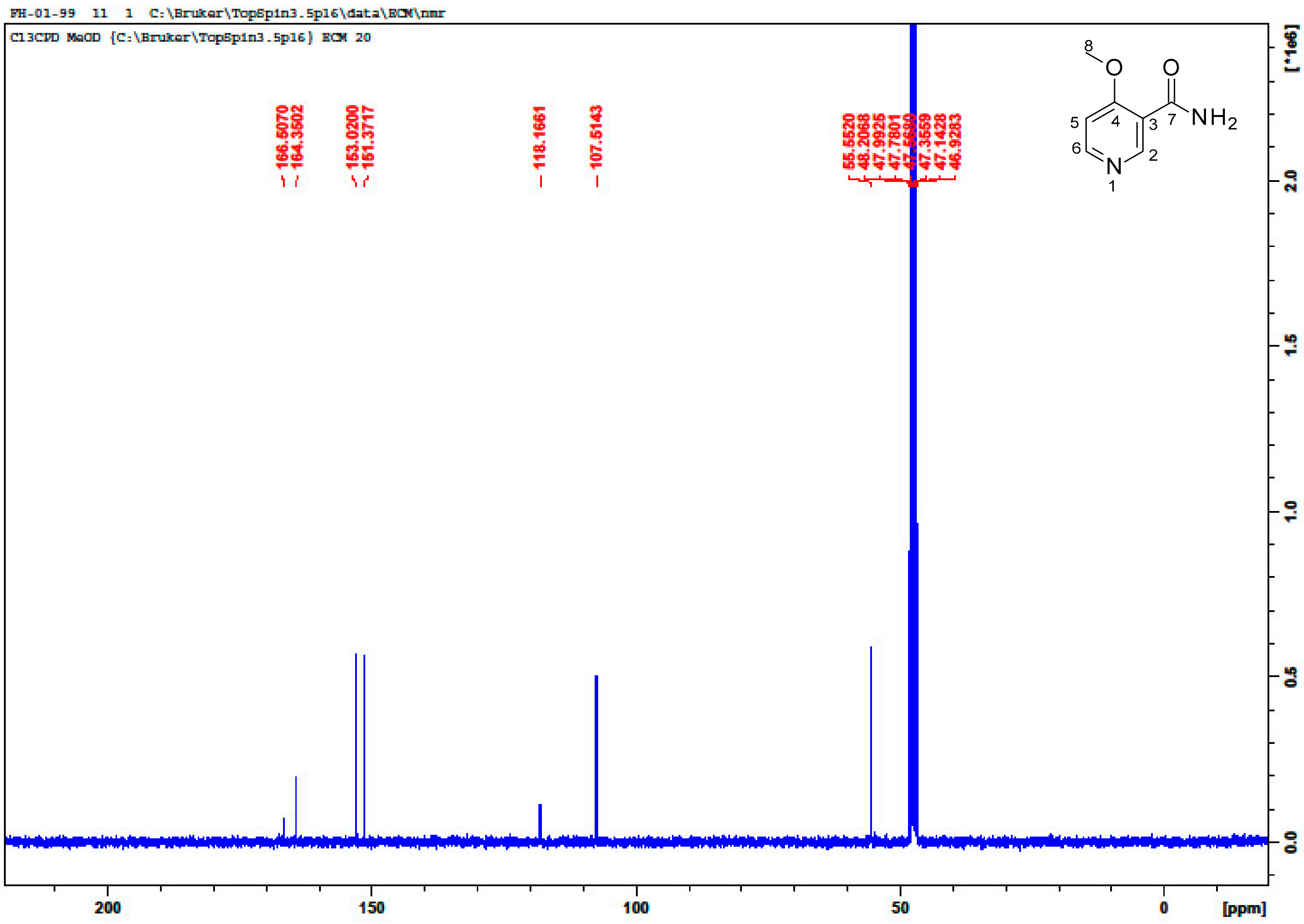

Compound 10. 400 MHz ^{13}C NMR spectrum in MeOD

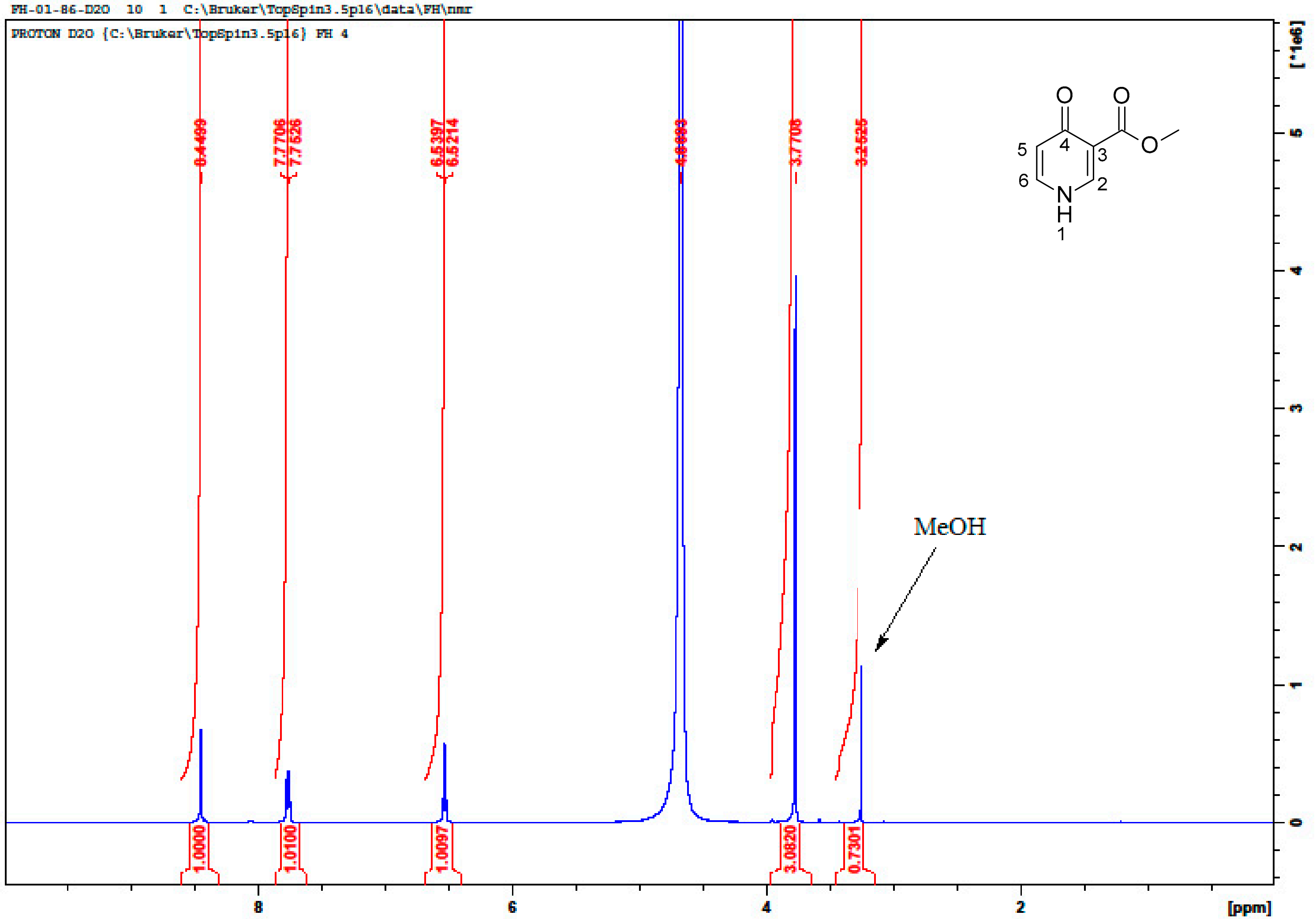

Compound 11. 400 MHz 1H NMR spectrum in D_2O

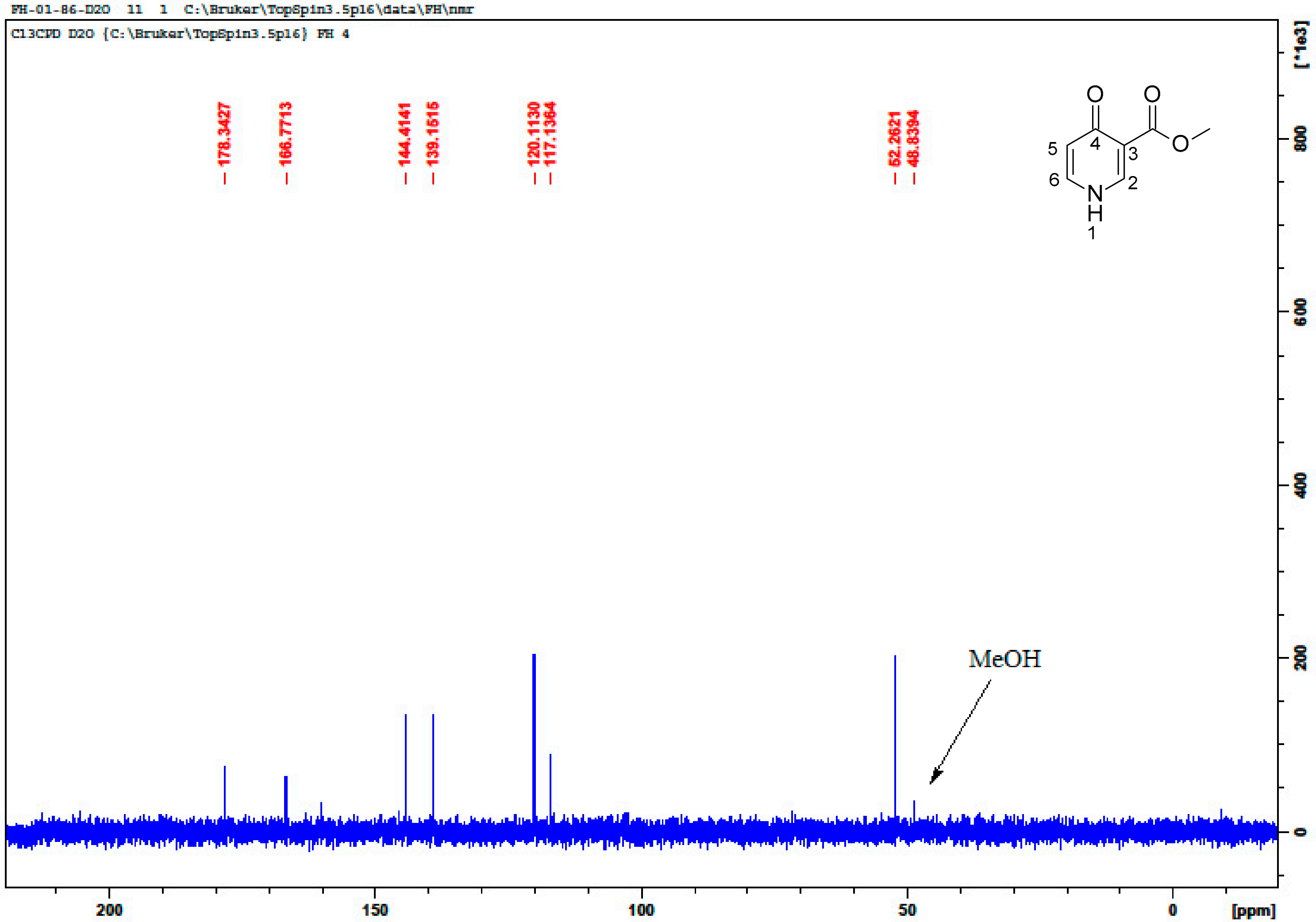

Compound 11. 100 MHz ^{13}C NMR spectrum in D_2O

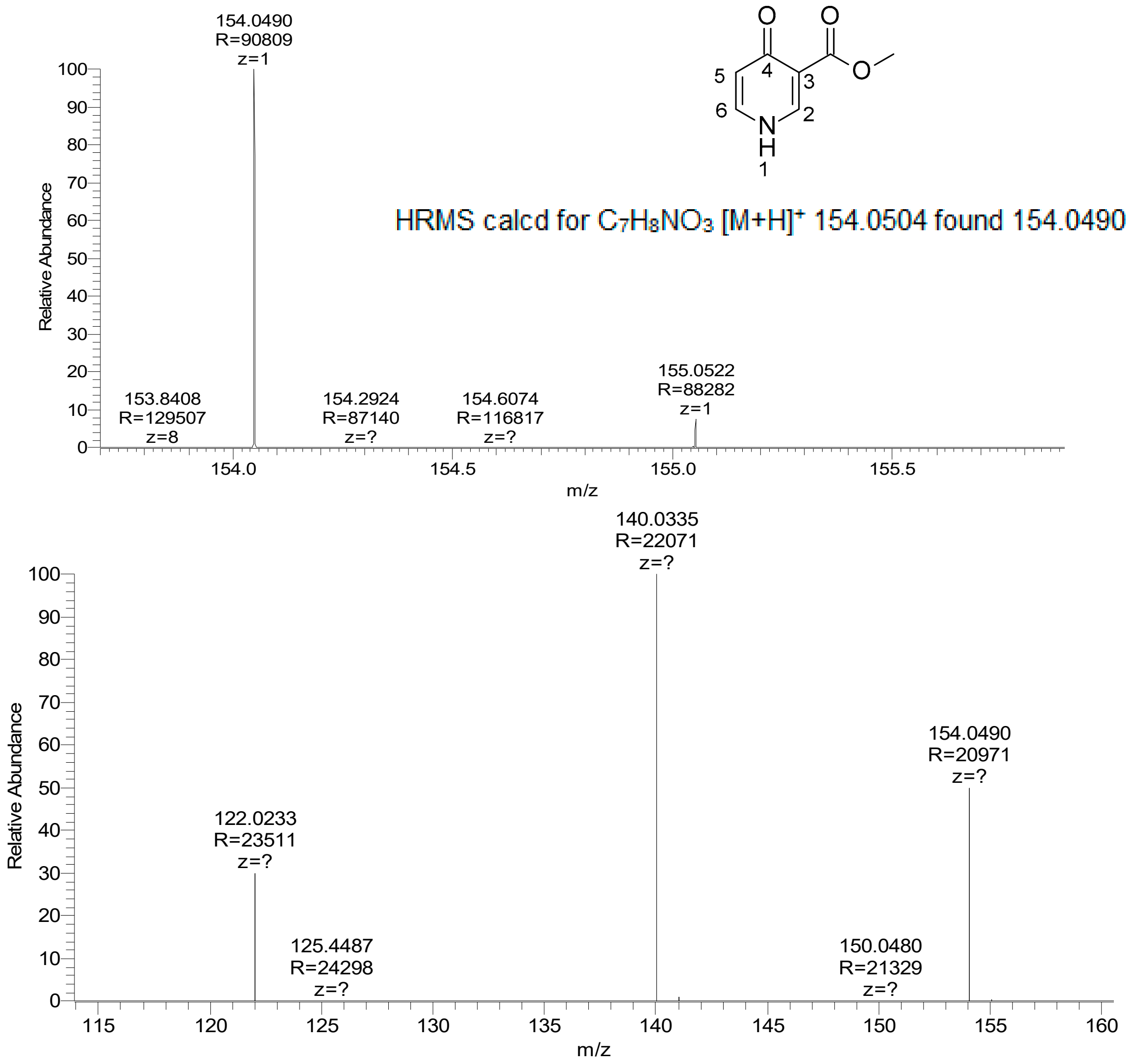

Compound 11. HRMS and MS/MS spectra

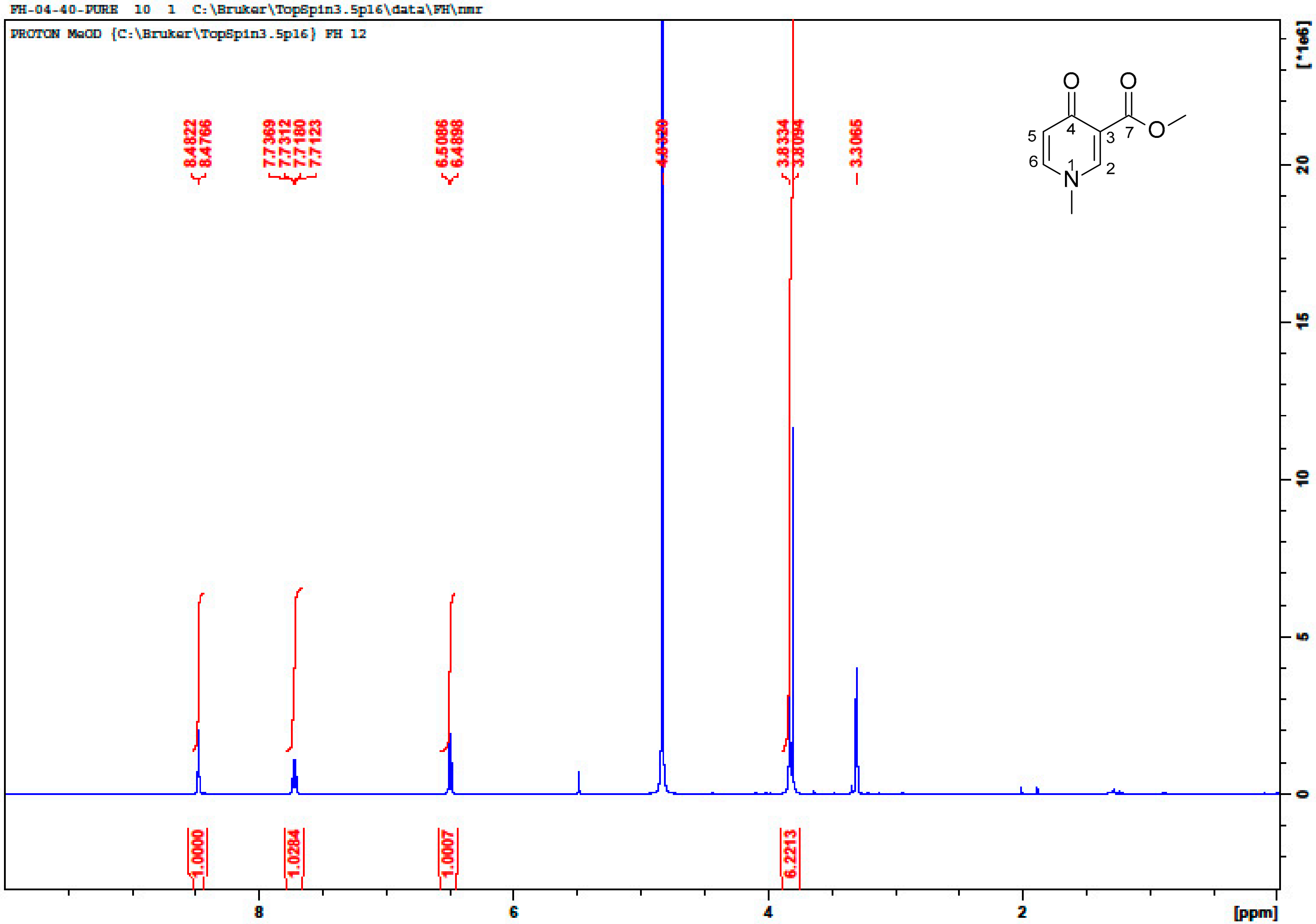

Compound 12. 400 MHz ^{1}H NMR spectrum in MeOD

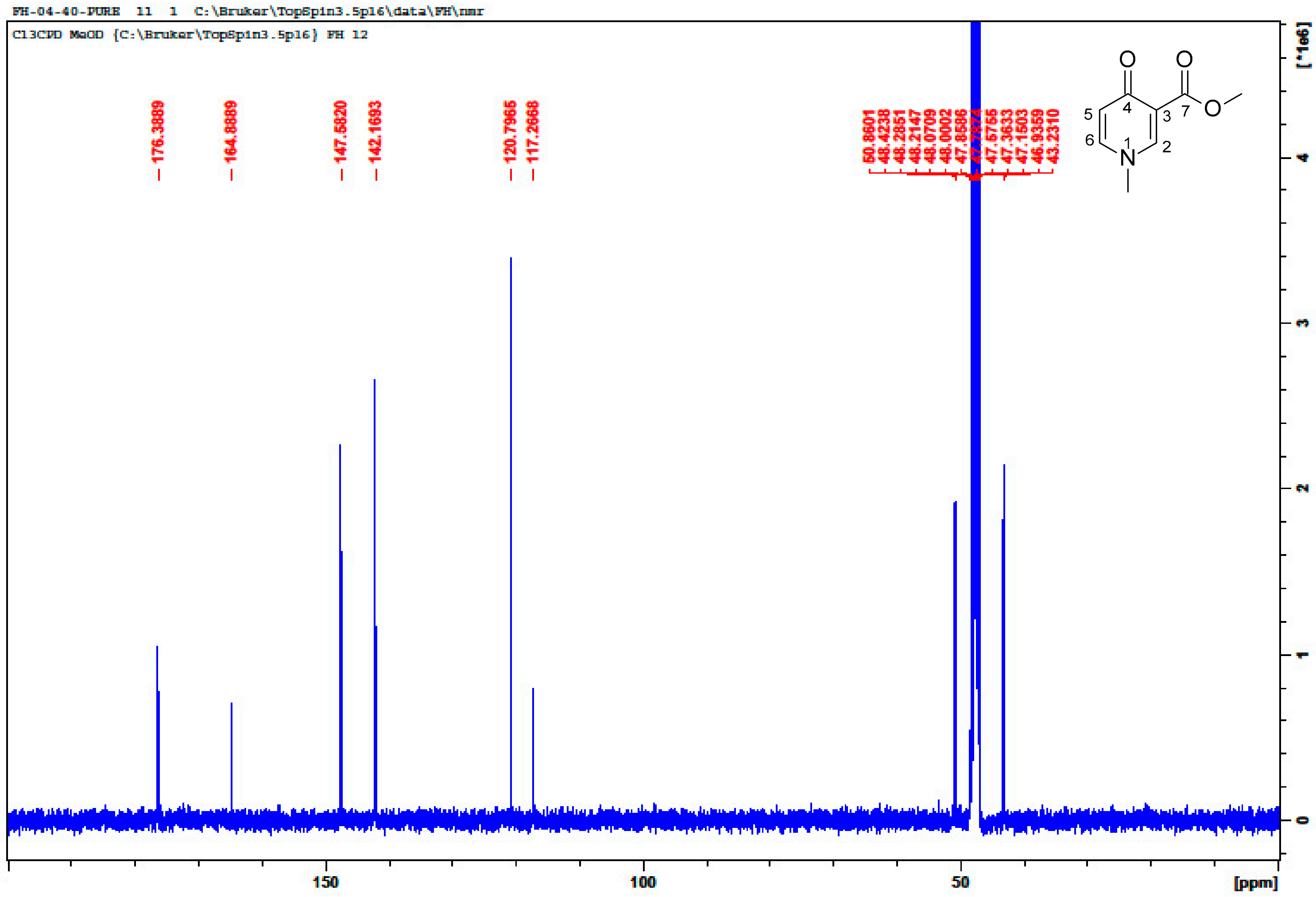

Compound 12. 400 MHz ^{13}C NMR spectrum in MeOD

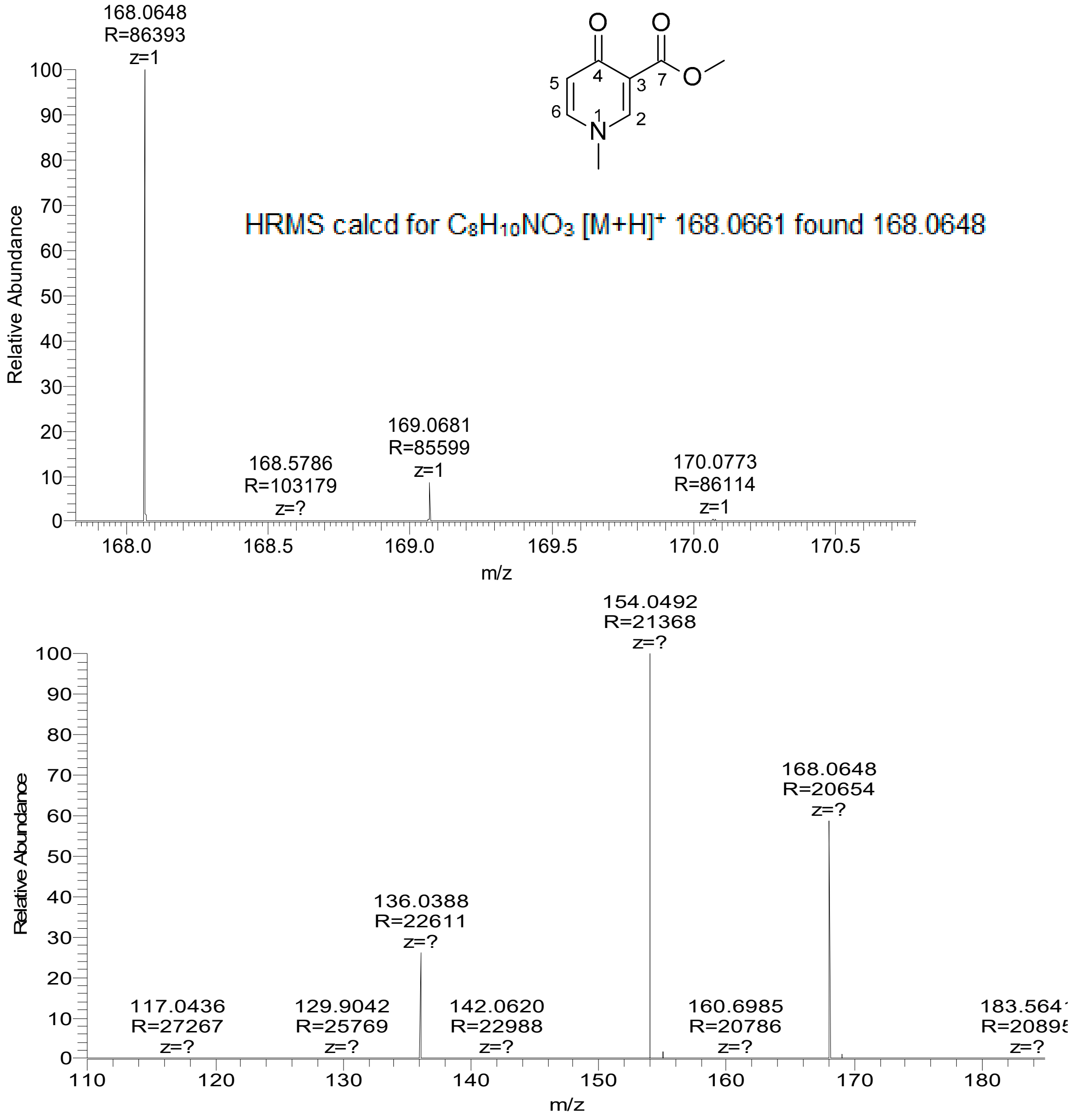

Compound 12. HRMS and MS/MS spectra

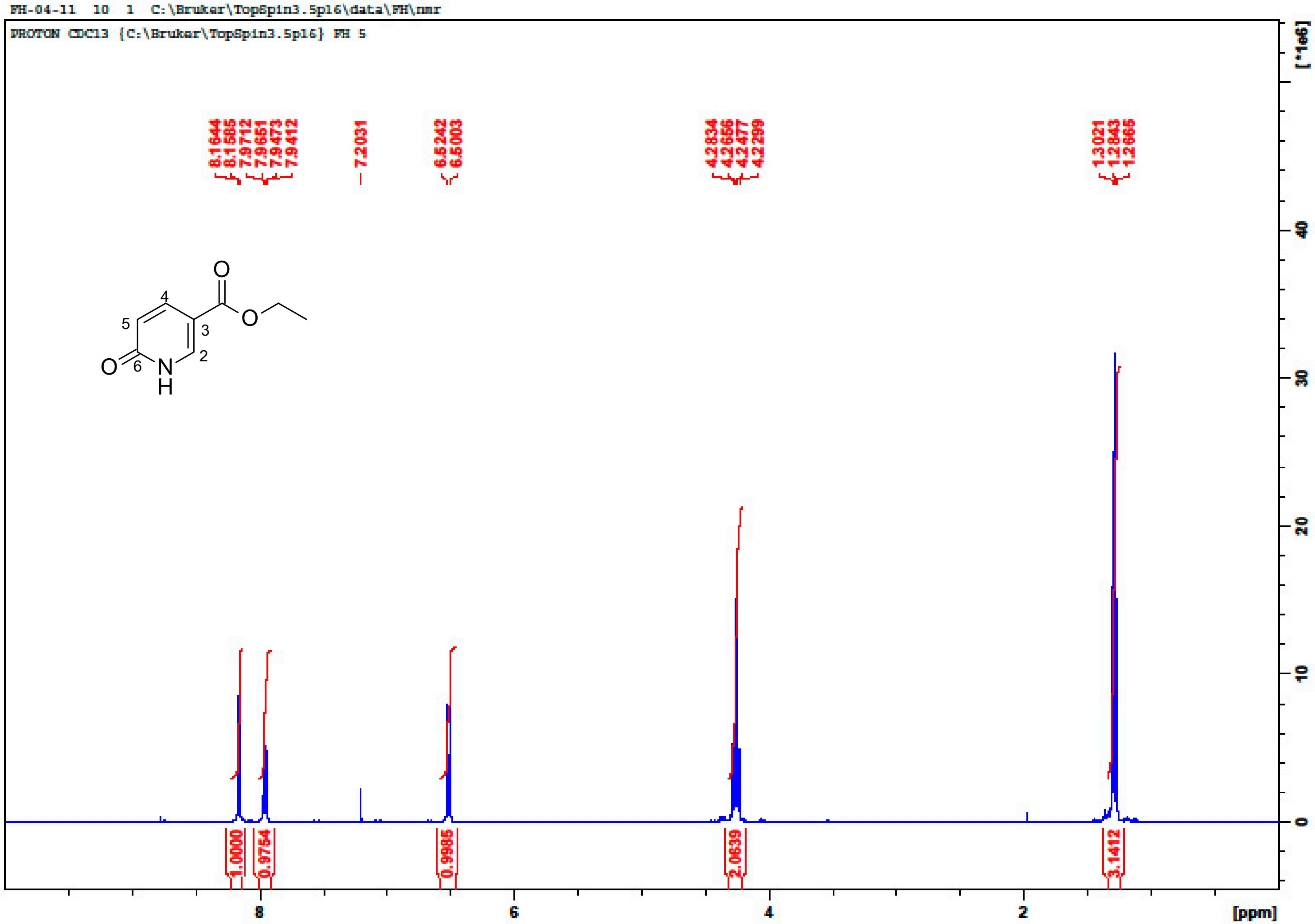

Compound 14. 400 MHz ^{1}H NMR spectrum in $CDCl_3$

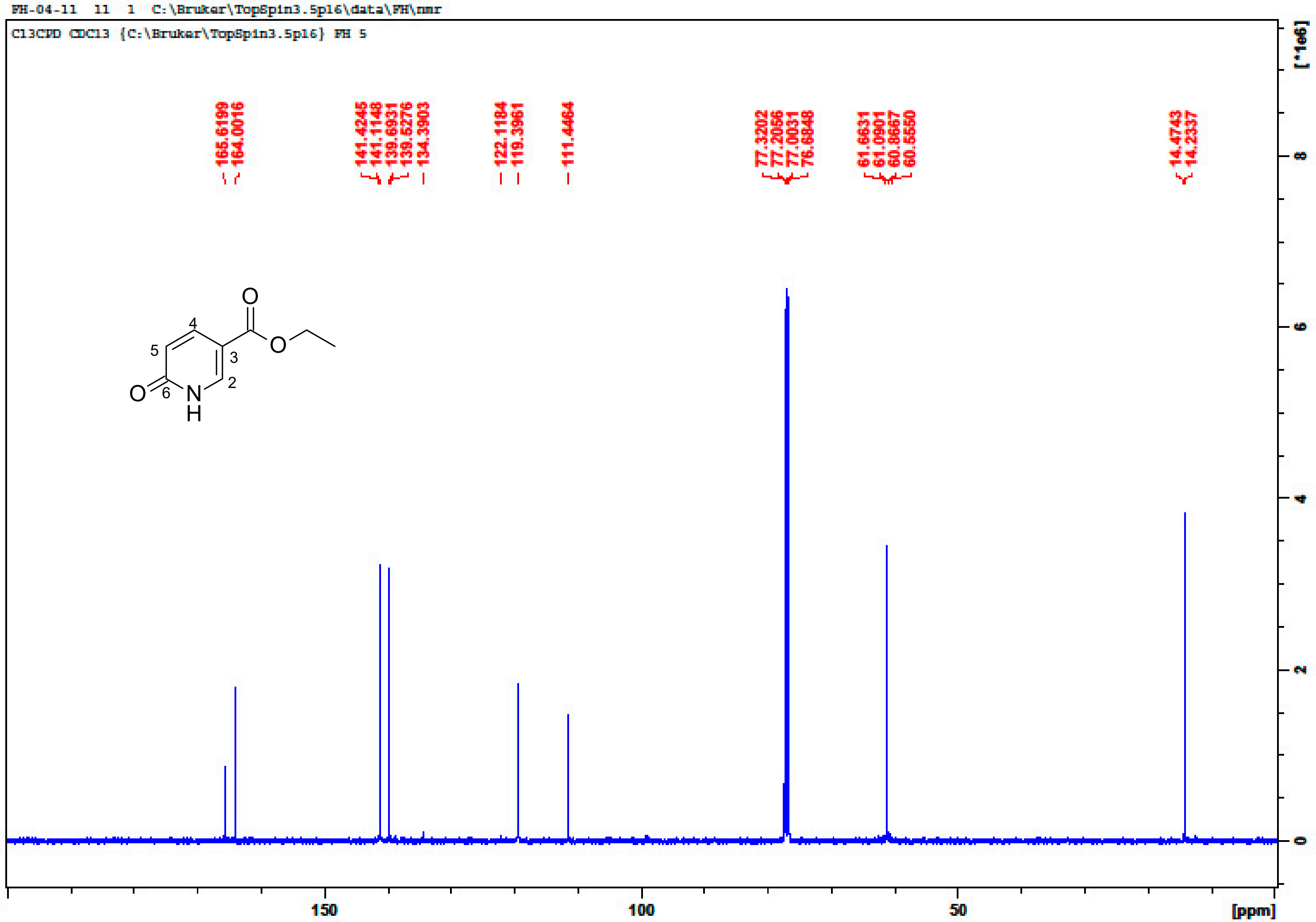

Compound 14. 400 MHz ^{13}C NMR spectrum in $CDCl_3$

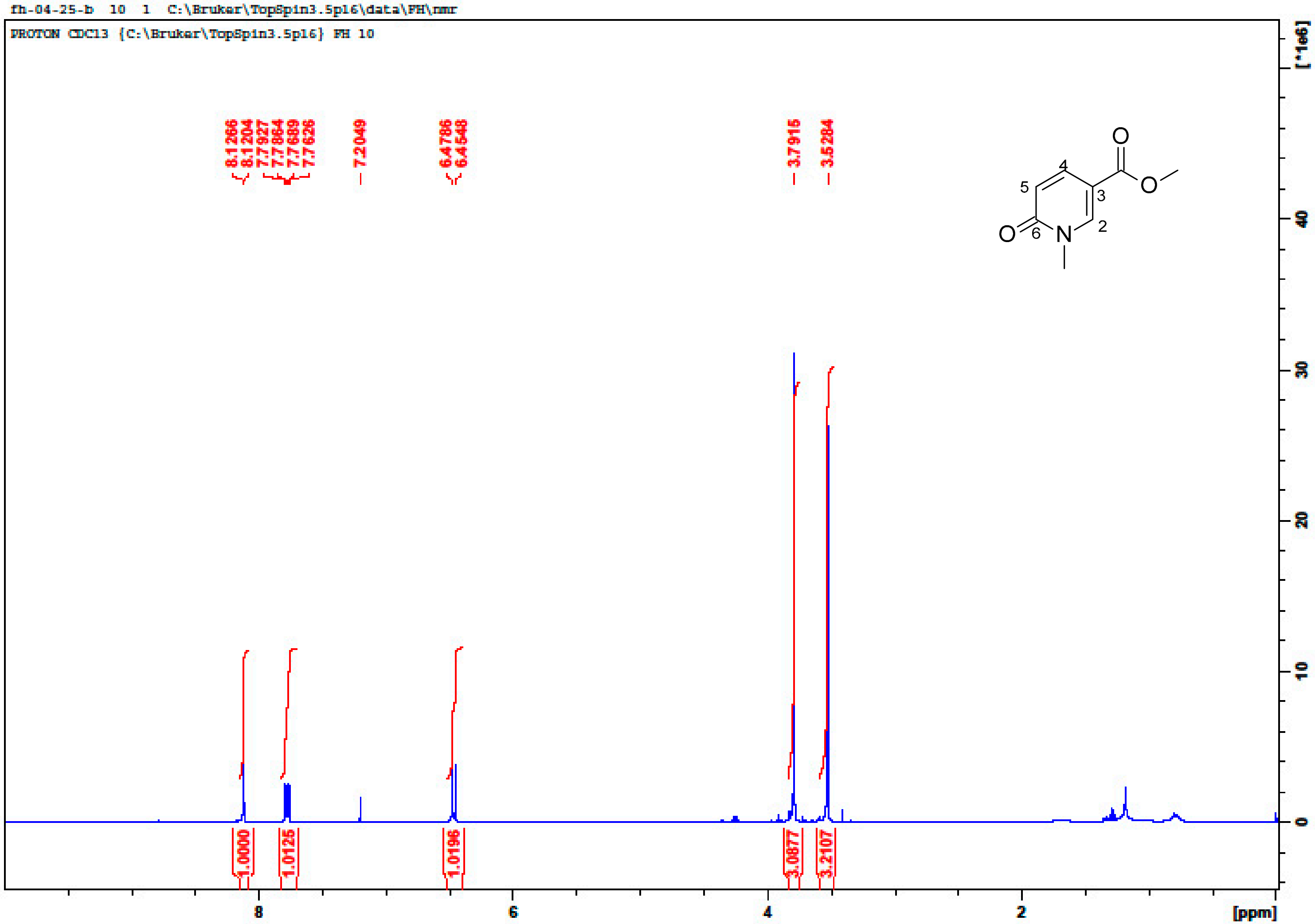

Compound 15. 400 MHz ^{1}H NMR spectrum in $CDCl_3$

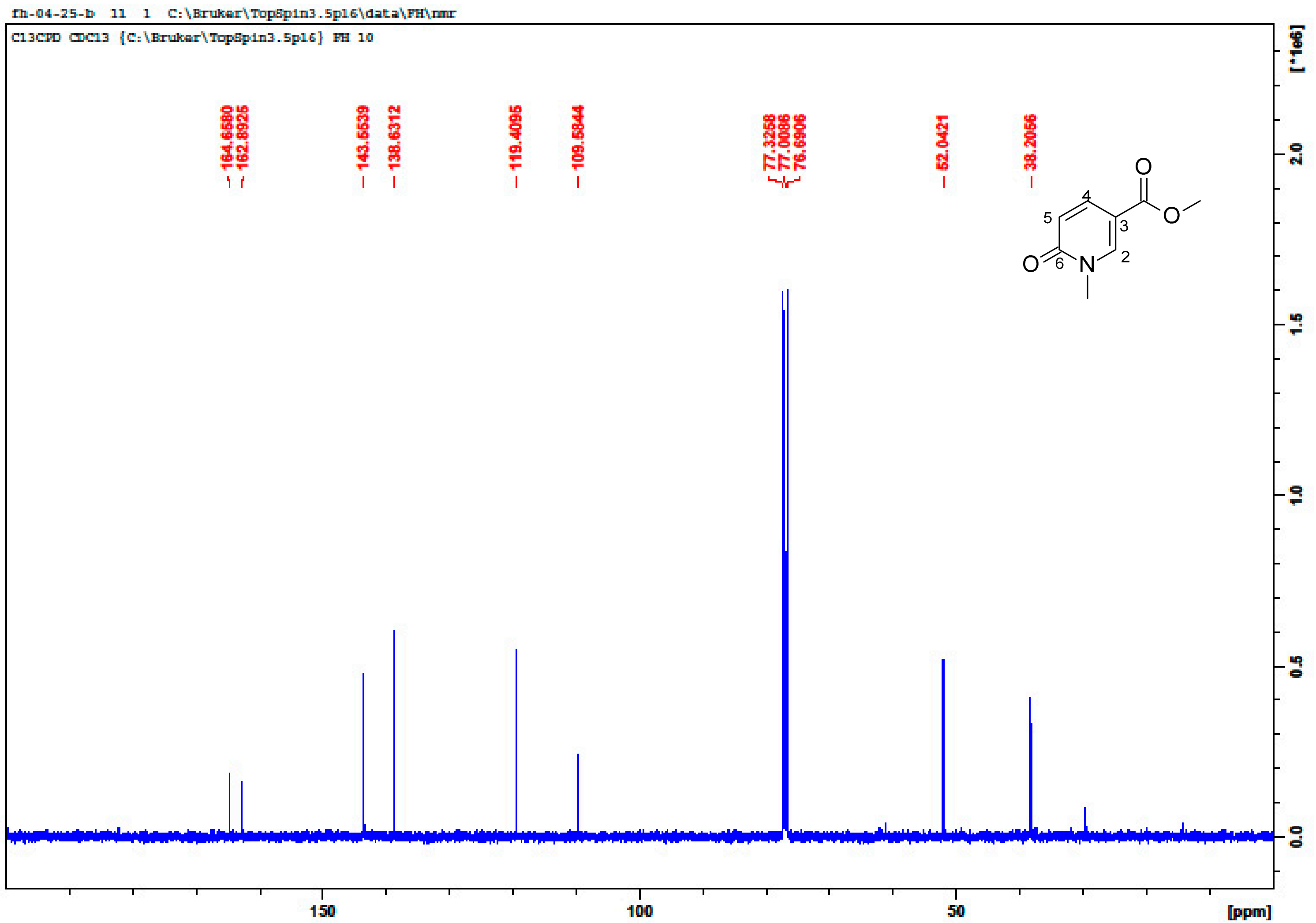

Compound 15. 400 MHz ^{13}C NMR spectrum in $CDCl_3$

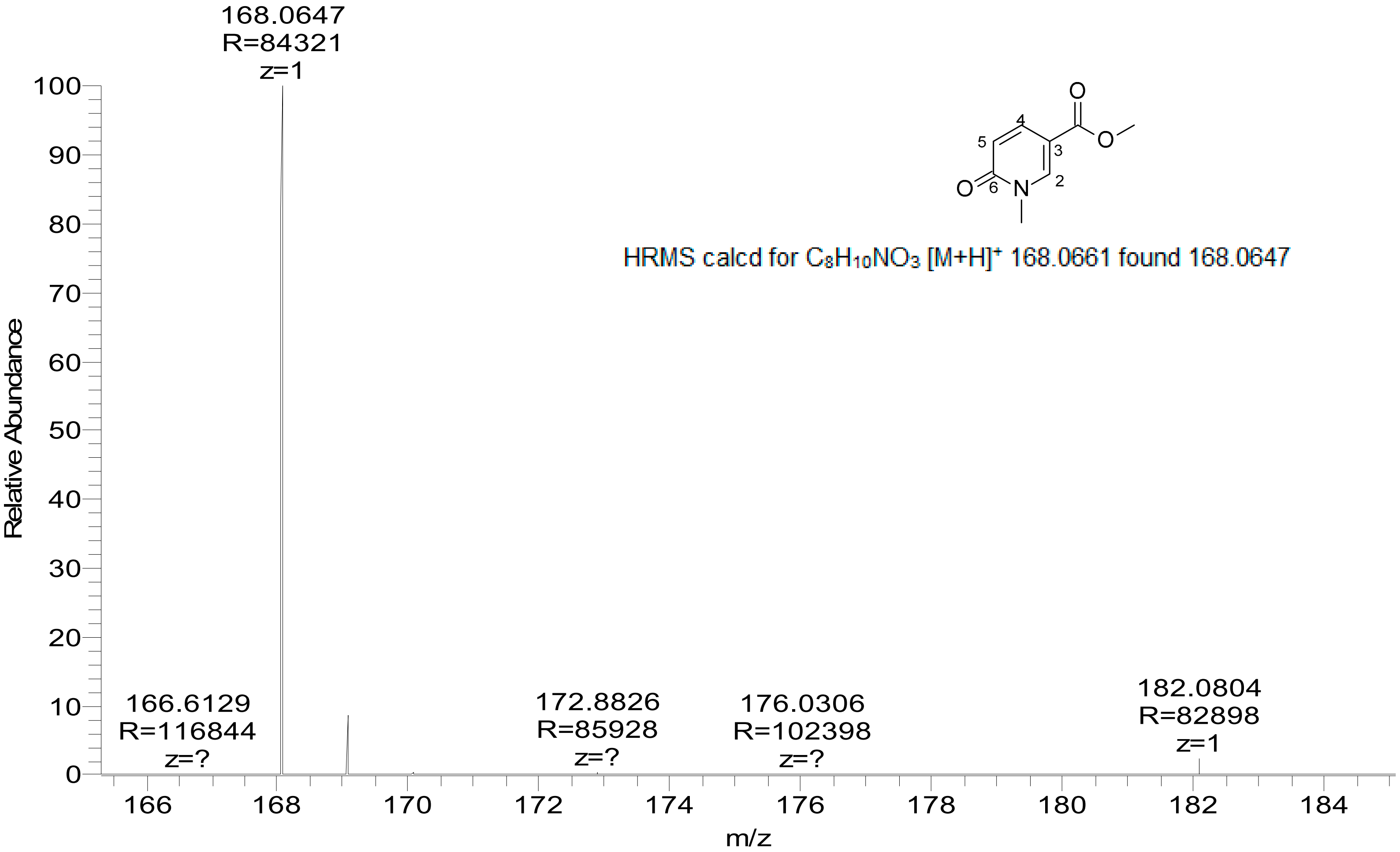

Compound 15. HRMS spectra

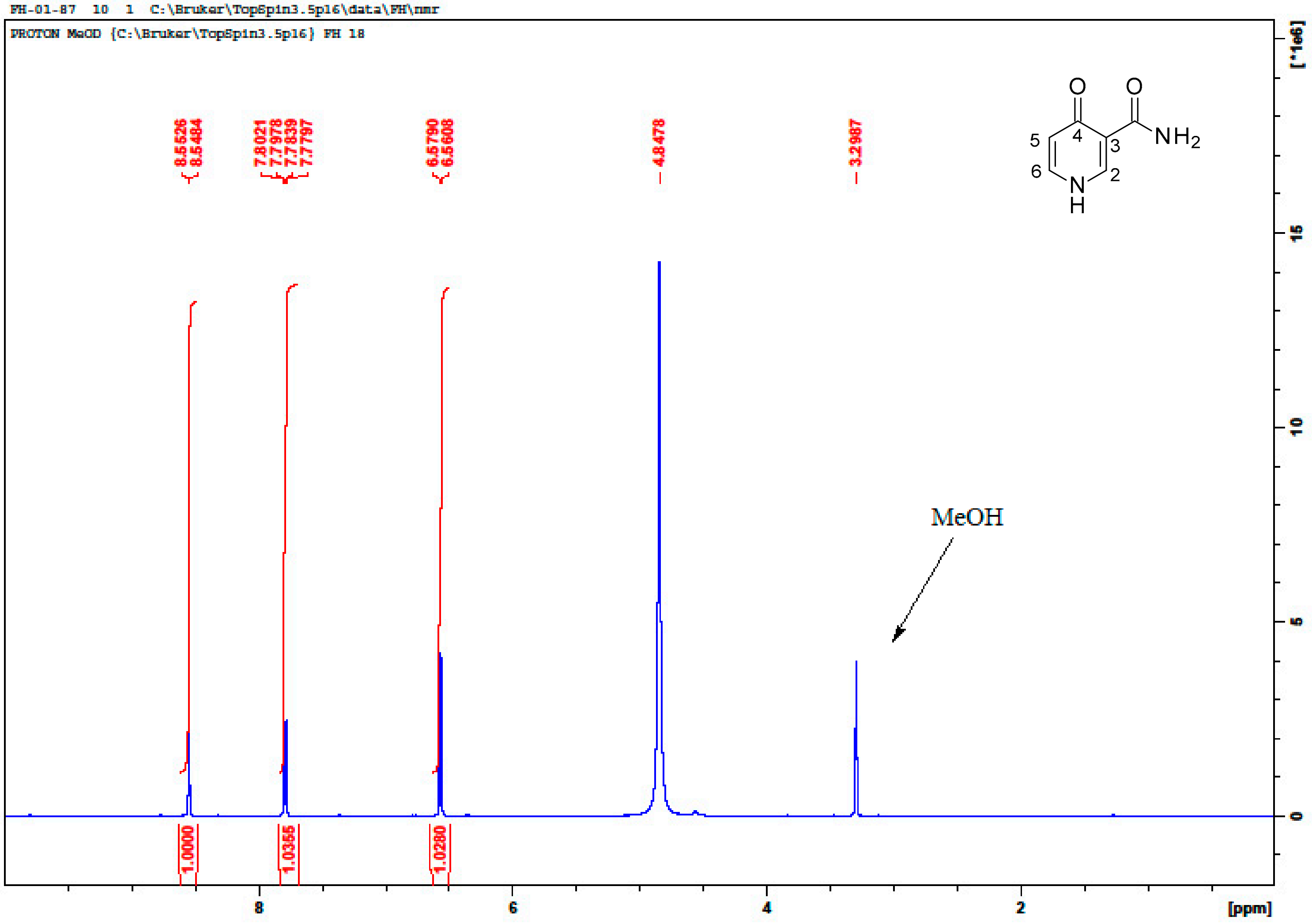

Compound 16. 400 MHz ^{1}H NMR spectrum in MeOD

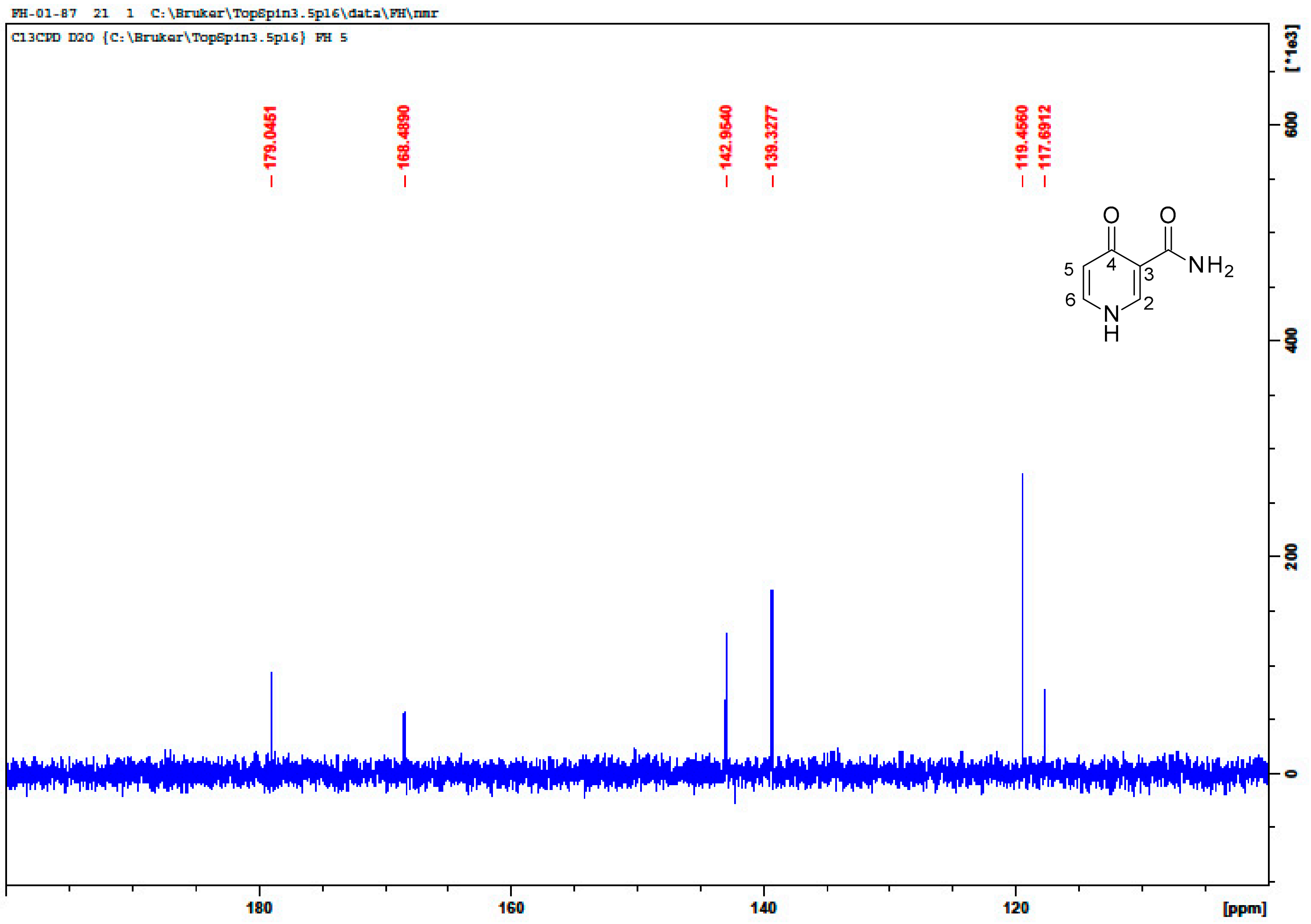

Compound 16. 100 MHz ^{13}C NMR spectrum in MeOD

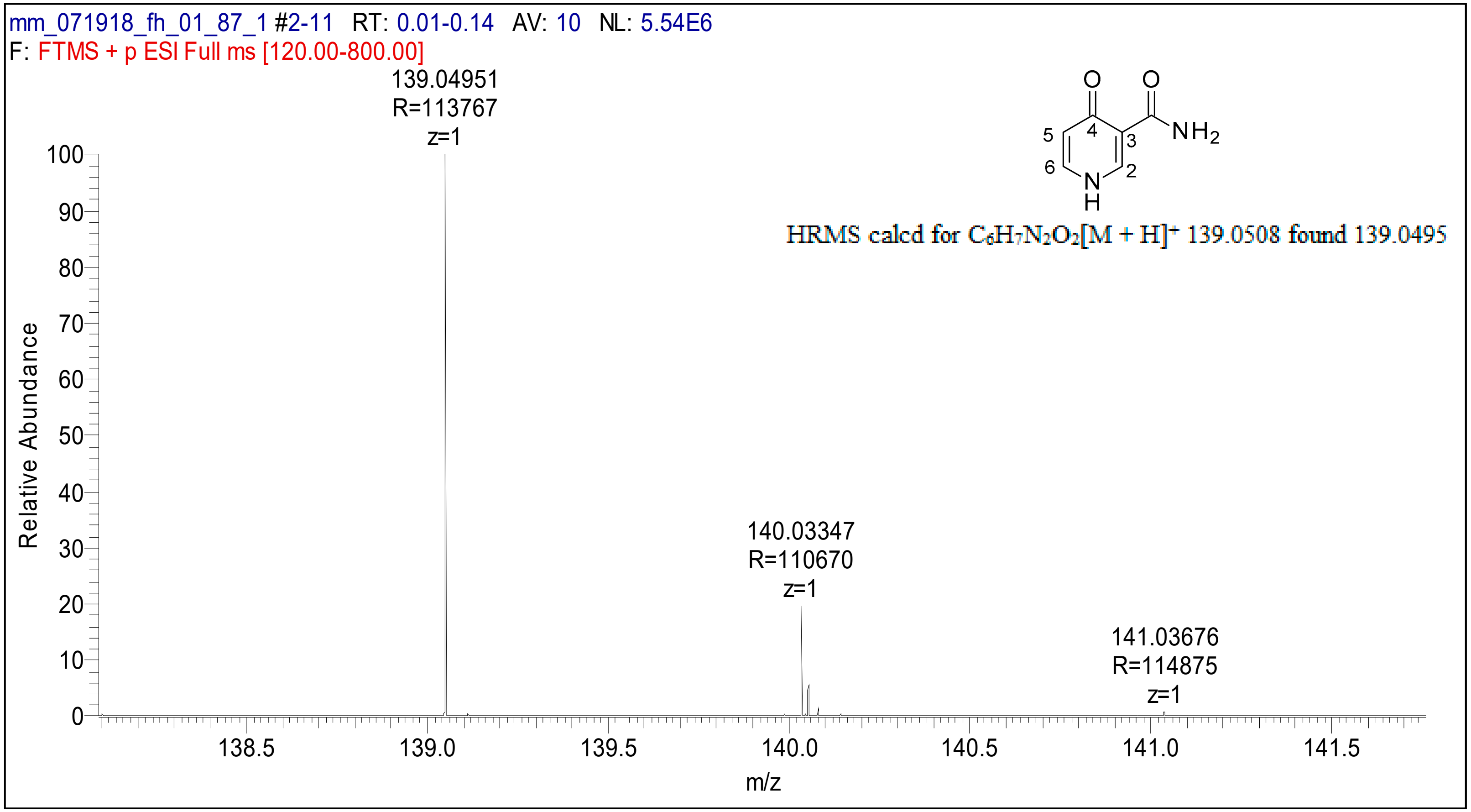

Compound 16. HRMS spectra

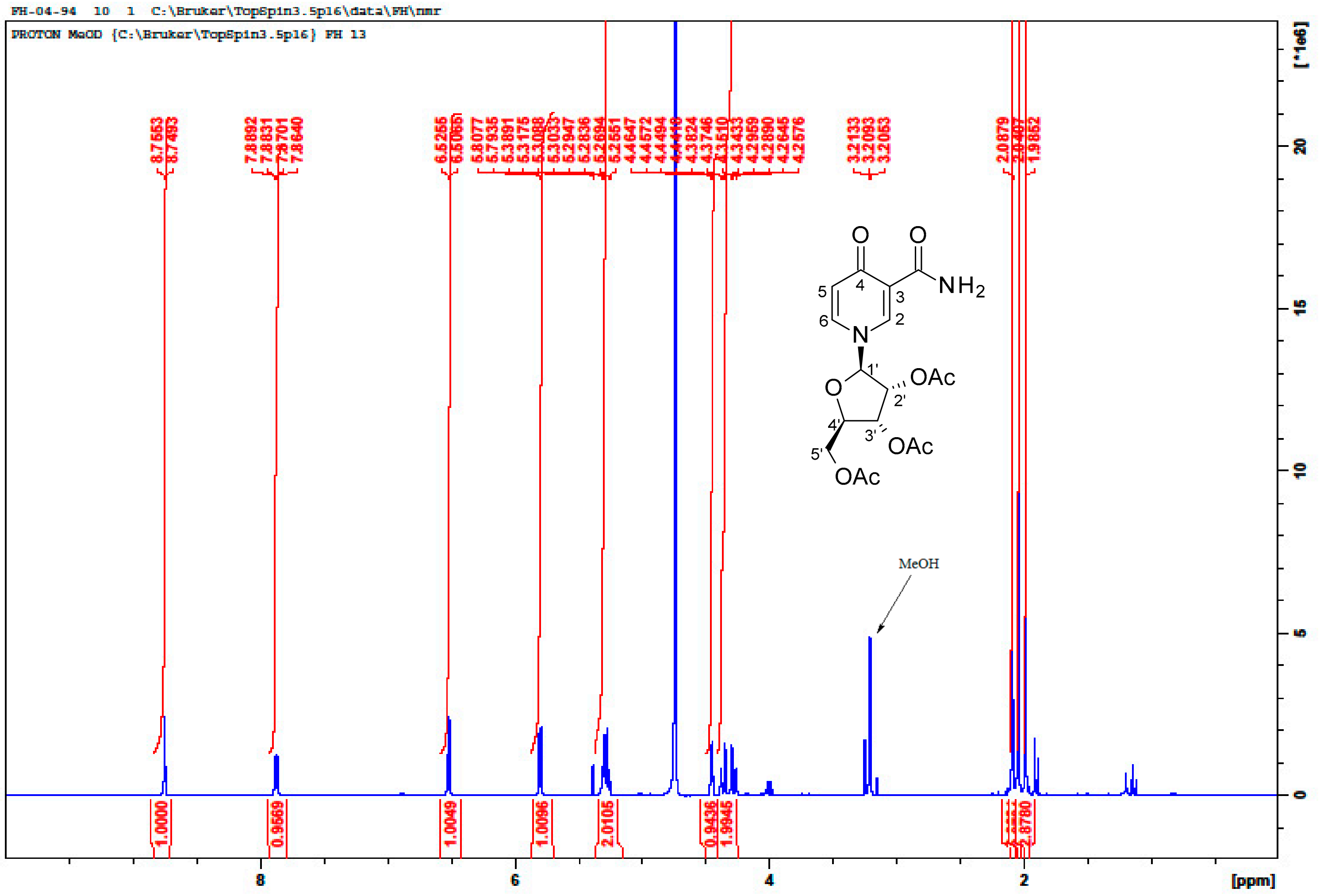

Compound 18. 400 MHz ^{1}H NMR spectrum in MeOD

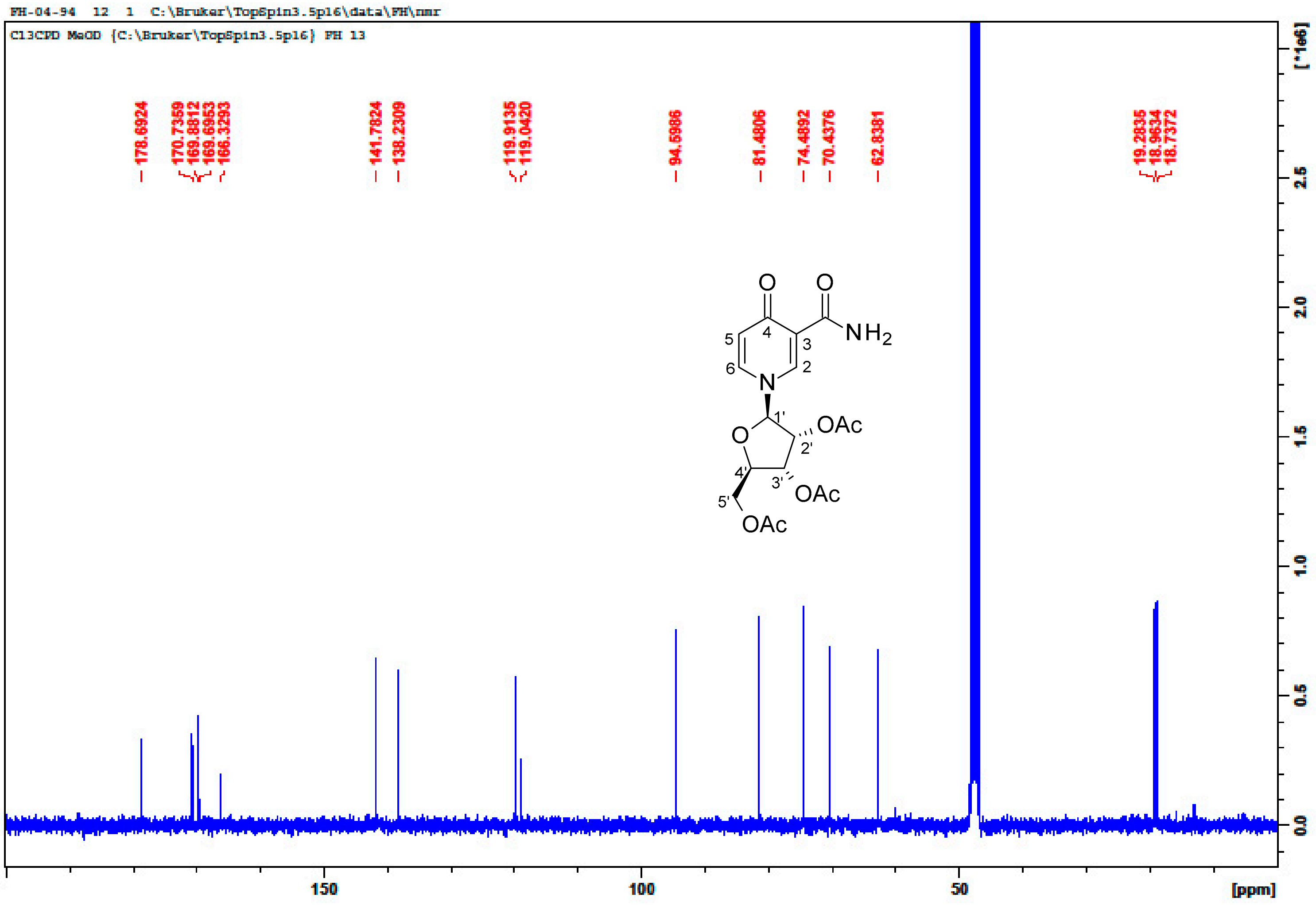

Compound 18. 100 MHz ^{13}C NMR spectrum in MeOD

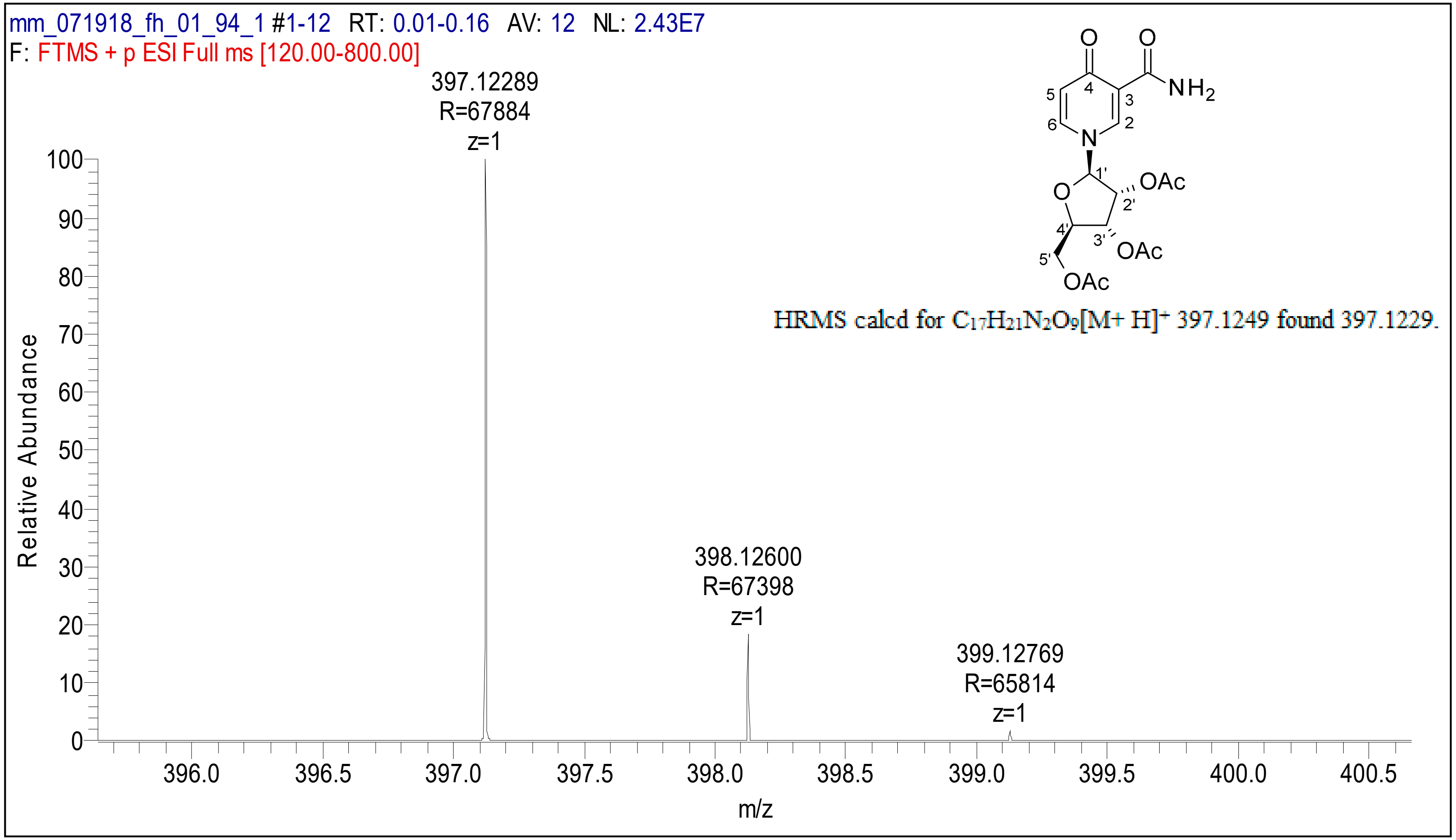

Compound 18. HRMS spectra

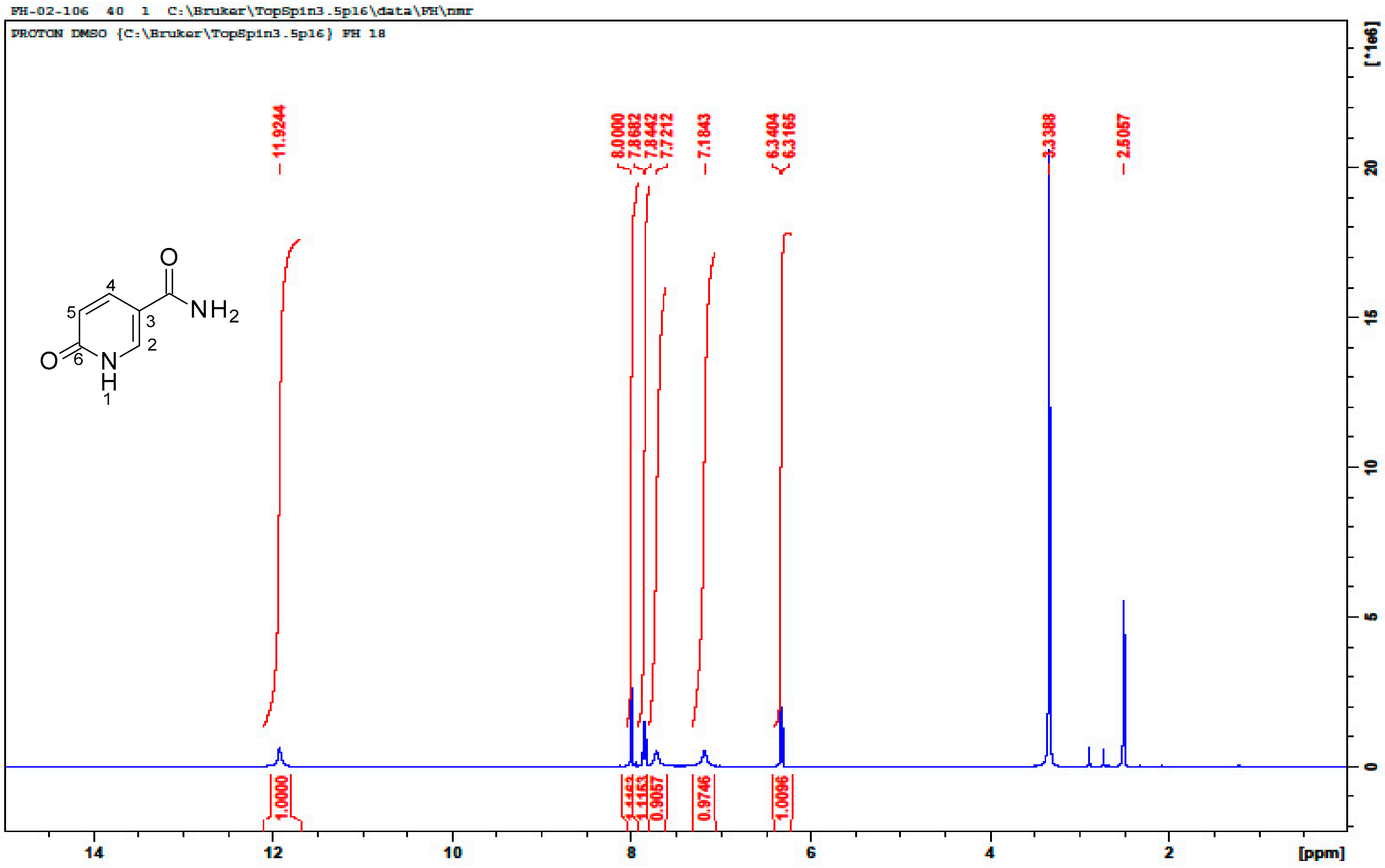

Compound 20. 400 MHz ^{1}H NMR spectrum in DMSO

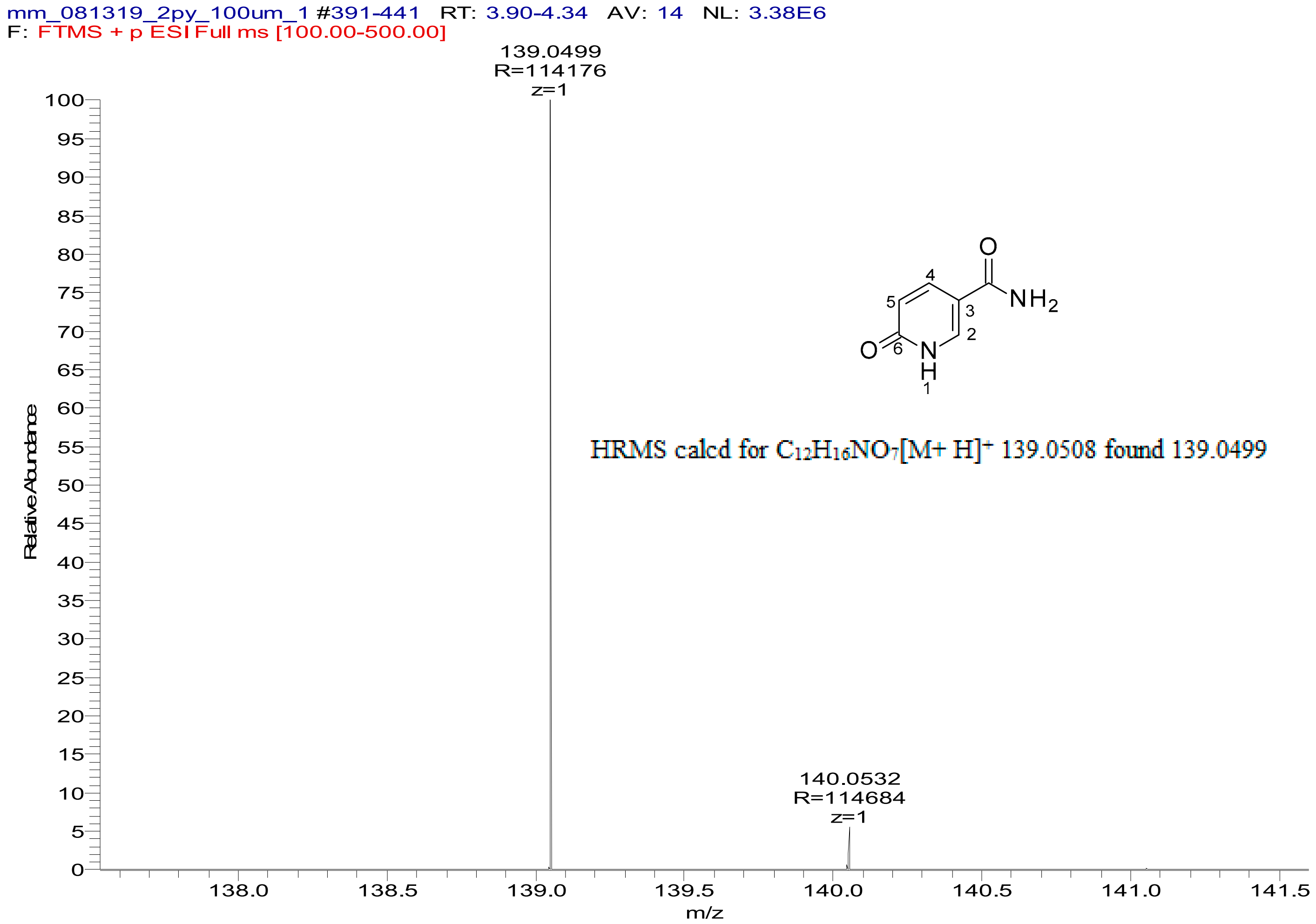

Compound 20. HRMS spectra

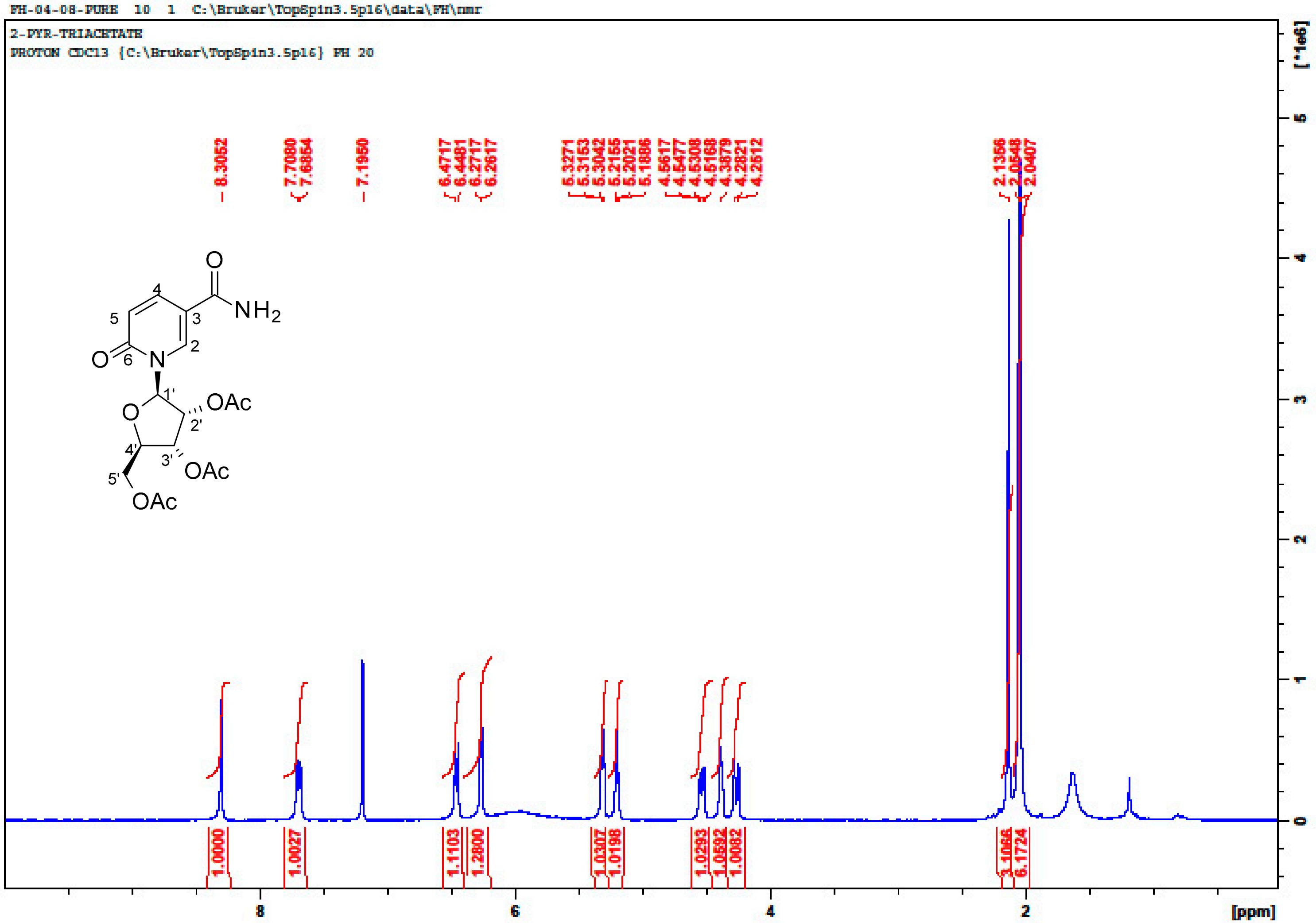

Compound 22. 400 MHz ^{1}H NMR spectrum in $CDCl_3$

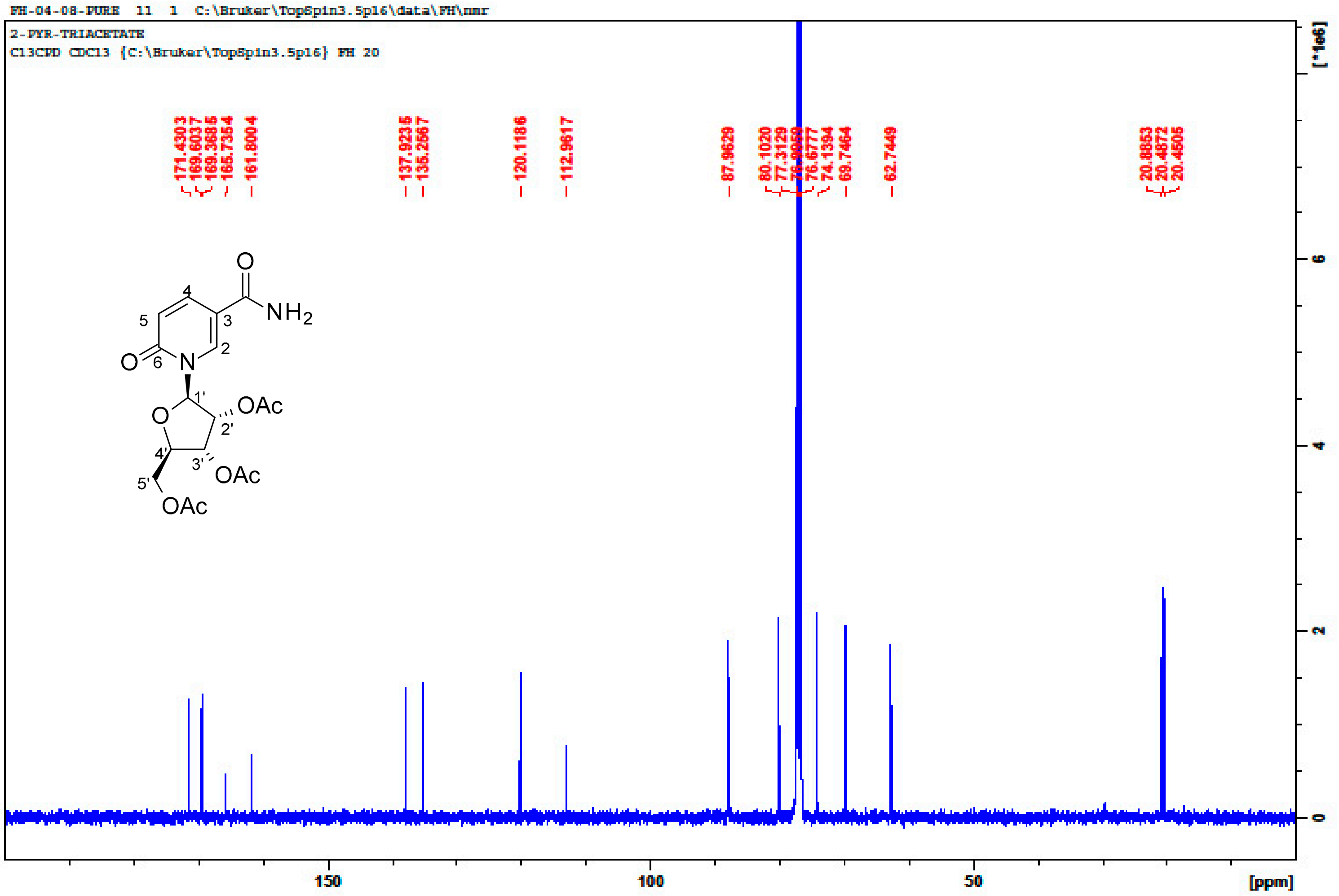

Compound 22. 400 MHz ^{13}C NMR spectrum in $CDCl_3$

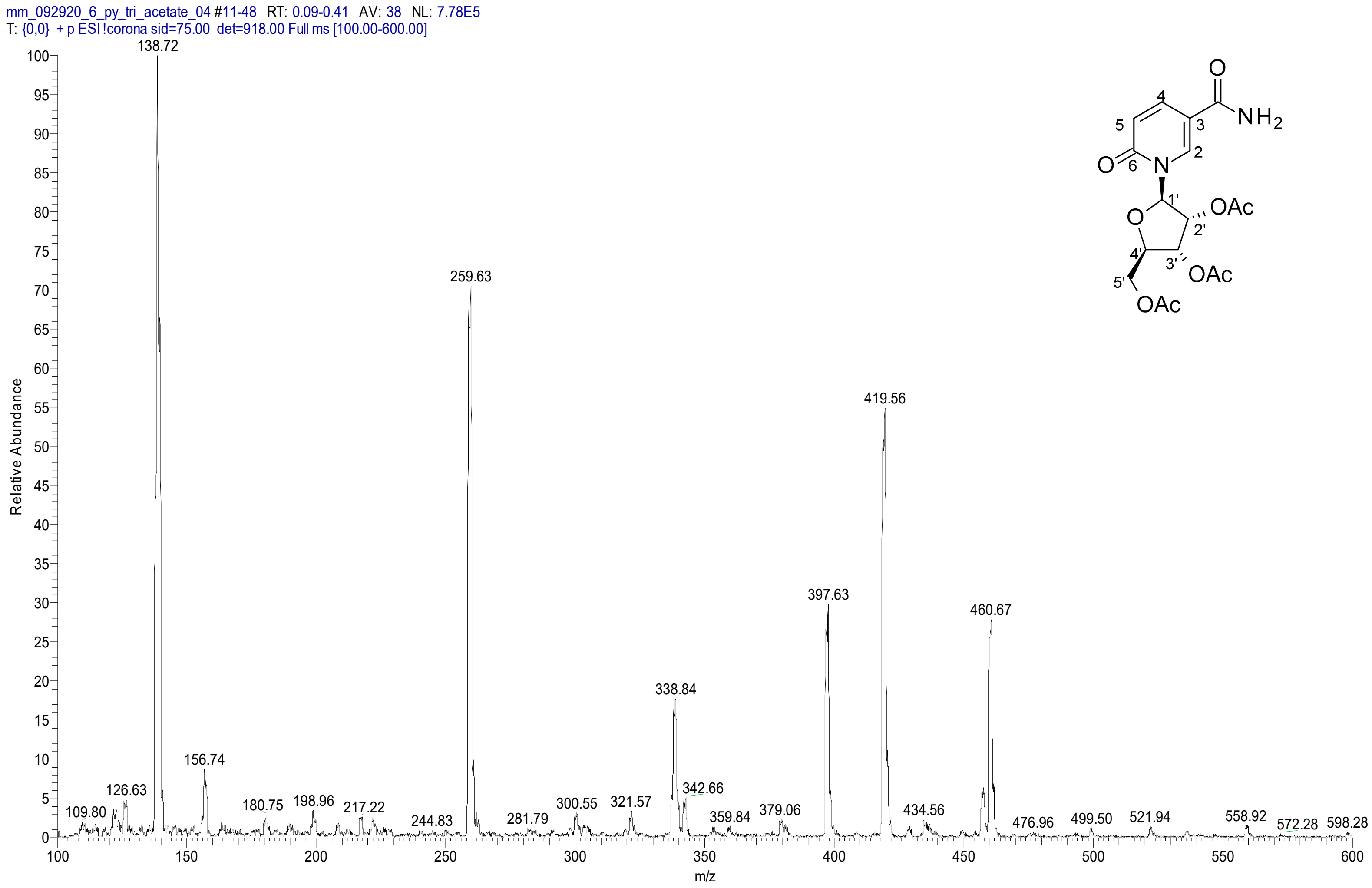

Compound 22. (ES)MS spectra

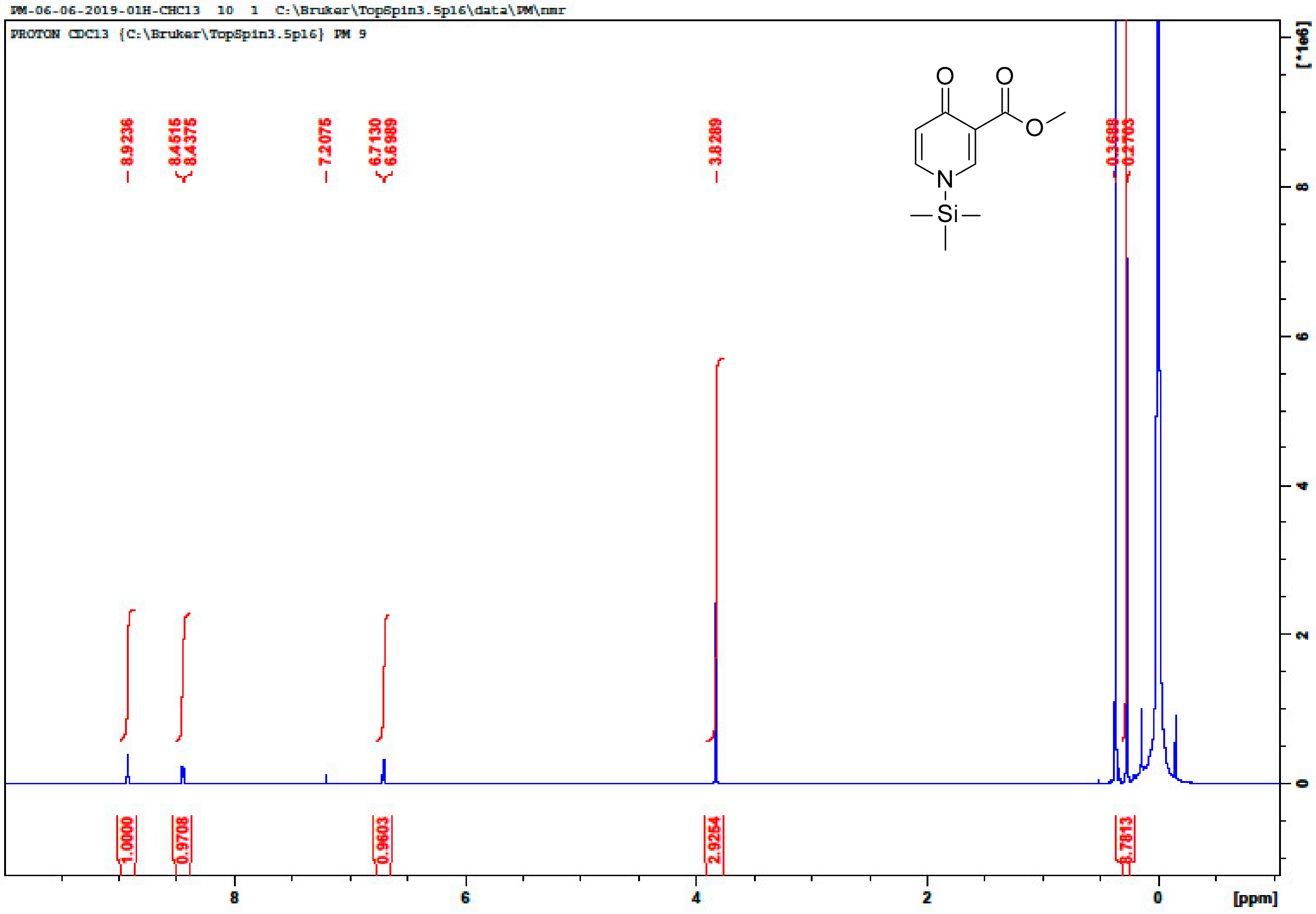

Compound 23. 400 MHz ^{1}H NMR spectrum in $CDCl_3$

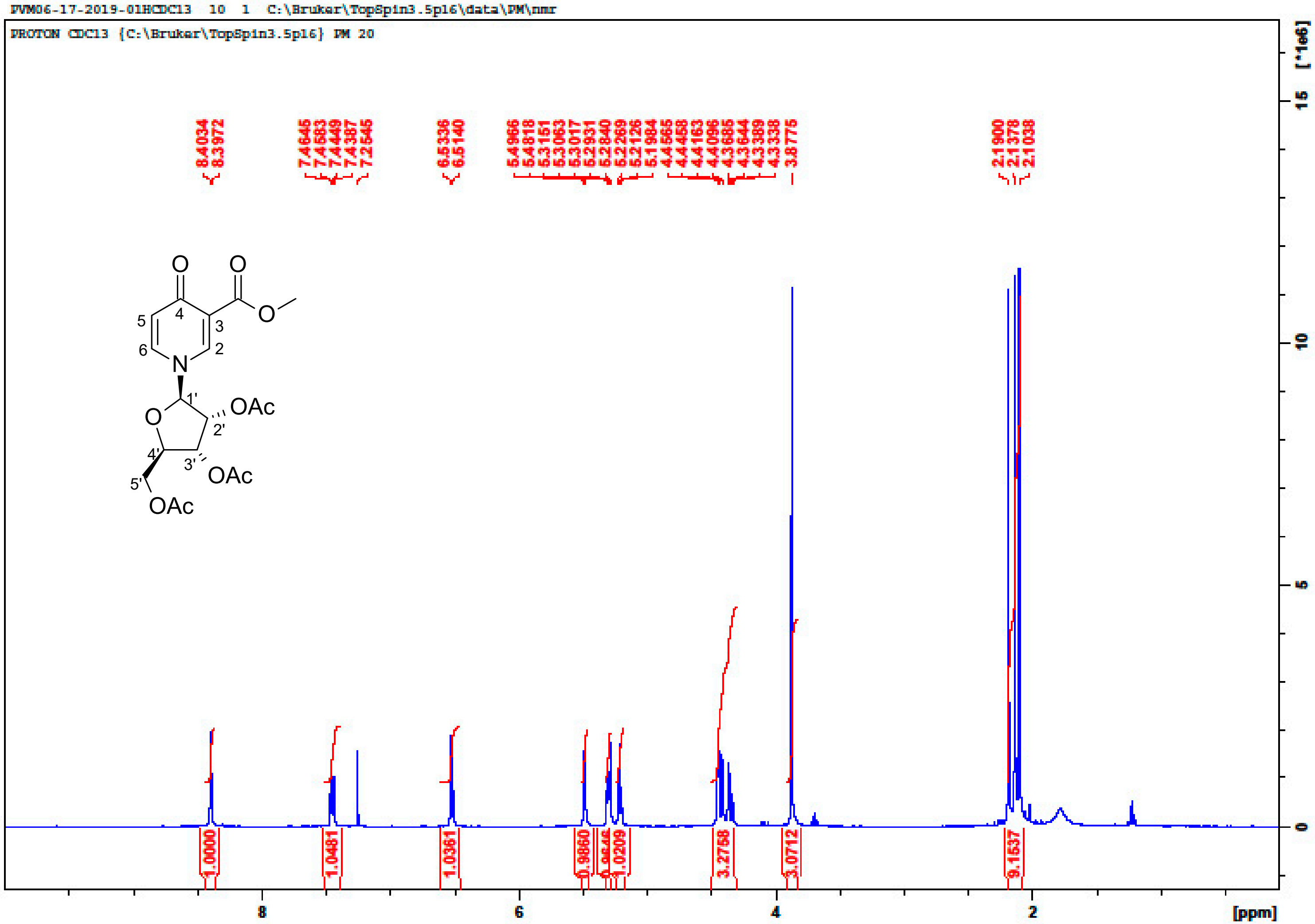

Compound 24. 400 MHz ^{1}H NMR spectrum in $CDCl_3$

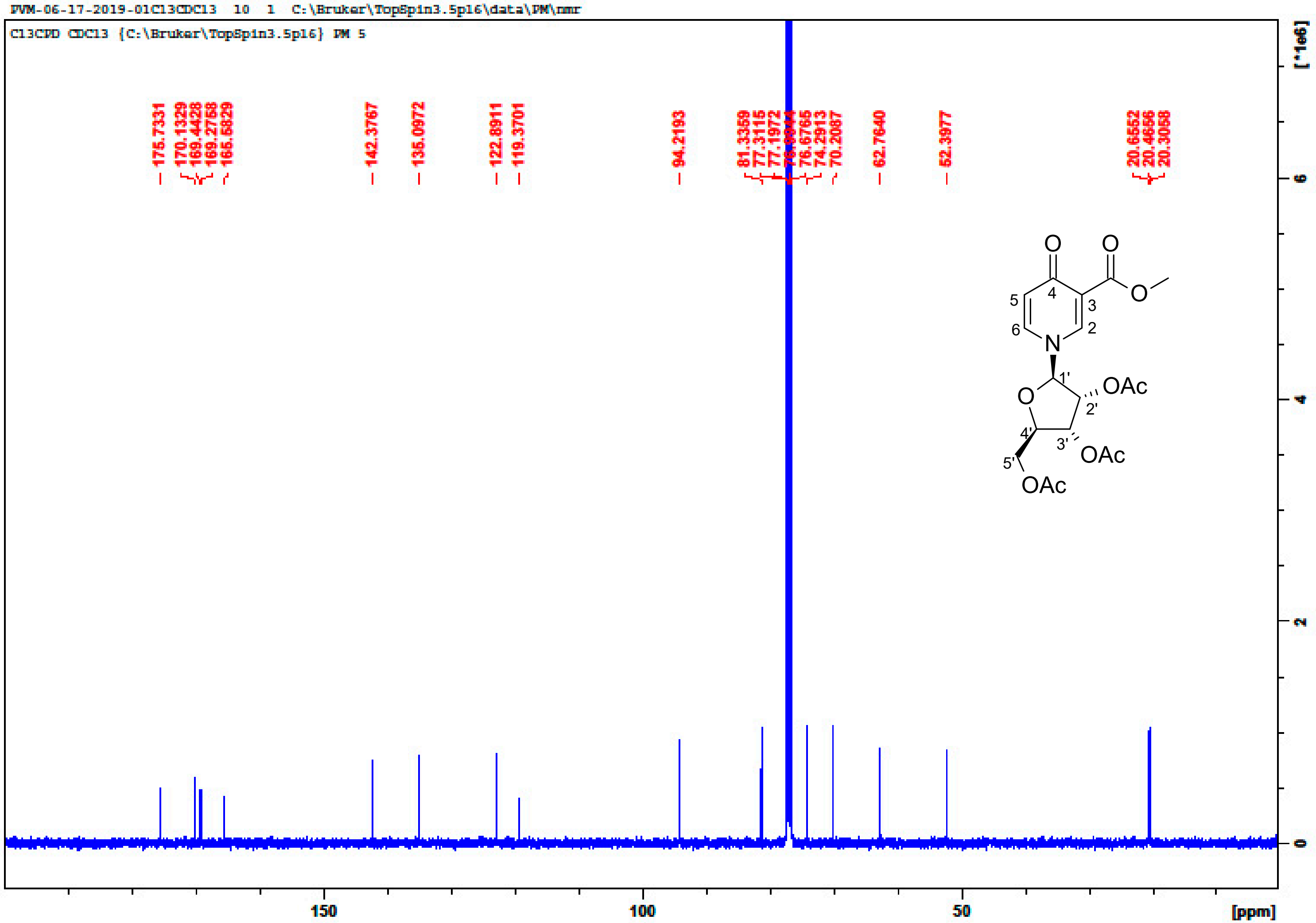

Compound 24. 400 MHz ^{13}C NMR spectrum in $CDCl_3$

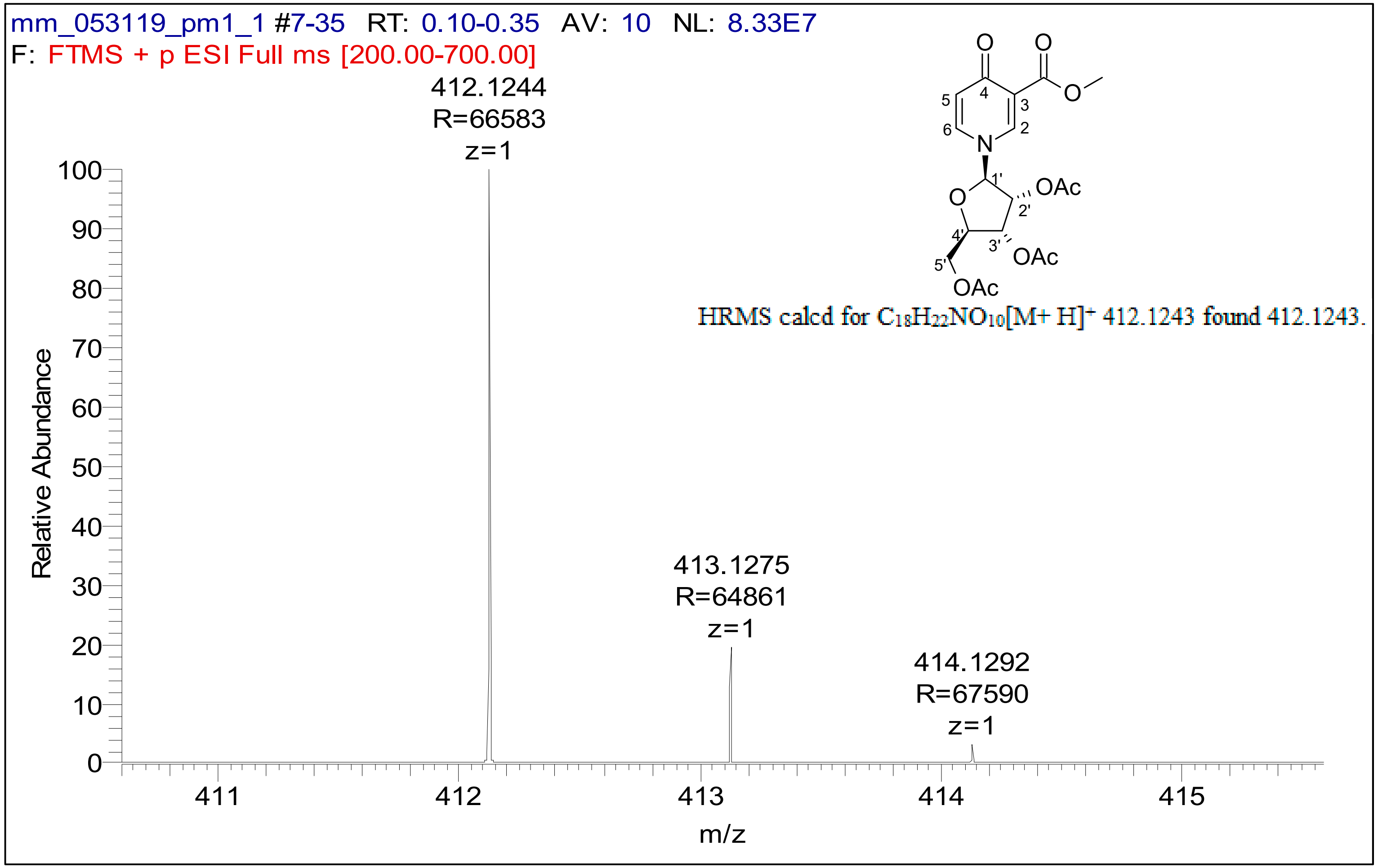

Compound 24. HRMS spectra

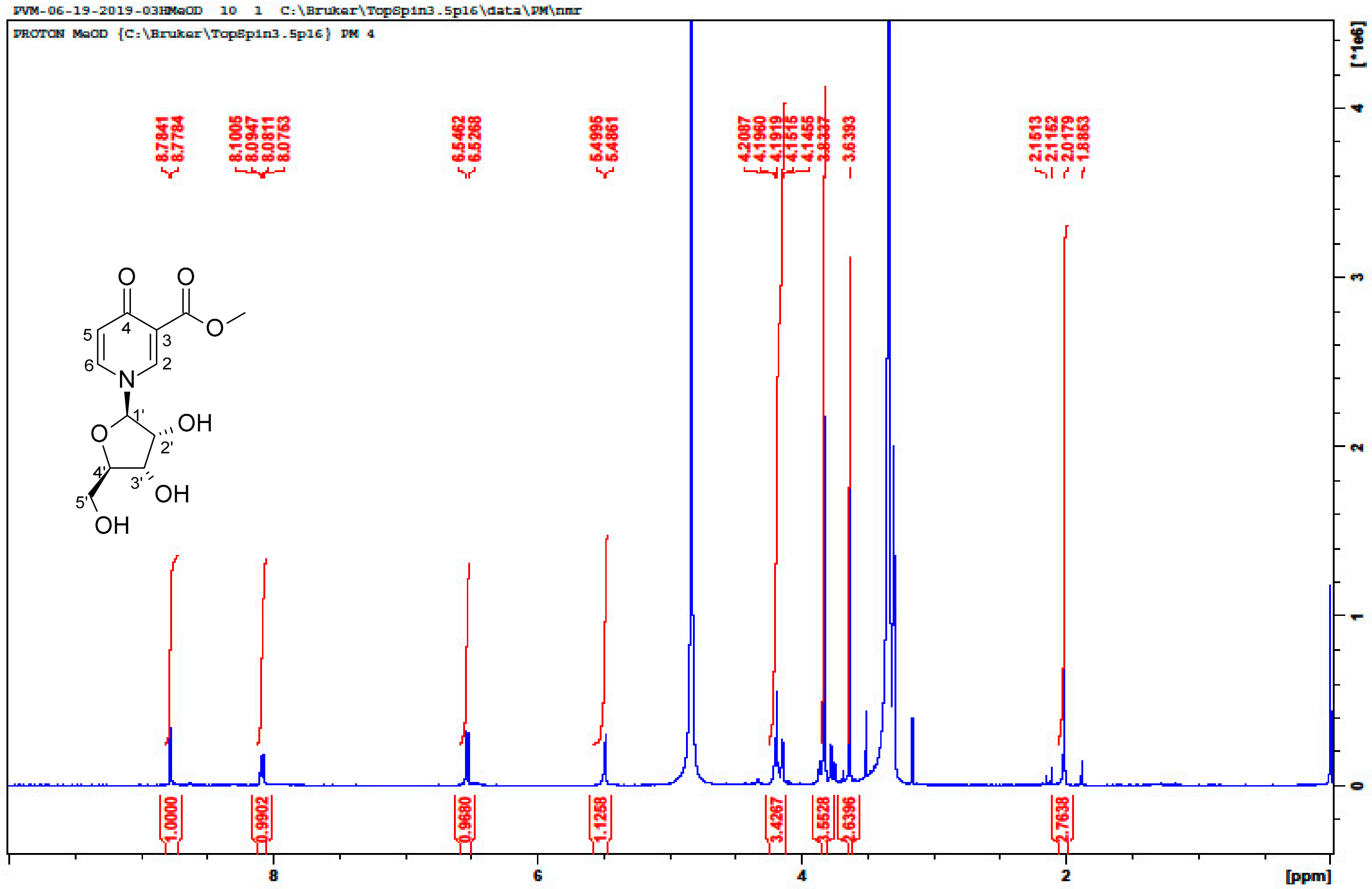

Compound 25. 400 MHz ^{1}H NMR spectrum in MeOD

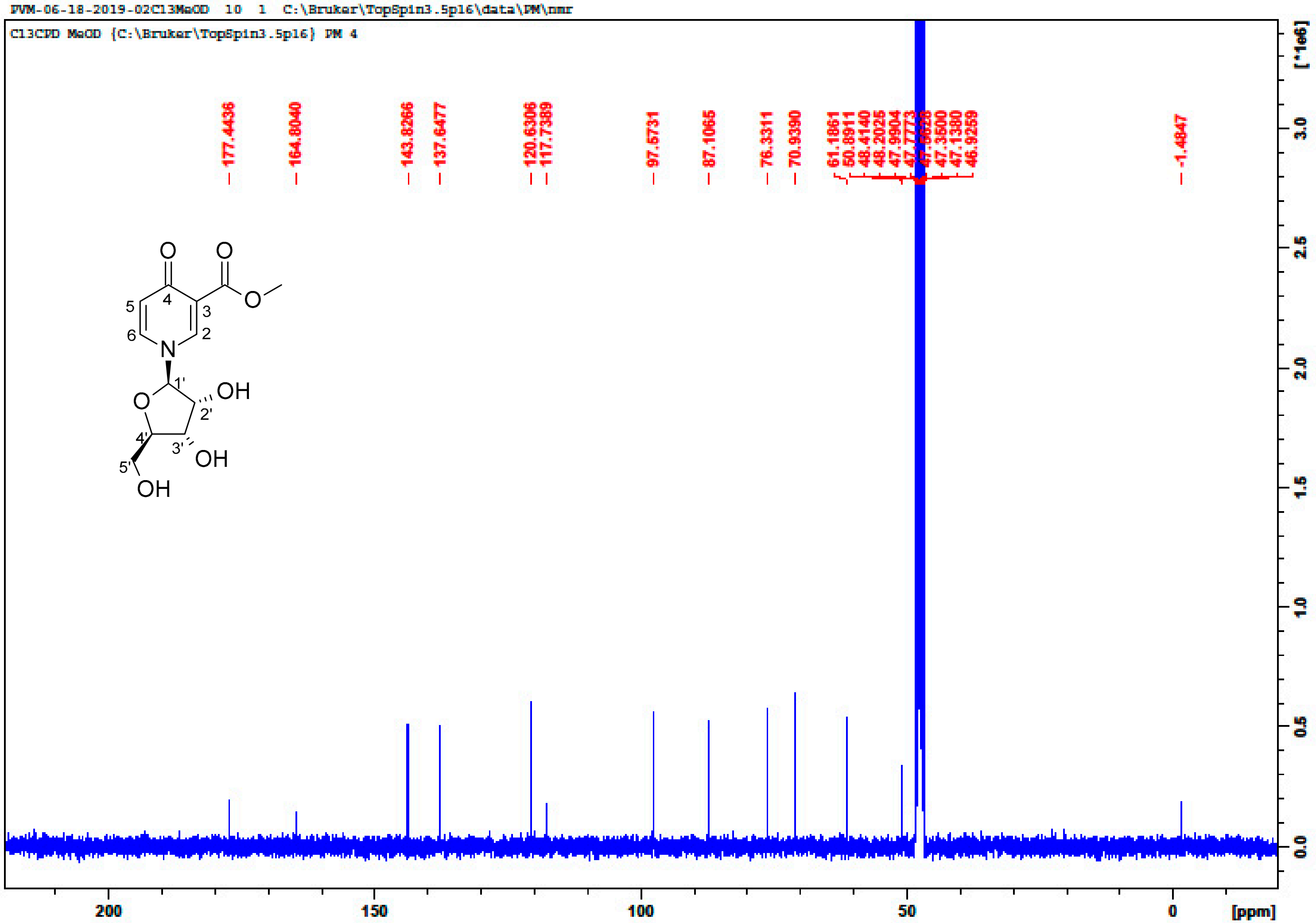

Compound 25. 400 MHz ^{13}C NMR spectrum in MeOD

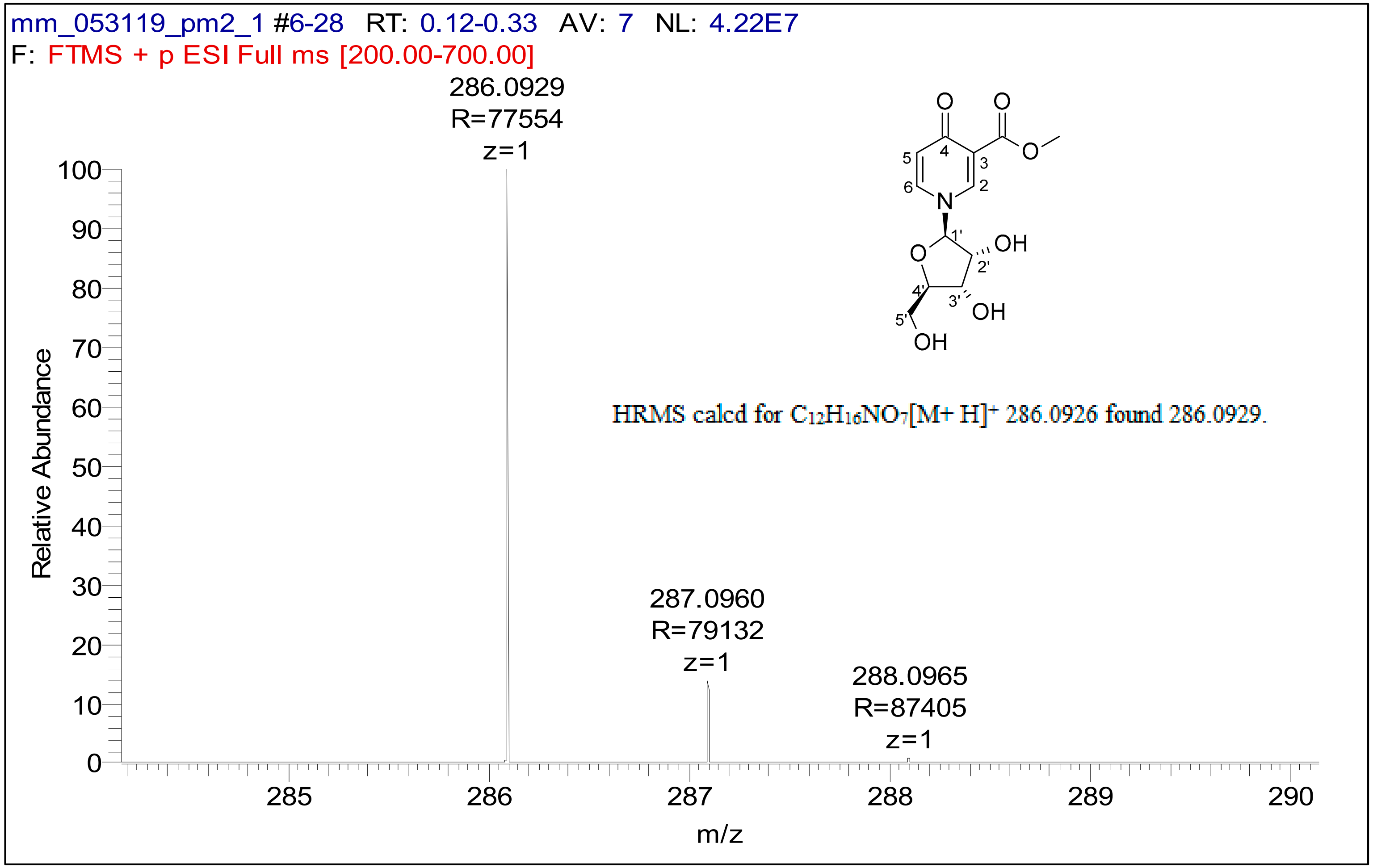

Compound 25. HRMS spectra

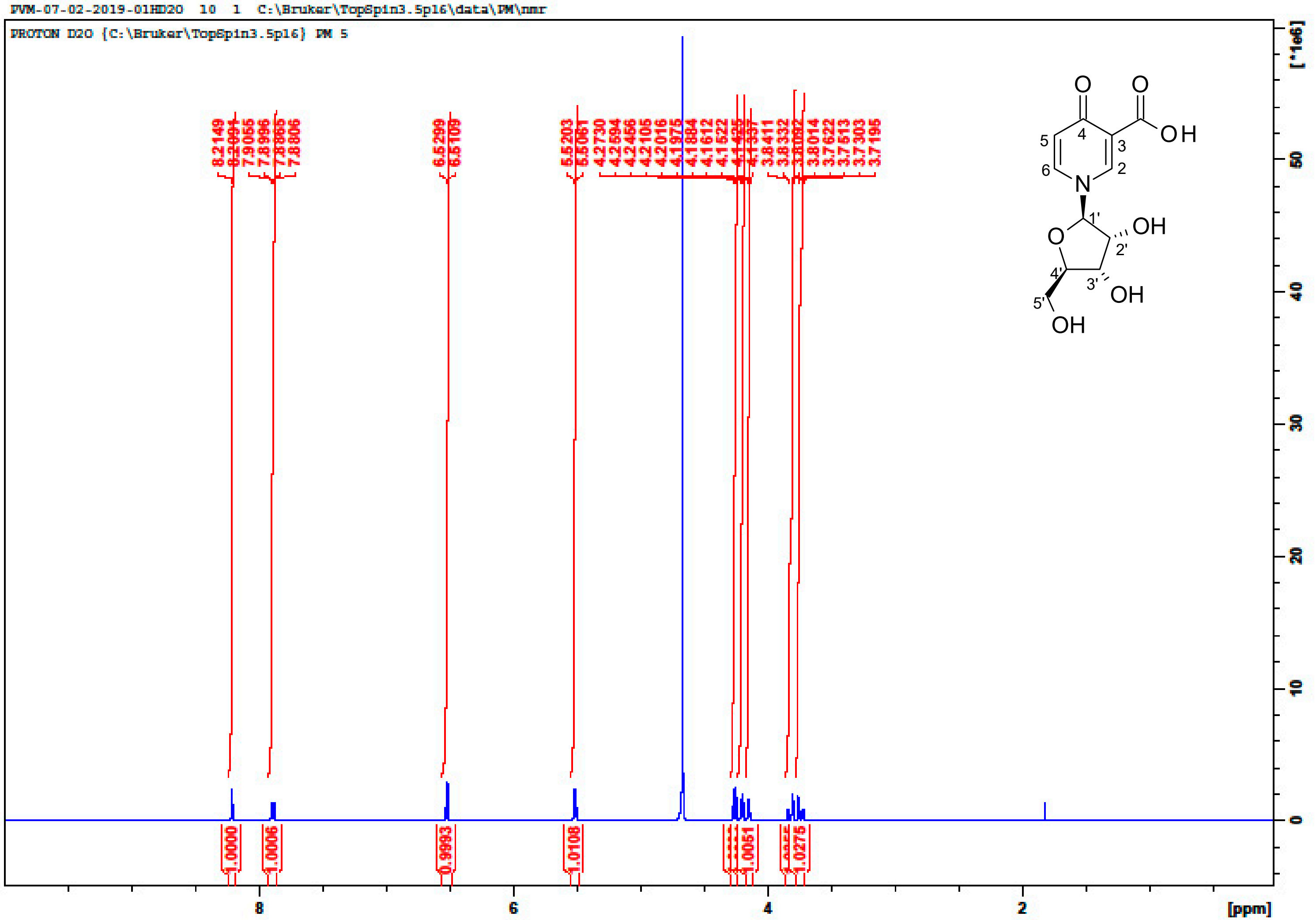

Compound 26. 400 MHz ^1H NMR spectrum in D_2O

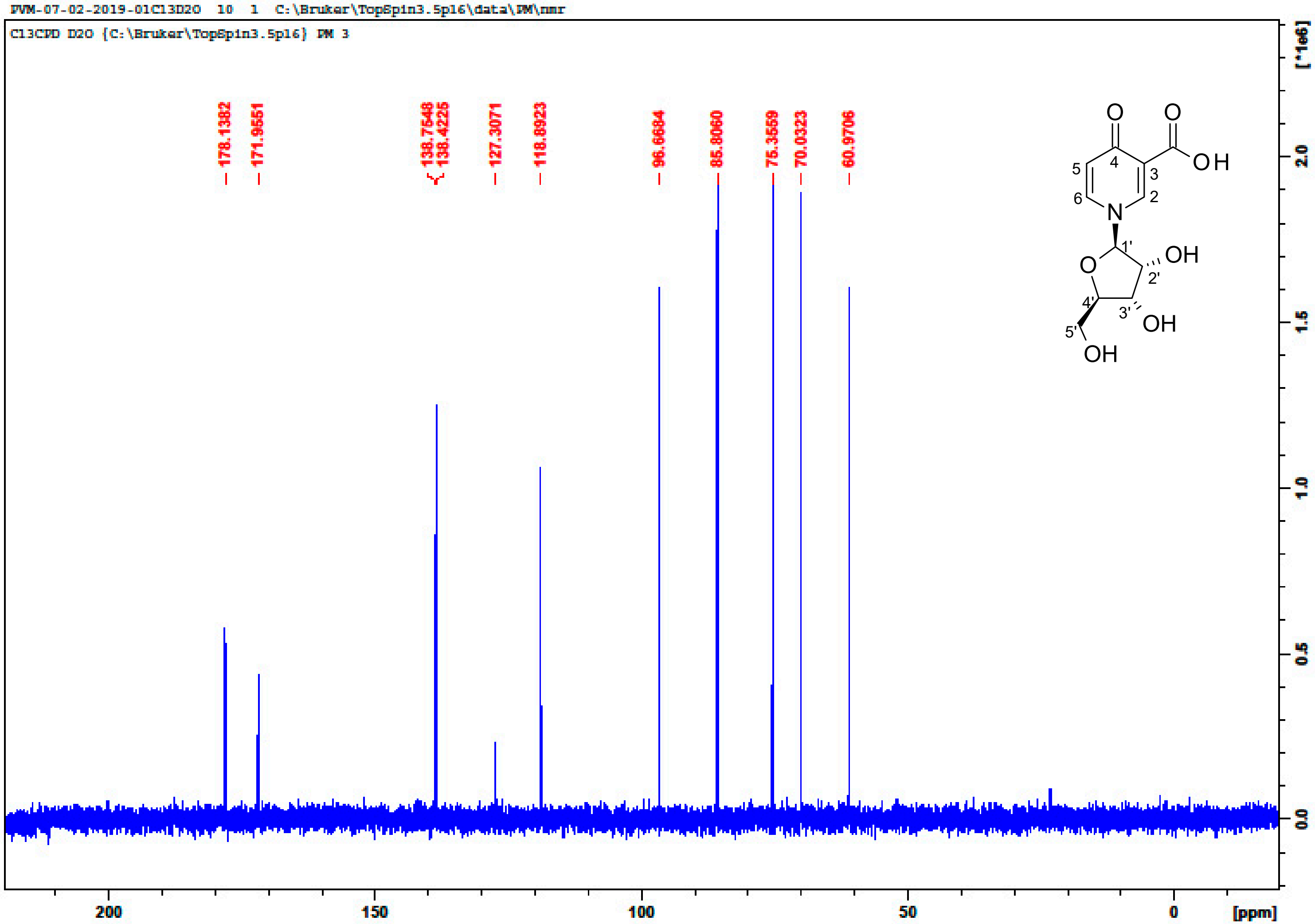

Compound 26. 400 MHz ^{13}C NMR spectrum in D_2O

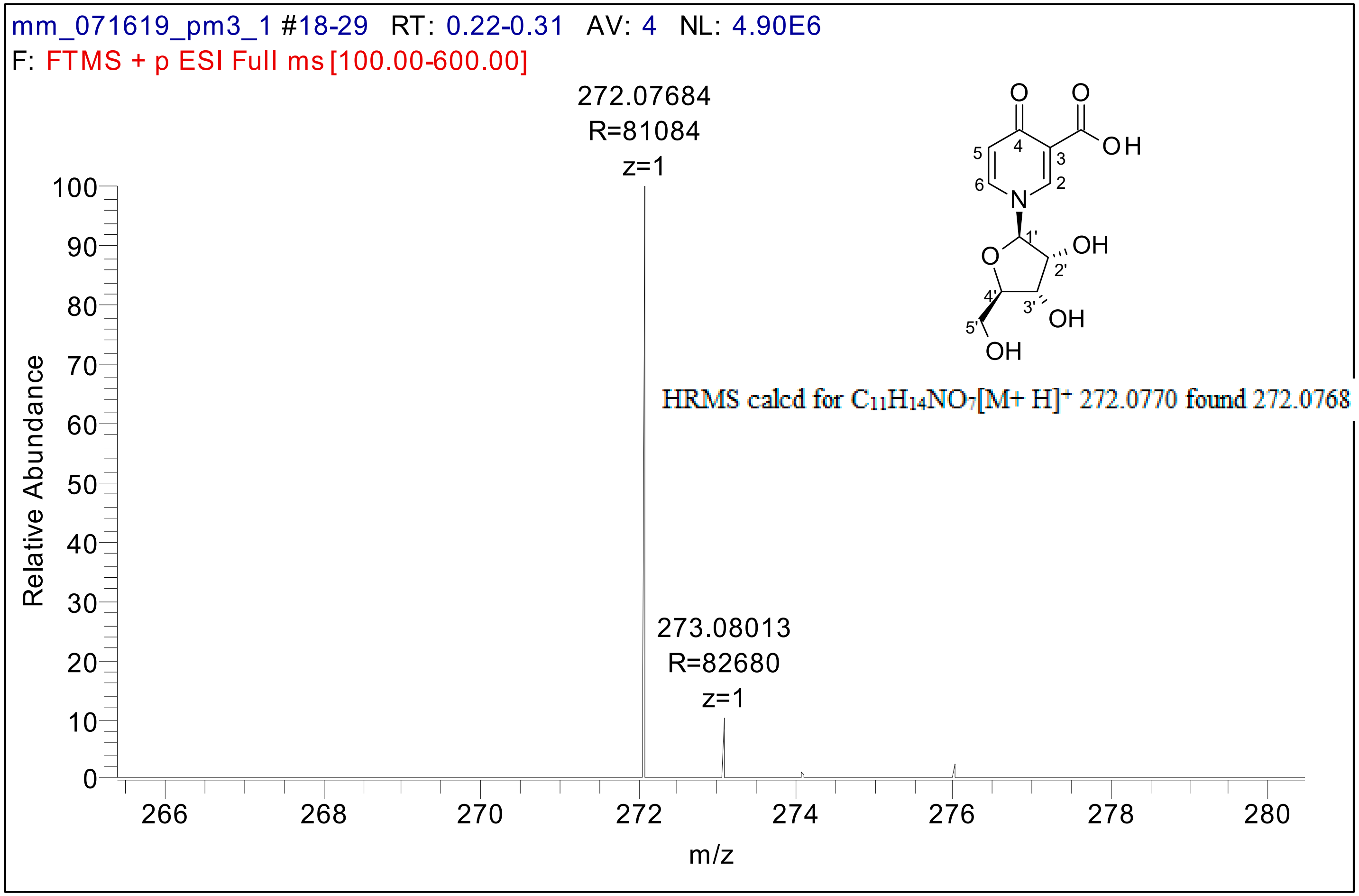

Compound 26. HRMS spectra

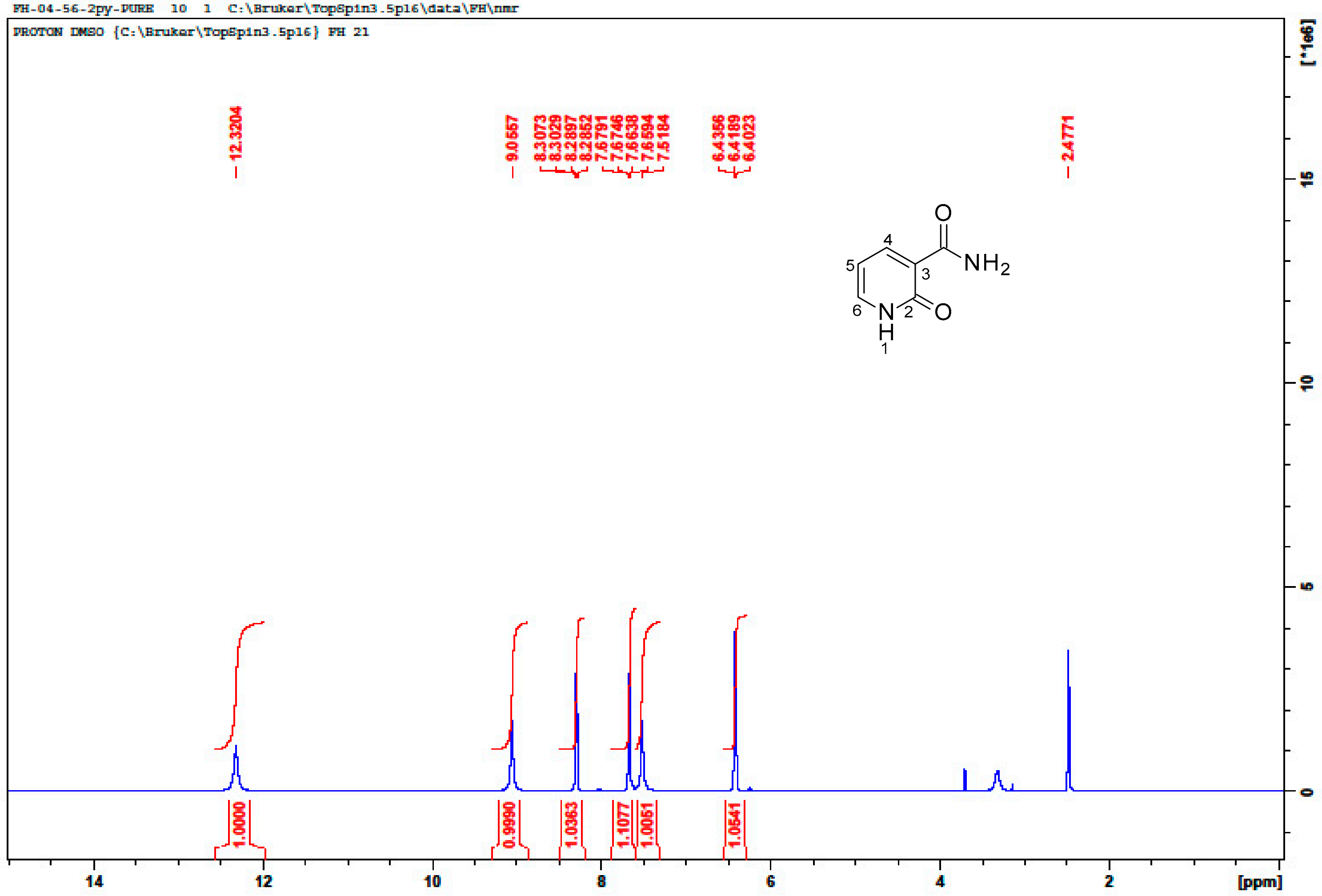

Compound 29. 400 MHz ^{1}H NMR spectrum in D_2O

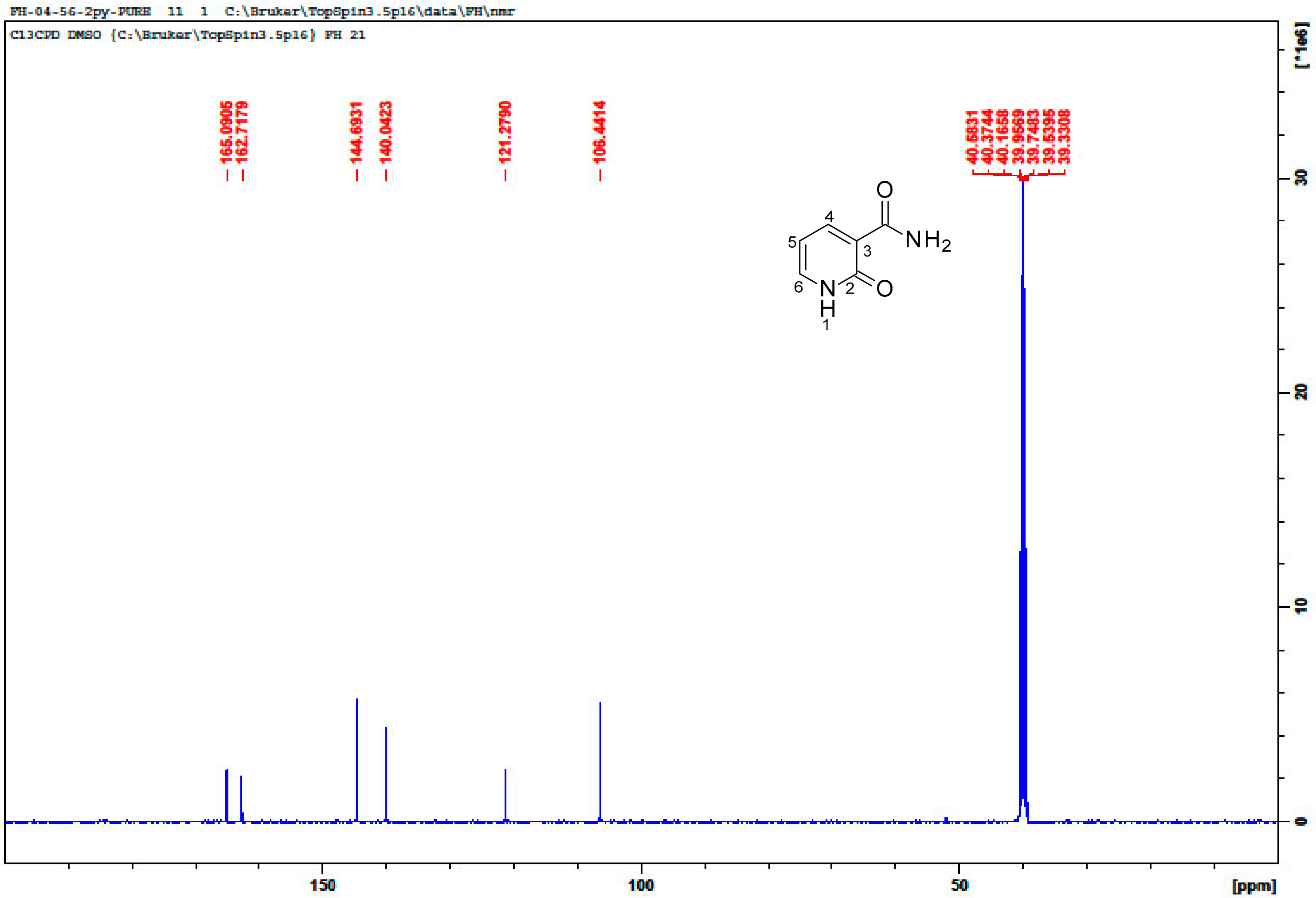

Compound 29. 400 MHz ^{13}C NMR spectrum in D_2O

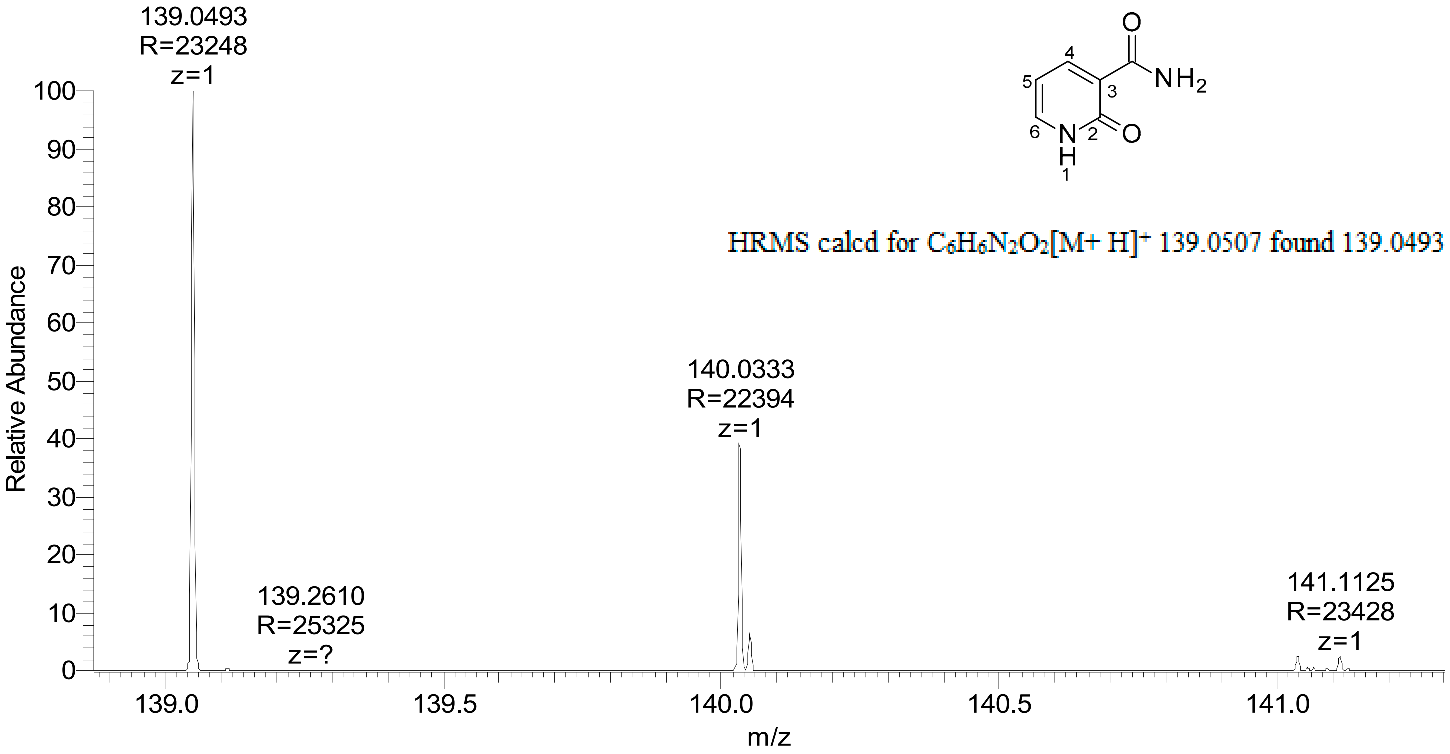

Compound 29. HRMS spectra

Figure 2: NQO2 catalyzed 4PYR formation

4PYR was observed from the reaction of NRH + NQO2, but not NR + NQO2.

Analyte	Parent Ion m/z	Major Fragment Ion	RT
NR	255.10	123.06	22.7
NRH	257.11	167.08	10.5
4PYR	271.09	139.05	10.8

XIC: 4PYR Observed in NRH + NQO2 Fraction 1

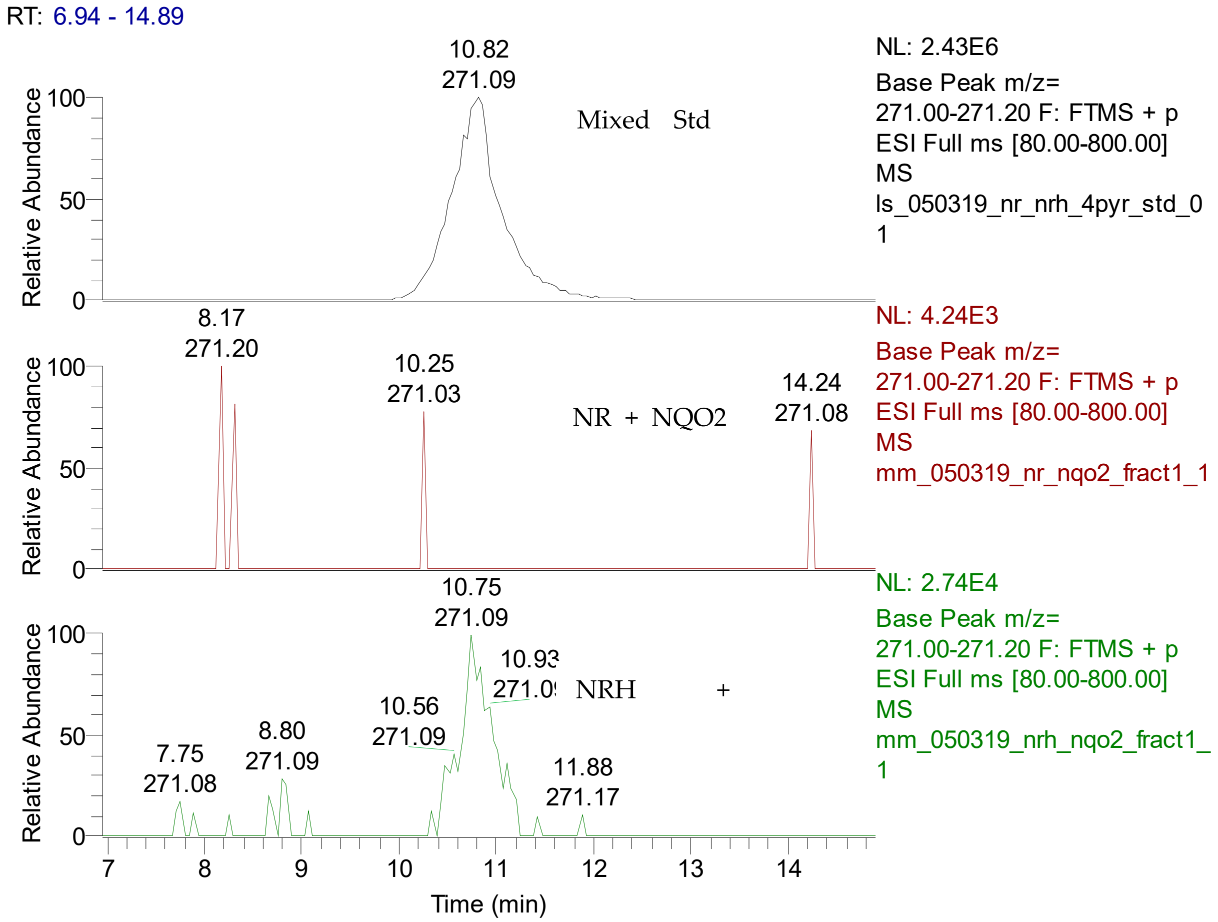

Targeted MS2 Spectrum of 4PYR

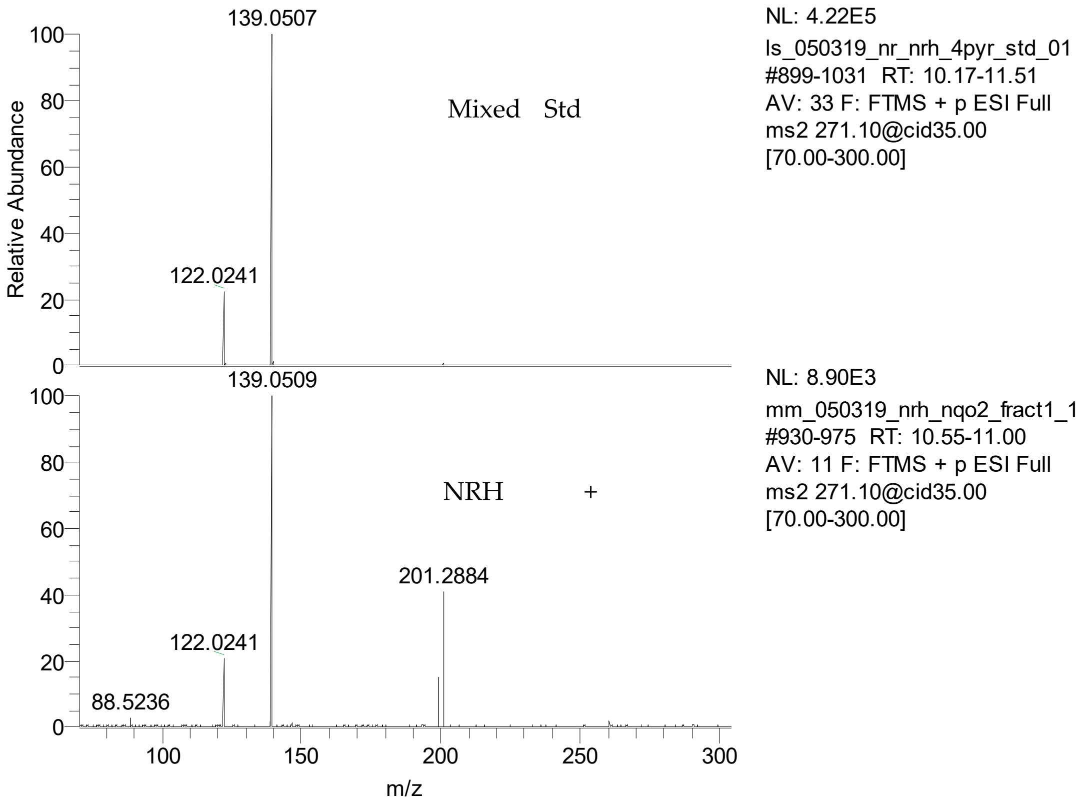

Figure S3: Fold change in human urinary NAD catabolites following 1gm administration of acetaminophen/APAP (average n=2, and technical replicates).

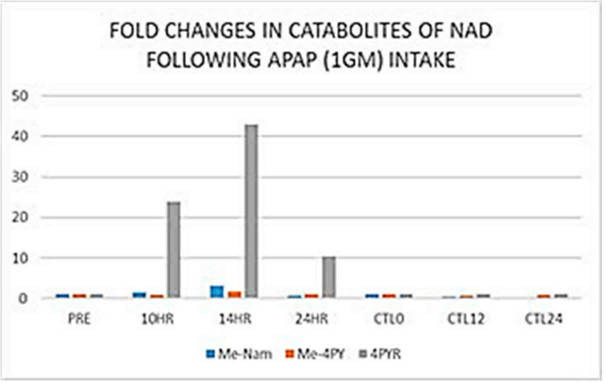

Supplement: Supplementary file 1 [file ijms-22-01145-s001.pdf]
